# Brainstem and cerebellar radiological findings in progressive supranuclear palsy

**DOI:** 10.1093/braincomms/fcaf051

**Published:** 2025-02-05

**Authors:** Chloe Spiegel, Cassandra Marotta, Kelly Bertram, Lucy Vivash, Ian H Harding

**Affiliations:** Department of Neuroscience, School of Translational Medicine, Monash University, Melbourne 3004, Australia; Department of Neurology, Alfred Health, Melbourne 3004, Australia; Department of Neuroscience, School of Translational Medicine, Monash University, Melbourne 3004, Australia; Department of Neuroscience, School of Translational Medicine, Monash University, Melbourne 3004, Australia; Department of Neurology, Alfred Health, Melbourne 3004, Australia; Department of Neuroscience, School of Translational Medicine, Monash University, Melbourne 3004, Australia; Department of Neuroscience, School of Translational Medicine, Monash University, Melbourne 3004, Australia; QIMR Berghofer Medical Research Institute, Brisbane 4006, Australia

**Keywords:** progressive supranuclear palsy, brainstem, cerebellum, magnetic resonance imaging, positron emission tomography

## Abstract

Progressive supranuclear palsy is a sporadic neurodegenerative 4-repeat tauopathy associated with significant morbidity. Heterogeneity of symptom expression among this group is increasingly recognized, reflecting variable tau spread and neurodegeneration. Clinical manifestations consist of debilitating and rapidly progressive motor, oculomotor, speech, cognitive and affective impairments. Core pathological changes are noted with a predominance in the midbrain and basal ganglia; however, spread to the more caudal brainstem and cerebellar regions is reported at various stages. Accordingly, whilst midbrain atrophy is the best recognized supportive imaging finding, quantitative neuroimaging studies using MRI and PET approaches have revealed a wider profile of brain abnormalities in cohorts of individuals with progressive supranuclear palsy. This expanded neurobiological scope of disease may account for individual heterogeneity and may highlight additional biological markers that are relevant to diagnosing and tracking the illness. Additionally, there is increasing understanding of the diverse cognitive, affective and speech functions of the cerebellum, which may be implicated in progressive supranuclear palsy beyond current recognition. In this review, we undertake a systematic literature search and summary of *in vivo* structural and functional neuroimaging findings in the brainstem and cerebellum in progressive supranuclear palsy to date. Novel and multimodal imaging techniques have emerged over recent years, which reveal several infratentorial alterations beyond midbrain atrophy in progressive supranuclear palsy. Most saliently, there is evidence for volume loss and microstructural damage in the pons, middle cerebellar peduncles and cerebellar cortex and deep nuclei, reported alongside recognized midbrain and superior cerebellar peduncle changes. Whilst the literature supporting the presence of these features is not unanimous, the evidence base is compelling, including correlations with disease progression, severity or variant differences. A smaller number of studies report on abnormalities in MRI measures of iron deposition, neuromelanin, viscoelasticity and the glymphatic system involving the infratentorial regions. Molecular imaging studies have also shown increased uptake of tau tracer in the midbrain and cerebellar dentate nucleus, although concern remains regarding possible off-target binding. Imaging of other molecular targets has been sparse, but reports of neurotransmitter, inflammatory and synaptic density alterations in cerebellar and brainstem regions are available. Taken together, there is an established evidence base of *in vivo* imaging alterations in the brainstem and cerebellum which highlights that midbrain atrophy is often accompanied by other infratentorial alterations in people with progressive supranuclear palsy. Further research examining the contribution of these features to clinical morbidity and inter-individual variability in symptom expression is warranted.

## Teaser

Progressive supranuclear palsy is a neurodegenerative tauopathy associated with significant morbidity. Tau spread frequently implicates the brainstem and cerebellum; however, studies of *in vivo* imaging have focused primarily on characteristic midbrain atrophy. In this review, Spiegel *et al.* summarize the continuum of radiological infratentorial findings reported in this disease.

## Graphical Abstract

**graphical abstract:**
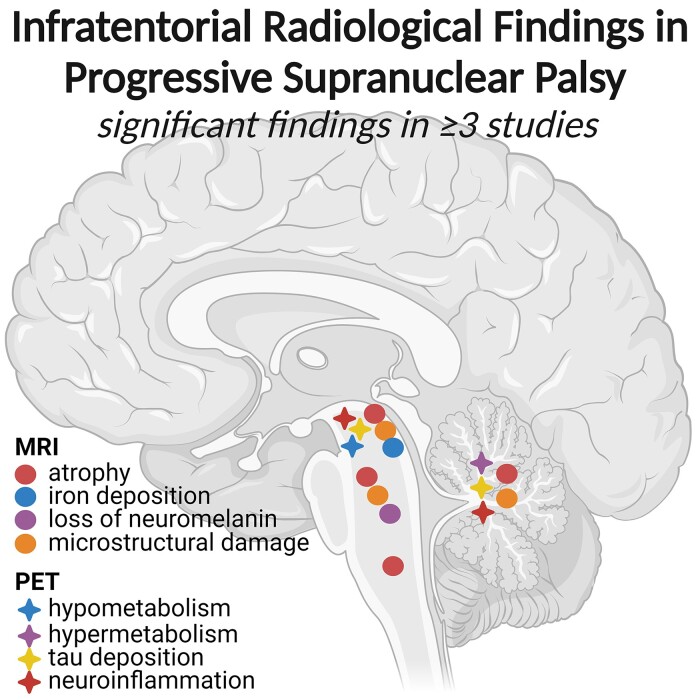

## Introduction

Progressive supranuclear palsy (PSP) is a sporadic neurodegenerative 4-repeat (4R) tauopathy associated with significant morbidity. It commonly presents with progressive vertical gaze dysfunction, rigidity, dysarthria and postural instability, along with a range of other manifestations, and is considered one of the atypical parkinsonian syndromes.

The diagnosis of PSP currently relies predominantly on clinical findings, in accordance with the Movement Disorder Society PSP diagnostic criteria.^1^ Diagnosis after first symptom presentation is often delayed and patients can be misdiagnosed as having Parkinson’s disease, particularly in the early stages.^2^ Symptom heterogeneity in PSP may further contribute to diagnostic uncertainty. Richardson syndrome (PSP-RS) is the classical and most common form of PSP, where supranuclear vertical gaze palsy and postural instability are central features.^1^ Multiple variant presentations of PSP are also now recognized, including PSP with predominant parkinsonism (PSP-P) where a vertical gaze palsy can be seen alongside asymmetric parkinsonism that is more characteristic of Parkinson’s disease.^2^ This phenotype, in particular, may be confused with Parkinson’s disease early on. Moreover, clinical variability is increasingly recognized, with rare overlap variants, such as PSP with Primary Lateral Sclerosis (PSP-PLS) reported.^3^ This complexity and considerable delay in diagnosis undoubtedly has implications for prognostication, counselling and patient selection for clinical trials. Many studies have therefore attempted to identify a reliable diagnostic imaging biomarker.

**Figure 1:**
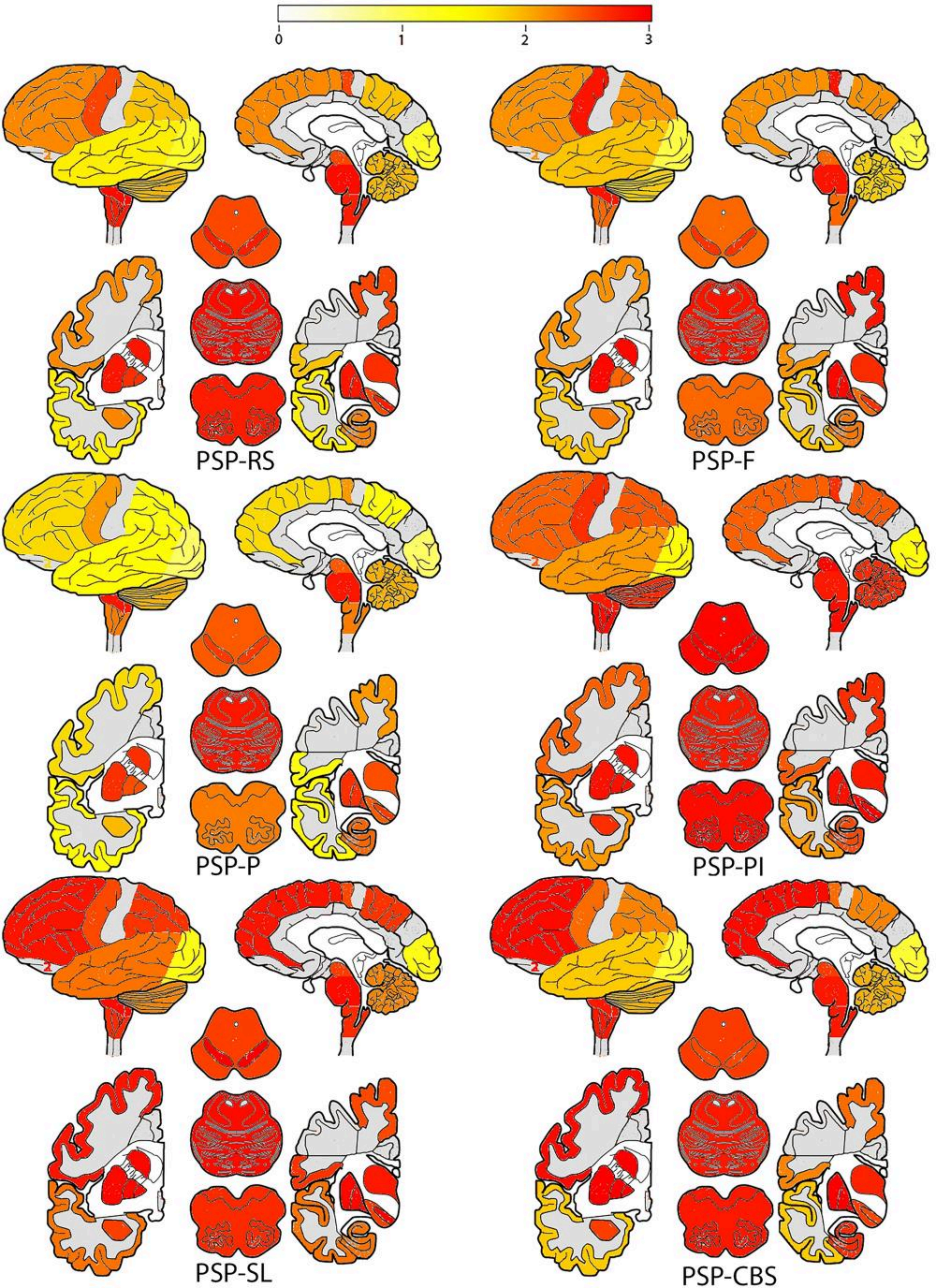
**Pathological hallmarks of PSP in neocortex, basal ganglia, brainstem and cerebellum in PSP**. Illustrative heat maps of tau aggregation in PSP variants. Top images display the lateral and medial surfaces of the brain. Bottom images to the left and right display coronal brain slices capturing the basal ganglia, with three axial representations of the brainstem in the middle at the level of the midbrain, pons and medulla in descending order. Scale: white (lighter) = none, through yellow and orange, to red (darker) = severe. Image reproduced with permission from Kovacs *et al*.^7^ PSP-RS = PSP-Richardson syndrome; PSP-F = PSP-frontal variant; PSP-P = PSP-Parkinsonism; PSP-PI = PSP-postural instability, PSP-SL = PSP speech-language variant; PSP-CBS = PSP-corticobasal syndrome.

Midbrain atrophy, measured by the magnetic resonance Parkinsonian index (MRPI) or midbrain–pons area ratio, is considered supportive of an early diagnosis of PSP-RS or PSP-P.^4^ The newer magnetic resonance Parkinsonian index 2.0, which also includes third ventricle width, may further improve accuracy for distinguishing PSP-P from Parkinson’s disease.^5^ However, none of these midbrain measures consistently corresponds with PSP tau pathology at post-mortem.^4^ Tau pathology in the brainstem and basal ganglia is considered mandatory for a pathological diagnosis of PSP^6^ but is also commonly reported in the cerebellum^7,8^; Fig. 1. Cerebellar dysfunction contributes to a number of neurological diseases,^9-11^ even when ataxia is not a presenting feature. Parkinson’s disease is an important example, where morphometric and functional imaging have demonstrated changes in the cerebellum corresponding to motor and non-motor symptoms, suggesting possible pathological or compensatory modifications.^12,13^ Similarly, tau accumulation in the dentate nucleus of the cerebellum is common to all PSP variants, including the rare PSP with predominant cerebellar ataxia variant, which may contribute to the motor, cognitive and affective symptoms, beyond current recognition.^14^ Critically, variable involvement of the cerebellum and other subtentorial structures across individuals may help explain the significant variability in symptom manifestation.

Over recent decades, imaging methods and algorithms have continued to advance. Studies of PSP have attempted to characterize morphology and function through the use of multiple neuroimaging modalities. These include CT,^15^ MRI,^16-19^ proton magnetic resonance spectroscopy^20-22^ PET,^23-25^ single photon emission computed tomography (SPECT)^26,27^ and transcranial sonography. The diagnostic utility of these modalities was reviewed in 2017 by Whitwell *et al*.^4^ As noted, many studies have focused on midbrain atrophy.^18,28,29^ Less is known about other findings in the brainstem or cerebellar regions, and their relationship with the clinical features.

In this review, we undertake a systematic literature search and summary of *in vivo* structural and functional neuroimaging findings in the brainstem and cerebellum in PSP, including relevant clinical associations and the potential applicability of these measures in research and clinical settings.

## Methods

A systematic literature search of the MEDLINE, PubMed and EMBASE databases was performed according to PRISMA guidelines using the following keywords: ‘progressive supranuclear palsy,’ or ‘Steel Olszewski Richardson syndrome,’ or ‘progressive supranuclear opthalmoplegia.’ This search was combined with a search of anatomical keywords including: ‘brainstem,’ ‘cerebellum,’ ‘cerebellar peduncles,’ ‘midbrain,’ ‘mesencephalon,’ ‘pons,’ ‘medulla’ and ‘rhombencephalon’. The database search included studies published as of 23rd June 2023.

Abstracts were screened for inclusion by two reviewers and conflicts resolved by consensus between the reviewers. Full texts were then further screened by the first author (C.P.) to determine final inclusion. Criteria for inclusion:

- A study population with possible, probable or definite PSP diagnosed by a movement disorders neurologist, and/or according to the specific National Institute of Neurological Disorders and Stroke Society for PSP or movement disorder society PSP criteria.
- Structural or functional imaging of the brain including the brainstem and/or cerebellum using any of: CT, MRI, proton magnetic resonance spectroscopy, PET, SPECT and transcranial sonography.
- Quantitative comparison between a PSP cohort and a healthy control or alternative disease group.
- Case–control studies, randomized control trials or meta-analyses.
- Available in English and published in a peer-review journal.

Criteria for exclusion:

- A study population of atypical parkinsonian syndromes where a distinct PSP group was not identified
- Studies of genetic or familial PSP
- Case reports or case series
- Studies of a PSP group that have undergone brain surgery

**Figure 2:**
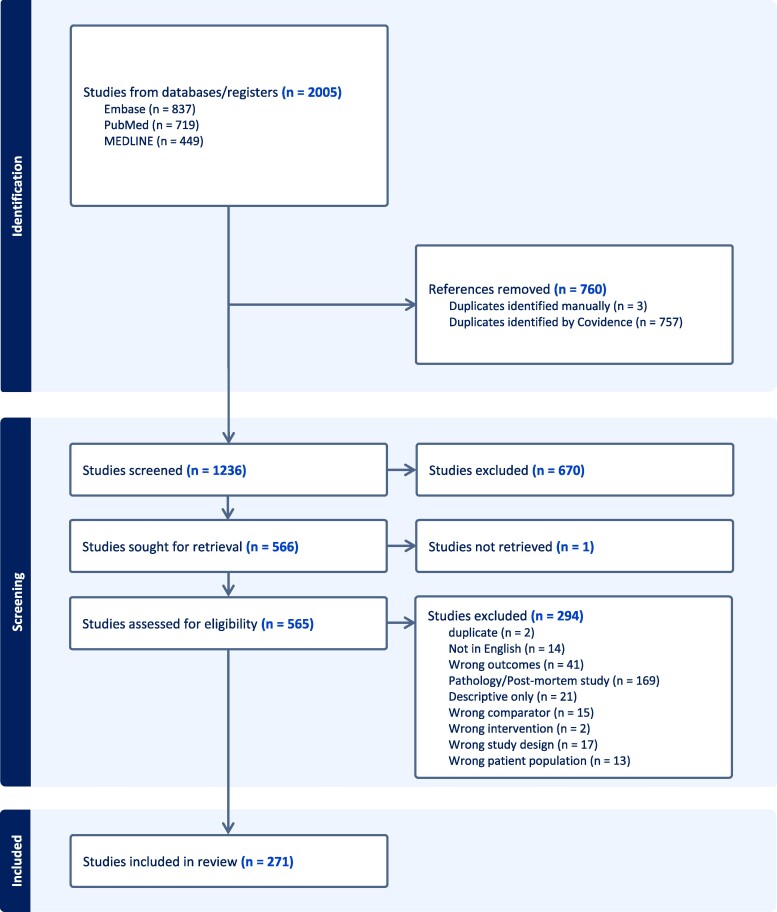
**PRISMA chart.** Produced with Covidence systematic review software, Veritas Health Innovation,Melbourne, Australia. Available at www.covidence.org.

Study selection and reasons for exclusions are summarized in the PRISMA chart in Fig. 2. The initial search aimed to include both imaging and neuropathology studies; however, the scope was narrowed and post-mortem studies without *in vivo* imaging were subsequently excluded.

## Results

Two hundred and seventy-one studies were reviewed. The study populations, some demographic data (i.e. disease duration), the imaging methods and main findings for each were individually reviewed and recorded in Supplementary Table 1. Of these, 63 MRI studies reported on recognized midbrain atrophy measures, such as midbrain diameter, area, volume, midbrain/pons ratio and magnetic resonance Parkinsonian index 1.0 or 2.0. As these measures are well-recognized, and have been reviewed previously,^4,30^ we briefly summarize these findings in Supplementary Table 2. Findings from the remaining studies utilizing MRI, PET, magnetic resonance spectroscopy, transcranial sonography, SPECT or CT imaging are described below. Nine studies were performed longitudinally.

Studies using fMRI are relatively limited in number (*n* = 12). Due to the heterogeneity in study designs, methodologies, and regions studied it was not possible to provide a clear concise summation or interpretation with regard to posterior fossa structures. A summary of these studies can be found in Supplementary Table 3.

## Brainstem imaging

### Conventional and volumetric MRI

Brainstem changes are summarized in Table 1. Brainstem atrophy is well recognized in PSP. In contrast with consistent evidence of midbrain atrophy,^29,178-180^ atrophy of the pons and medulla is less certain, with similar numbers of studies supporting and refuting these findings.^31,40-42^ Highlighting the inconsistency, one meta-analysis reported significant white matter atrophy in the pons compared with controls,^40^ while another earlier meta-analysis comparing PSP with Parkinson’s disease did not.^43^ Two studies report worsening pontine volume loss alongside midbrain atrophy on longitudinal 12-month imaging.^44,45^ This was not replicated in a later voxel-based study, using a shorter 6-month interval.^181^ Highlighting the relevance to disease severity, one longitudinal study found a correlation between volume loss in both the midbrain and the pons, and worsening PSP rating scale (PSPRS) scores.^45^

**Table 1:** Brainstem findings in the literature

			Midbrain	Midbrain nuclei				Pons	Pontine nuclei		Medulla
		Unspecified region		SN	RN	SC/IC	CN		PPN	LC	
MRI											
Atrophy	+	^31-39^	^31-33,40-76^	^32,33,48,77-82^	^48,57^			^31,40-42,44-46,49,51,54-58,60,61,64,68,73,75^			^41,42,45,52,53,56,59,60,68^
	−	^83,84^						^33,43,47,48,53,62,63,65-67,69-72,74,85,86^			^31,33,40,43,46-48,58,61,62,65,67,69-73,85^
Microstructural/neuronal damage	+	^87,88^	^22,32,46,49,50,61,71,89-98^	^32,99-103^	^77,93,100,101^			^22,32,50,61,89,91,93,95,102,104,105^	^32,91,100,101^	^32^	
	−		^102,106,107^					^46,49,71,96,106,108-112^			^46,49,50,61,71,91,95,102^
Iron deposition	+		^113^	^114-123^	^114-116,118,119,121,124^						
	−			^124^							
↓ neuromelanin	+			^80,125^						^82,126,127^	
	−			^128^						^128^	
↓ viscoelasticity	+		^129^								
PET											
↓metabolism	+	^130^	^24,25,108,110-112,131-145^			^139^	^135^		^135^		^108,130^
	−							^24,131-133,136-143,145^			^24,131-133,136-143,145^
↑ tau	+		^33,48,69,144,146-156^	^146,149,154^	^33^			^146^		^146 a^	
	−		^157^	^158^				^33,48,69,148-151,154^		^33,48,69,150,154^	^33,69,149,150,154^
↑ 5HT2A receptors	+			^159^							
↓ monoamine transporter	+		^160^								
↓ Acetylcholine	+		^161,162^			^161^					^161 b^
Neuroinflammation	+		^148,163^					^163^			^163^
	−		^160^					^160^			^160^
↓ neuromelanin	+			^164^							
↓ synaptic density	+		^165^					^165^			^165^
↓ BBB efflux	−		^166^					^166^			^166^
Ultrasound/CT											
↓ area/width	+		^167-169 c^	^170,171^							
↓ echogenicity	+			^167,172^							
↑ echogenicity	−			^171,173^							
SPECT											
↓ perfusion	−	^174,175^									
↓ monoamine transporter	+		^176,177^					^176^			

^a^Includes Raphe Nuclei.

^b^Includes Inferior Olivary Nucleus.

^c^Single CT study included in this section.

‘+’ rows list studies with significant findings compared with control or disease group ‘−’ rows list studies without significant findings compared with control or disease group. SN = substantia nigra; RN = red nucleus; SC = superior colliculi; IC = inferior colliculi; CN = cunieform nucleus; PPN = pedunculopontine nucleus; LC = locus coeruleus; BBB = blood brain barrier.

The literature is heavily focused on PSP-RS, in which midbrain atrophy is recognized. There are, however, recent reports of midbrain atrophy in a number of variants as well. These include PSP-P, PSP-F, PSP-CBS, PSP-PGF and PSP-SL.^31,46-48^ Despite longer disease duration in such variants, midbrain volume loss appears to be greater in PSP-RS.^48^ No volumetric differences across variants have been reported in the pons or medulla.^48^

### Microstructural MRI

Microstructural MRI has the theoretical potential to reveal neurodegenerative pathology prior to the development of apparent atrophy on conventional MRI. Studies have attempted to characterize the microstructural and white matter tract changes in PSP using magnetic resonance spectroscopy^49^ and diffusion-weighted imaging,^104,182^ including diffusion tensor imaging (DTI),^183,184^ diffusion kurtosis imaging^89,90^ and free water imaging.^99,100^

Similar to the volumetric findings, microstructural changes are predominantly observed in the midbrain, with more limited evidence in other brainstem regions.^32,46,50,91^ DTI typically shows reduced fractional anisotropy or increased mean diffusivity in affected regions compared with controls or other disease groups.^46,91-93^ Similarly, two studies of diffusion kurtosis imaging have shown decreased midbrain kurtosis.^89,90^ One study also reported that midbrain fractional anisotropy was significantly reduced in PSP-RS compared with PSP-P.^46^ Studies of DTI and free water methods show changes fairly consistently in the substantia nigra and red nucleus.^93,99-102^ DTI tractography studies have also demonstrated alterations in the dentatorubrothalamic tract (DRTT).^46,93^ Despite microstructural techniques appearing to distinguish PSP from other groups with reasonable accuracy,^32,93^ midbrain findings have not been reported in the absence of significant atrophy^46,50^ and do not seem to surpass midbrain volume as a tool for diagnosing PSP-RS.^32^

There have been mixed reports of microstructural change in the pons. Pyatigorskaya *et al.*^32^ report diffusion changes in specific pontine nuclei, including the pedunculopontine nucleus and locus coeruleus, among other regions. Although not exceeding the diagnostic accuracy of midbrain volume, pontine fractional anisotropy was also one of the best distinguishing markers of PSP-RS compared with Parkinson’s disease.^32^ Some studies have shown similar diffusion changes in the pons,^50,61,89,93^ and dedicated free water imaging studies have also implicated the pedunculopontine nucleus.^100,101^ Despite these reports, such findings have not been consistent across studies.^46,49,96,109^ No studies have shown microstructural changes in the medulla.

### Neuromelanin sensitive MRI

The neuromelanin-related pigmentation of dopaminergic and noradrenergic neurons in specific nuclei can become depleted with neurodegeneration. Dedicated neuromelanin-sensitive MRI has been used to assess the substantia nigra and locus coeruleus.^80,125-127^ Depigmentation of the substantia nigra, associated with nigral degeneration, has been reported in a number of studies of Parkinson’s disease.^185,186^ More recently, it has been shown that depigmentation and volume loss of the pars compacta also occurs in PSP.^80,125,126^ Topographical differences in the pattern of nigral depigmentation have also been reported, though these have not aligned across studies.^80,125^ Whilst midbrain volume remains a superior diagnostic tool, one study showed improved diagnostic accuracy in distinguishing PSP from Parkinson’s disease when combining volumetry with neuromelanin sensitive MRI.^80^

Similarly, the locus coeruleus, a key region involved in arousal, has been studied with variable results.^126-128^ A recent study using 7-T ultrahigh field magnetization transfer imaging showed significant contrast reduction in the caudal locus coeruleus in both Parkinson’s disease and PSP, compared with controls.^126^ These changes were associated with worsening cognition and apathy.^126^ There were no significant differences between the Parkinson’s disease and PSP groups however,^126^ which is at odds with 3T MRI studies showing significantly reduced contrast signal in Parkinson’s disease or multisystem atrophy compared with PSP.^82,128^ Post-mortem studies have shown both tau and synuclein-related neurodegeneration in the locus coeruleus,^187,188^ though the locus coeruleus does appear to be relatively spared in PSP.^82^

### Other MRI measures

One study used a region-of-interest approach to assess brain elasticity, and found that using magnetic resonance elastography, the midbrain viscoelasticity was significantly reduced in PSP compared with a control and Parkinson’s disease group.^129^ This highlights changes in the microarchitecture, which correlate with midbrain volume loss.

**Figure 3:**
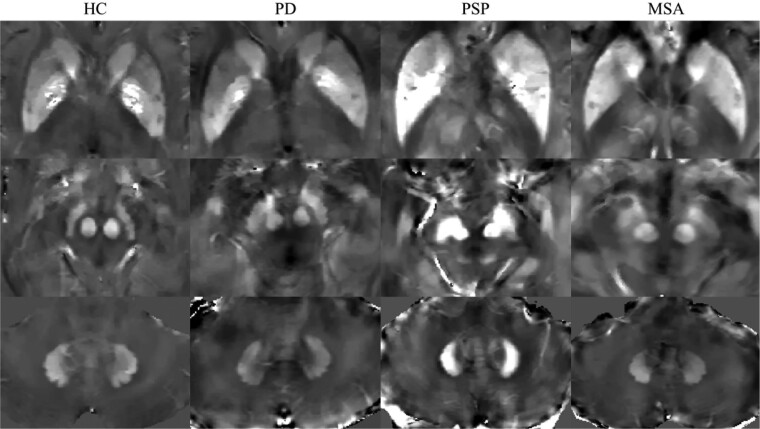
**Quantitative susceptibility mapping in the basal ganglia and brainstem.** Quantitative susceptibility mapping showing increased susceptibility in the basal ganglia (top); substantia nigra and red nucleus (middle), and cerebellar dentate nucleus (bottom) in a representative individual with PSP relative to HC, Parkinson’s disease and MSA. Reproduced with permission from Zhang *et al*.^115^ HC = healthy controls; PD = Parkinson’s disease; MSA = multisystem atrophy.

Signal changes suggesting iron deposition in the substantia nigra and red nucleus have been reported in several studies using dedicated MRI sequences, such as susceptibility weighted imaging or quantitative susceptibility mapping.^114-116,118,120-122,124^ A number of these studies have shown that there tends to be significantly higher susceptibility in the substantia nigra or red nucleus in PSP,^114-116,118,119^ distinguishing PSP from Parkinson’s disease or multisystem atrophy with good accuracy^115,116,119^ (Fig. 3). Another study showed that pairing susceptibility in the red nucleus with established morphometric midbrain measures improved the differentiation of PSP from Parkinson’s disease (AUC 0.976).^116^

Several MRI studies have also shown alterations in CSF flow and drainage. A recent study using DTI analysis along the perivascular space found a significantly reduced DTI analysis along the perivascular space index throughout the brainstem, suggesting impairment of the glymphatic system in this region.^189^ Another study reported significant enlargement of brainstem perivascular spaces in PSP compared with controls.^190^ As the glymphatic system and perivascular spaces are thought to play a pivotal role in the clearance of solutes, dysfunction of this system is proposed to contribute to the accumulation of proteins in neurodegenerative disease. Additionally, Fukui *et al.*^191^ used a CSF flow study to show significantly reduced mean velocity, along with midbrain atrophy and aqueduct dilatation, in PSP compared with Parkinson’s disease. Using the width of the CSF velocity and area of the midbrain aqueduct, they were able to differentiate the PSP group from Parkinson’s disease group with reasonable accuracy (93.3%).^191^

### Ultrasound and CT

Only one study of CT met the inclusion criteria for this review, likely due to the exclusion of qualitative case series. This study demonstrated a reduced midbrain anterior–posterior diameter, which is concordant with midbrain atrophy measured on MRI.^169^ Few studies of transcranial sonography have also demonstrated a reduced midbrain area.^167,168^ When looking at the substantia nigra specifically, there are reports of reduced size and hypoechogenicity,^167,170,171^ though this does not clearly distinguish PSP from Parkinson’s disease.^167^ Highlighting this, a small study in 2010 found that hyperechogenicity, which is typically a feature of Parkinson’s disease, was present in six out of seven PSP-P patients, and one of the 27 PSP-RS patients.^192^ No studies of transcranial sonography have interrogated other brainstem regions.

### Metabolic PET

Significant midbrain hypometabolism measured by fluorodeoxyglucose (FDG) PET is observed in PSP-RS and PSP-P.^131,132,134,136^ Interestingly, such hypometabolism was not seen in a study of PSP-PAGF.^110^ Only a single study, which used a region-of-interest approach to investigate the supraspinal locomotor centres, has reported hypometabolism in in the cuneiform complex and pedunculopontine nucleus, located in the upper pons.^135^ Other studies that used a whole-brain statistical parametric mapping approach did not find metabolic changes in the pons or medulla.^132,133,136^ In a study from 2014, the ‘pimple sign,’ characterized by a focal region of hypometabolism in the midbrain, was found to have high specificity, but low sensitivity for PSP. Subsequent studies have shown improved sensitivity when patterns of hypometabolism in both the supratentorial and infratentorial regions are considered.^134,141,193^

### Tau PET

A more recent and exciting development is the use of tau-specific PET ligands to assess *in vivo* tau deposition. A number of tau tracers have been investigated. The majority have used 18F-flortaucipir (also known as 18F-AV-1451), frequently reporting increased uptake in the midbrain.^33,48,147,149,151,152,154^ A single longitudinal study using this tracer did not find any significant regional change over time^151(p201)^. 18F-flortaucapir uptake has also been shown to correlate with regional MRI volumes;^33^ however, there remains some uncertainty as to how effectively it truly binds 4R-tau. This uncertainty stems from autoradiography reports of relatively low affinity,^194,195^ as well as regions of off-target binding in a number of *in vivo* studies.^33,154^ In an attempt to optimize this approach, novel tau tracers have emerged in recent years, though potential off-target binding remains a concern. As an example, Oliveira Hauer *et al.*^158^ recently investigated a novel tracer, 18F-RO-948, but found that there was actually increased binding in the substantia nigra in the healthy control group. The authors suggest that this is due to an affinity for neuromelanin, which appears to be reduced in PSP.^164^

A different novel tracer, F-APN-1607, is suggested to have less off-target binding, although higher background activity and binding in the choroid plexus has still been observed.^146^ Despite this concern, it is worth noting that one study showed increased uptake in the pons, specifically the pontine base, raphe nuclei and locus coeruleus in PSP.^146^ Adding weight to this finding, they also showed that PSPRS correlated with uptake in the pontine base, along with the midbrain and specific nuclei.^146^ Notably, however, this has not been replicated in other studies.^33,48,69,148-151,154^

Only one study reported increased tracer uptake in the medulla, along with the midbrain.^153^ In this study, a voxel-wise approach was used to investigate 18F-THK5351 binding in PSP and healthy controls^153(p201)^. Although, initially thought to have potential as a tau tracer, 18F-THK5351 has been shown to have a greater affinity for monoamine oxidase B, a marker of astrogliosis.^148^ This finding is therefore of uncertain significance, and may reflect more extensive neuroinflammation in the brainstem. No other tau tracer studies have found significantly increased uptake in the medulla.^33,69,149,150,154^

### Other PET ligands

As noted above, certain ligands may be used to assess neuroinflammation. In addition to reports of increased 18F-THK5351 brainstem uptake,^148,153^ a different ligand targeting microglia, 11C(R)-PK11195 has been studied.^160,163^ An early study showed increased uptake in the midbrain among other regions,^163^ however this did not significantly change on the 6 and 10-month analysis, and was not replicated in a larger study of sixteen PSP-RS patients.^160^ Interestingly, despite the inconsistency, midbrain and pons 11C(R)-PK11195 uptake correlated with the PSPRS score in the larger study.^160^

Additional studies have investigated specific neurotransmitters. Using 18F-altanserin, post-synaptic serotonin (5-HT2A) receptors were found to be upregulated in the substantia nigra.^159^ Cholinergic deficit in the brainstem has also been reported using 18F-FEOBV and 11C-PMP, ligands for the vesicular acetylcholine transporter and acetylcholinesterase, respectively.^161,162^ Additionally, 11C-UCB-J, which binds presynaptic vesicle glycoprotein 2A on brain synapses, was recently investigated. There was significant and diffuse loss of synaptic density, including the brainstem in PSP-RS.^165^ This was evident in regions with and without significant atrophy, and global uptake negatively correlated with the PSPRS.^165^

### Single photon emission computed tomography

Although SPECT has revealed some perfusion changes supratentorially in PSP, there have been no studies showing significant perfusion abnormality in the brainstem.^174^ Few studies comparing PSP with healthy controls have demonstrated reduced dopamine transporter binding with 123Ibeta-CIT in the midbrain^177^ and pons.^176^ Utilizing brainstem and striatal dopamine transporter binding together may improve differentiation of PSP from Parkinson’s.^176^

### Cerebellar white matter and peduncles

#### Conventional and volumetric MRI

**Figure 4:**
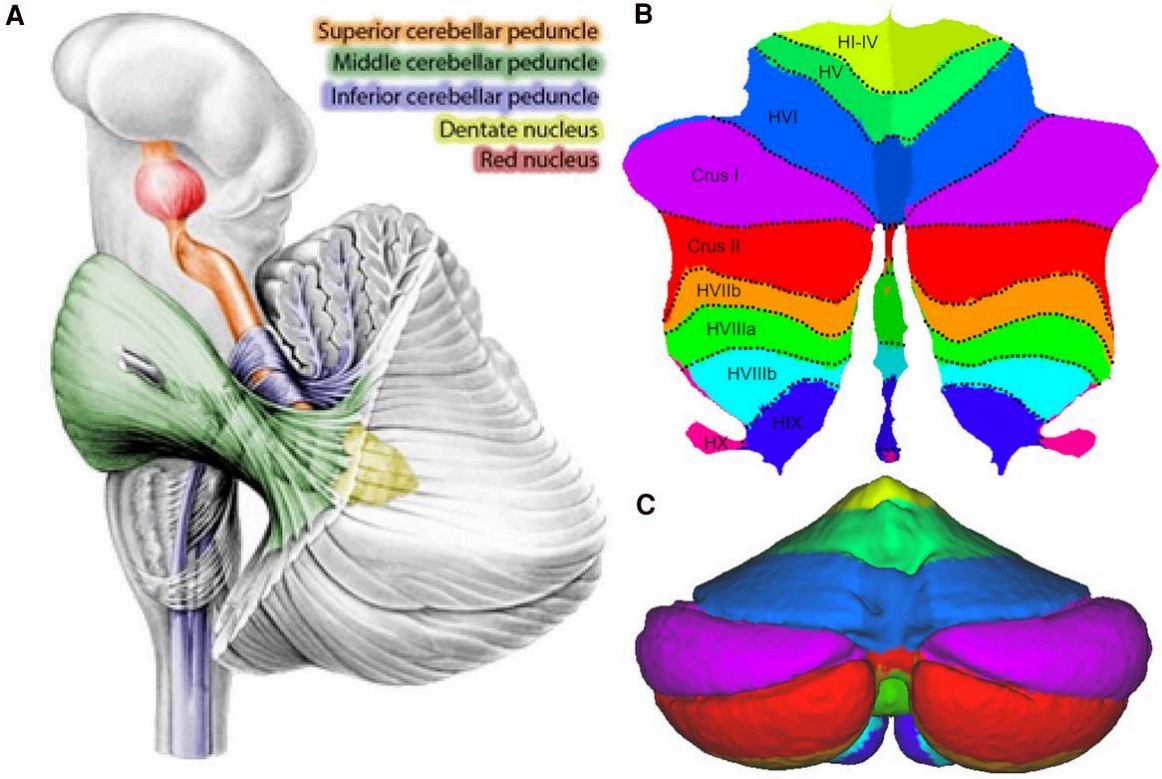
**The anatomical structure of the cerebellum and cerebellar peduncles.** (A) The cerebellar peduncles depicted on 3D brain templates, as defined using diffusion-weighted tractography. Reproduced with permission from van Baarsen *et al.*^196^ and Nieuwenhuys *et al*.^197^ (**B)** Flat map with anatomical subunits. Lobules within the cerebellar hemisphere are labelled HI-HX, and (**C**) *Posterior view with anatomical subunits.* Reproduced with permission from Diedrichsen *et al.*^198^

The cerebellar peduncles are pivotal structures connecting the cerebellum with the brainstem, cerebral cortex and spinal cord to facilitate broad network connections (Fig. 4). Abnormalities in the superior (SCP), middle (MCP) and inferior cerebellar peduncles (ICP) in PSP are summarized in Table 2. The SCP is the primary output tract of the cerebellum and arises largely from the efferent axons of the dentate nucleus. Volume loss in the SCPs has been reported in a large number of studies in PSP.^40,41,54,68,199,200,203,221^ In fact, SCP width is a key component of the magnetic resonance Parkinsonian index, which includes both midbrain-to-pons area ratio, and MCP-to-SCP width ratio.^222-224^ A few studies suggest that, similar to midbrain volume loss, the severity of SCP atrophy is greater in PSP-RS than other variants. Emphasizing this, significantly greater SCP atrophy has been reported in studies comparing PSP-RS with PSP-PAGF^203^ and PSP-P.^204,221^ There is also evidence that SCP volume significantly declines over time in PSP-RS.^225^ SCP degeneration may be directly related to pathology of the dentate nucleus (see below) and is in-line with reported microstructural abnormalities in the dentato-rubro-thalamic tract (DRTT)—of which the SCP is a key component—as reviewed above.

**Table 2:** Cerebellar white matter and cerebellar peduncle findings in the literature

		Cerebellar white matter	SCP	MCP	ICP
MRI					
Atrophy	+	^44-46,49,55,58,60,199-202^	^40,44,46,47,56,60,63,68,70,73,86,152,182,199,200,203-206^	^40,45-47,54,73,199,200,203^	^45,49,73^
	−	^1,38,40,48,61,63,65,70,74,85,181,205^	^31,85^	^41,48,49,86,205^	^40,41,46,48,199,200,205^
Microstructural damage	+	^46,49,50,61,95,100,102,184,200,207^	^33,46,49,50,61,71,91-96,100-102,105,109,183,184,200,201,204,207-217^	^36,46,61,91,101,106,200,202,214^	^36^
	−	^33,71,91,105,215,218^		^49,50,71,95,96,100,102,104,105,109,184,201,210-212,215,219^	^46,49,61,91,95,102,200,201,210,211,215^
PET					
↓metabolism	+		^108,135^		
	−	^24,25,108,110,111,132,133,136-143a^			
↑ tau	+	^149,156^			
	−	^33,48,147-150,218a^			
Neuroinflammation	−	^148^			
↓ BBB efflux	−	^166 a^			
SPECT					
↓perfusion	−	^220 a^			

^a^Studies using Statistical Parametric Mapping showing no significant findings in the cerebellar white matter and peduncles.

‘+’ rows list studies with significant findings compared with control or disease group ‘−’ rows list studies without significant findings compared with control or disease group. SCP = superior cerebellar peduncle; MCP = middle cerebellar peduncle; ICP = inferior cerebellar peduncle; BBB = blood brain barrier.

The MCP predominantly carries afferent fibres to the cerebellum, forming the second leg of the cortico-ponto-cerebellar tract. Reports of MCP atrophy in PSP are mixed. Several studies have demonstrated significant MCP atrophy,^49,54,199,200^ including a meta-analysis.^40^ In contrast, a similar number of studies did not report this finding.^41,48,49,86,205^ When seen, it appears to be relatively minor compared with SCP atrophy.^54,56^ The ICP, which predominately carries afferent fibres from the spinal cord, vestibular system and lower brainstem to the cerebellum, is spared in the vast majority of volumetric studies where it has been investigated,^40,41,46,48,199,200,205^ although there are a few reports of volume loss.^45,49^ The paucity of such reports is compatible with relative sparing of these peduncles, compared with the SCPs. Despite this, volume loss of all three sets of peduncles has been shown to occur over time in PSP-RS, alongside brainstem atrophy.^45^ No studies have shown MCP or ICP volume loss in specific PSP variant presentations.

When observed, cerebellar white matter atrophy appears alongside midbrain and SCP atrophy.^45,46,55,60,199^ It has been noted to worsen over time,^44,45^ and in one study, white matter volume loss was significantly associated with worsening cognition.^44^ Reports of cerebellar white matter atrophy have been most commonly reported in PSP-RS.^199,200^ Few studies have also reported significant findings in PSP-P and PSP-CBS compared with controls.^46,55,60^ Despite these findings, a voxel-based meta-analysis from 2019 reporting significant SCP atrophy did not find associated cerebellar white matter volume loss.^40^

#### Microstructural MRI

A range of studies, predominantly using DTI, have shown significant changes indicating microstructural damage in the SCPs.^33,46,50,91,184,204,207^ In multimodal studies, this is seen alongside significant SCP atrophy^33,46^ and has been shown to worsen over time.^50,94^ Moreover, free water and diffusion abnormalities in the SCP have been shown to correlate with the severity of oculomotor scores,^94^ PSPRS^201^ and cognition.^101,201^ As with other metrics, it appears these changes are more severe in PSP-RS when compared with other variants, including PSP-P or PSP-SL.^184,204^ Similar diagnostic accuracy to conventional MRI has been reported. Combining conventional MRI with DTI does not appear to significantly improve accuracy.^92^

While the majority of studies have not found significant changes in the MCPs,^49,50,71,95,96,100,102,104,105,109,184,201,210-212,215,219^ there are some reports of microstructural alterations.^46,61,91,101,214^ Interestingly, in one study, MCP changes were found to be significant in the PSP-RS group, but not the PSP-P group, compared with controls.^46^ It should be noted that this has not been a consistent finding,^61^ and distinctions between specific variants remain somewhat uncertain.

Similarly, most studies have not shown significant microstructural change in the ICPs.^46,49,61,91,95,102,200,201,210,211,215^ Only one study, using a region-of-interest DTI approach, showed significantly decreased fractional anisotropy in the ICP of PSP patients compared with controls.^36^ This was noted alongside significant DTI changes in the SCP and MCP, possibly suggesting disease spread.^36^ However, these findings did not significantly correlate with disease severity or duration.^36^

Whilst many did not observe significant changes,^33,71,91,92,94,105,182,215,218^ reductions in fractional anisotropy and increases in mean diffusivity have been reported in the cerebellar white matter as well.^46,50,95,184,200,207^ Importantly, the cerebellum is involved in the DRTT, projecting via the brainstem and SCP to the thalamus, with subsequent influence over cortical regions. Impairment of this key network has been demonstrated using DTI and tractography in PSP-RS, PSP-SL and PSP-P to differing extents.^46,70,93,183^ These findings are particularly notable in PSP-RS, and in one study the mean diffusivity in the DRTT correlated with disease duration.^46^ As with the brainstem findings, multimodal studies have shown that significant microstructural change in the cerebellum generally occurs alongside volumetric changes.^46,49,200^

#### Other MRI

As reported earlier, Ota *et al.*^189^ used DTI analysis along the perivascular space to assess the glymphatic system. The DTI analysis along the perivascular space index was reduced in the SCP, along with the brainstem, in PSP compared with controls.^189^ There were no significant changes in the MCP or ICP.^189^

#### Metabolic PET

Two studies of fluorodeoxyglucose-PET, using a voxel-wise approach, reported significant hypometabolism in the region of the SCP, alongside other regions including the midbrain.^108,135^ This has not been replicated in numerous voxel-based studies,^24,25,110,111,132,133,136-143^ and these data likely reflect the inherent metabolic differences between white matter and grey matter, whereby white matter tracts have lower metabolic activity.

### The cerebellum

#### Conventional and volumetric MRI

Neuroimaging changes in the cerebellum in PSP are summarized in Table 3. The cerebellum is a key infratentorial structure that can be subdivided into lobes and lobules which are variously implicated in a range of motor, cognitive, affective, language and social control functions (15). Atrophy of the cerebellar cortex has been reported in a range of studies using both region-of-interest volumetric methods or voxel-based morphometry,^45,46,52,69,76,199,202,218,226,227,231^ although this has not been universally replicated.^40,45,48,70,72,85,181^ Almost every region of the cerebellar cortex has been implicated, including the anterior lobe,^46,229^ posterior lobe,^46,76,203,231^ vermis^14,62,66^ and flocculonodular lobe.^76,231^ Reports of dentate nucleus atrophy are also available,^41,46,47,228^ which are compatible with post-mortem studies.^7,235^

**Table 3:** Cerebellar findings in the literature

		Hemispheric grey matter				Vermis	Flocculonodular lobe
		Region unspecified	Dentate nucleus	Anterior lobe	Posterior lobe		
MRI							
Atrophy	+	^37,52,60,69,71,218,226,227^	^41,46,47,202,228^	^14,36,46,73,229,230^	^14,40,46,47,73,76,203,229,231^	^62,66^	^76,231^
	−	^38,70,74,85,207^	^33,39,43,45,48,49,58,61,65,72,181,200,205a^				
Microstructural damage	+		^46,100^	^100-102^	^46,101,102^	^87,100-102^	
	−	^94^	^92^				
Iron deposition	+		^114,115,120^				
	−		^121,124^				
PET							
↑ metabolism	+	^110,133,136,193^					
↓ metabolism	+	^112,131^					
↑ or ↓ metabolism	−	^25,108,132,135,140,141b^					
↑ tau	+	^48^	^33,89,141,143-145,147-151,181,219^				
	−	^33,69,147,149,150^	^69,146,164^				
↓ Acetylcholine	+	^162^			^161^		
↓ synaptic density	+	^165^					
Neuroinflammation	+	^163^					
	−	^160^					
↓ BBB efflux	−	^166 b^					
MR spectroscopy							
Neuronal damage	+	^232^					
SPECT							
↓ perfusion	−	^174,175,233,234^	^220 b^				

^a^Voxel based studies, showing no significant volume loss in the cerebellum.

^b^Studies using Statistical Parametric Mapping showing no significant findings in the hemispheric grey matter (excluding the dentate nucleus).

‘+’ rows list studies with significant findings compared with control or disease group ‘−’ rows list studies without significant findings compared with control or disease group. BBB = blood brain barrier.

The spatial pattern of cerebellar grey matter atrophy is heterogeneous across studies, but two voxel-based meta-analyses of predominantly PSP-RS have provided insights into the most consistent findings.^14,230^ Pan *et al.*^230^ reviewed 18 whole-brain studies and found significant atrophy in the anterior lobe, including lobules III, IV and V. Gellersen *et al.*^14^ synthesized nine studies, and reported significant volume loss in the anterior lobe (lobules I–IV), posterior lobe (left crus I, crus II, lobule VIIb; right lobule IX) and vermis (left culmen). These authors propose that these changes correspond to both motor and non-motor manifestations of PSP,^14^ but only a few studies have found significant clinical correlations with cerebellar grey matter atrophy. One study of 15 PSP patients found the frontal assessment battery and disease duration positively correlated with cerebellar grey matter volume loss.^229^ An association was also reported with the postural instability gait disturbance sub-score and phonological verbal fluency in this study.^229^ Another small study reported a relationship between language semantics and grey matter loss in the vermis (lobules I–IV).^231^ There have been a few other reports showing associations between cerebellar grey matter loss and stance time variability,^226^ letter fluency^47^ and the PSPRS.^218^ However, others have failed to find significant cognitive^36,218^ or motor^36,47^ correlations with cerebellar structure in their analyses. A small number of studies have also reported cerebellar grey matter loss in variant PSP, including PSP-P,^46^ and PSP-CBS,^60^; however, such findings were not replicated in a larger volumetric study of variant PSP in 2020.^48^

Interestingly, two studies of reasonably accurate support vector machine classifiers included cerebellar voxels in their models for distinguishing PSP from Parkinson’s disease.^73,236^ It has been posited that this may primarily correspond to changes in Parkinson’s disease^42^; however, it is feasible that PSP-related alterations also contribute.

#### Microstructural MRI

As noted above there are mixed reports of cerebellar white matter tract damage,^46,50,95,184,200,207^ which is compatible with spread and dysfunction along the DRTT. There are a few reports of microstructural changes in the dentate nucleus^32,46^ and cerebellar cortex^46,102^ as well.

#### Other MRI measures

A few studies have investigated iron deposition in the dentate nucleus with variable results (Fig. 3). Some of these showed increased susceptibility or R2* values in PSP compared with control or Parkinson’s disease.^114,115,120^ In one study using quantitative susceptibility mapping, PSP could be distinguished from Parkinson’s disease using dentate nucleus susceptibility with an AUC of 0.826.^115^ Despite this, such findings have been inconsistent.^121,124^ Moreover, a study of 14 post-mortem brains using GRE T2* weighted imaging did not find a significant increase in the dentate nucleus.^237^ Whilst this may be due to the small sample size, or the effects of formalin, it raises further uncertainty.

#### Metabolic PET

Studies of fluorodeoxyglucose-PET have shown contradictory findings in the cerebellum. Several studies using a voxel-based SPM approach have reported cerebellar hypermetabolism in PSP compared with controls.^110,133,193^ Additionally, cerebellar regions were also included in the ‘PSP-related pattern’ of metabolic changes, derived from principal component analyses in each of these studies. These patterns reportedly showed high accuracy for distinguishing PSP from control or Parkinson’s disease groups.^110,133,193^ One study noted this significant finding in their PSP-RS, PSP-P and PSP-PAGF groups.^110^ Despite this body of evidence, a few similar studies did not find significant cerebellar hypermetabolism.^25,132,140,141^ Moreover, some have even reported significant cerebellar hypometabolism,^112,131^ with one study reporting a greater frequency of hypometabolism in PSP-RS and PSP-PI variants specifically.^131^

#### Tau PET

**Figure 5:**
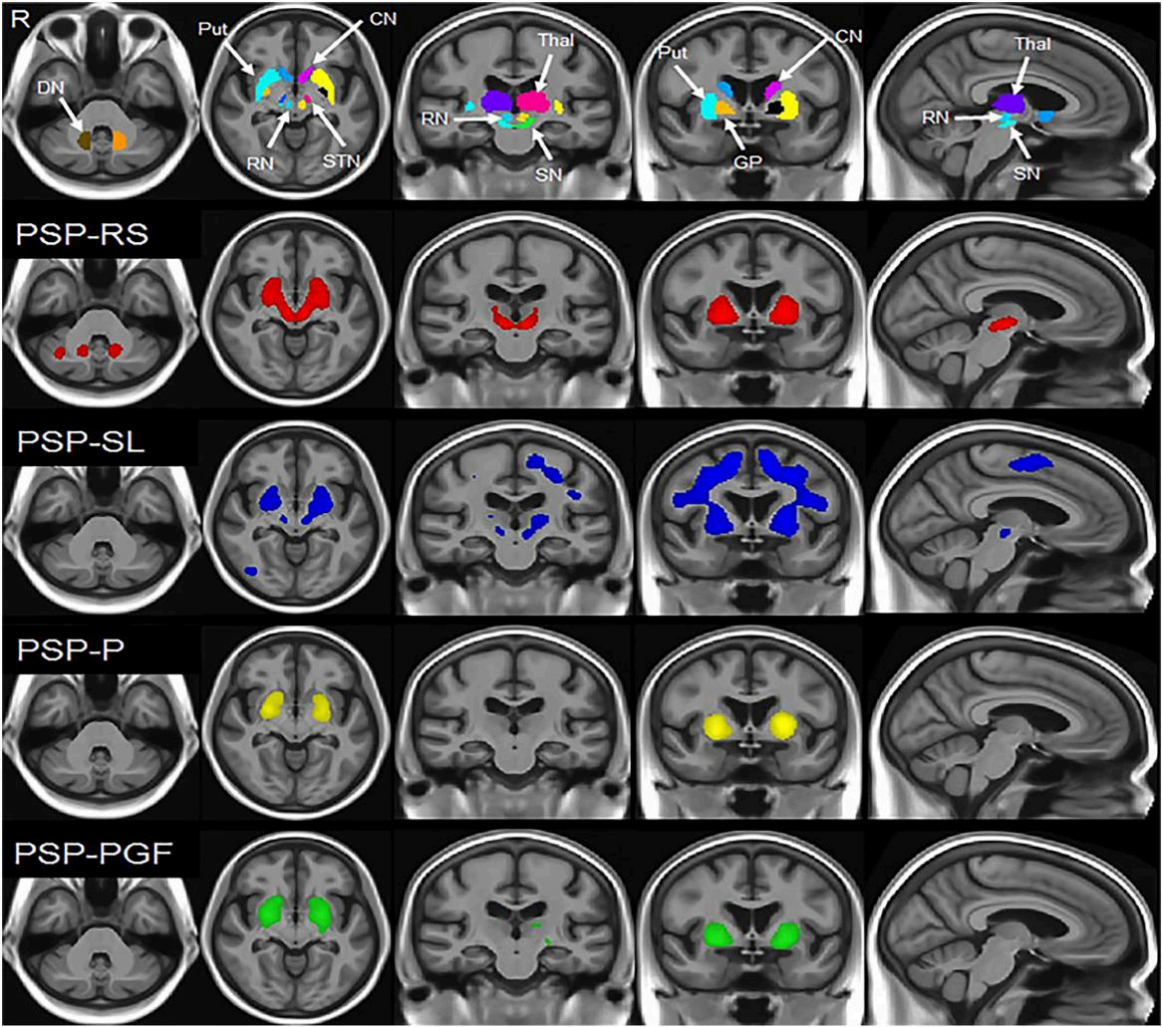
**PET imaging of flortaucipir uptake in PSP.** Regions of increased 18F-Flortaucipir uptake in PSP variants compared with controls. Figure reproduced with permission from Whitwell *et al*.^48^ PSP = progressive supranuclear palsy; PSP-RS = Richardson–Steel variant; PSP-F = frontal variant; PSP-CBS = corticobasal syndrome variant; PSP-SL = speech/language variant; PSP-P = Parkinsonism variant; PSP-PGF = progressive gait freezing variant; DN = dentate nucleus of the cerebellum; Put = putamen; CN = caudate nucleus; RN = red nucleus; STN = subthalamic nucleus; SN = substantia nigra; Thal = thalamus; GP = globus pallidus; R = right.

Studies of tau PET have frequently demonstrated an increased uptake in the dentate nucleus, compatible with post-mortem analyses.^48,147^ This has been shown in studies using a range of ligands including 18F-flortaucipir,^33,48,147,151,152,154,155,218^ 18F-PI-2620,^157^ 11C-PBB3^150^ and 18F-FDDNP.^156^ As noted previously, the majority have focused on 18F-flortaucipir, which has important interpretive limitations in PSP. Increased 18F-flortaucipir uptake has also been reported in the cerebellar cortex in PSP-RS and PSP-SL (Fig. 5)^48^; however, this has not been replicated in a number of other studies.^33,69,147,149,150,218^ One study reported a significant correlation between dentate nucleus 18F-flortaucipir uptake and cerebellar grey matter volume loss.^218^ Only a small number of studies have observed increased uptake in the cerebellar white matter,^147,218^ compared with multiple studies that have found no significant effects.^33,48,147-150,218^

Highlighting concordance with some volumetric findings, Sintini *et al.*^33^ showed that 18F-flortaucipir uptake in the dentate nucleus was associated with volume loss in the same region. A recent study also showed that dentate nucleus uptake was significantly greater in PSP than corticobasal degeneration; however, on partial least squares analysis, uptake in the red nucleus appeared to be a better discriminator of the disease.^147^ Over time, 18F-flortaucipir uptake also appears to increase in the dentate nucleus, along with the midbrain, pallidum and precentral cortex.^152^ Despite this, in their longitudinal study, Whitwell *et al.*^152^ found 18F-flortaucipir uptake did not show the same strong correlation with disease severity seen with midbrain atrophy.

#### Other PET ligands

Post-mortem studies have reported a significantly increased microglial burden—reflecting neuroinflammation—in PSP, which may follow the distribution of tau.^238^ The results of PET studies, however, have been conflicting. A small study in 2005 showed increased 11C(R)-PK11195 binding in the cerebellum,^163^ but this was not replicated in a larger study using a similar approach.^160^ Additionally, a study using 18F-THK5351, which has affinity for monoamine oxidase B, did not find significantly increased binding in the cerebellum.^148^

Lastly, studies of 18F-FEOBV and 11C-PMP PET also suggest a cholinergic deficit involving the cerebellum.^161,162^ This is compatible with post-mortem studies and is postulated to implicate the medial vestibular neurons which innervate the cerebellum, among other regions.^161^ This deficit was noted to correlate with the severity of gait dysfunction in PSP, as well as Parkinson’s disease and multisystem atrophy.^161^ In line with this, a study showing widespread synaptic loss using 11C-UCB-J PET, which is also seen in the cerebellum.^165^ Finally, despite a significant increase in 18F-altanserin binding in the brainstem, Stamelou *et al.*^159^ did not find serotonergic alterations within the cerebellum.

#### Single photon emission computed tomography

SPECT studies assessing cerebral perfusion have not demonstrated significant changes in the cerebellum when compared with controls or Parkinson’s disease.^174,175,220,233,234^ Few studies have looked at variant PSP. One study from 2022 reported significant cerebellar hypoperfusion in multisystem atrophy-P compared with PSP-P, which is consistent with the disproportionate cerebellar involvement in multisystem atrophy.^239^ As noted earlier, the paucity of perfusion changes infratentorially in PSP is at odds with significant changes reported supratentorially.^220^ It has been suggested that comparable cerebellar compensatory mechanisms in PSP and Parkinson’s disease may underlie this finding.^233^

## Discussion

An increasingly mature body of neuroimaging studies has provided in-depth characterization of the structural and functional changes in PSP using a range of methods. Numerous infratentorial alterations have been reported, implicating the brainstem and cerebellum in this disease. Well-established metrics, such as measures of midbrain atrophy or hypoperfusion, are increasingly being complemented by more novel methodologies and biologically diverse outcomes.

Midbrain atrophy and hypometabolism have been reported across many studies, and these findings are used to support a clinical PSP diagnosis.^4^ Additionally, there are a number of studies demonstrating microstructural damage in the midbrain, with some showing changes in the substantia nigra or red nucleus.^48,100,101^ Multimodal studies have demonstrated a concordance between diffusion changes and midbrain volume loss,^46,50^ emphasizing the presence of microstructural damage in association with atrophy. Such microstructural change is thought to precede atrophy; however, it remains to be seen whether these methods are useful in earlier disease detection. Some longitudinal studies demonstrate worsening of these changes over time.

Although pathological studies have suggested progressive rostral and caudal spread of tau from its core regions,^7^*in vivo* imaging studies implicating other brainstem regions are conflicting. Some studies have noted significant volumetric and microstructural damage in the pons or medulla, and one longitudinal study found that disease severity significantly correlated with pontine, as well as midbrain atrophy.^45^ Though this is compelling, several studies did not find significant involvement of other brainstem regions. The mixed results likely reflect variable study designs, small sample sizes and heterogeneity, particularly in disease duration or severity.

Along with the midbrain, SCP damage has been reported in many studies, in the form of atrophy or microstructural change.^46,200,204^ These important white matter tracts, joining the cerebellum and midbrain, are thought to be disproportionately affected in PSP, along with other components of the DRTT.^46,70,93,183^ Reports of MCP or ICP involvement are less robust. There are mixed reports of MCP abnormalities in PSP, and there is very little evidence implicating the ICPs. It is feasible that, with disease progression and tau spread, the MCPs and ICPs may become affected; however, they appear to remain relatively spared even when impairment is evident.^54,56^ This relative sparing, along with the aforementioned limitations, likely contribute to the inconsistent findings.

Imaging studies implicating the cerebellum in PSP are variable. Whilst numerous studies suggest volume loss and microstructural damage affecting various cerebellar regions,^37,46,55,201^ many do not.^43,45,48,61^ Some have reported clinico-radiologic correlations between cerebellar volume loss and domains including cognition, gait dysfunction, verbal fluency, language, and disease severity. Nevertheless, these reports are inconsistent, and no distinct pattern of cerebellar involvement is recognized in PSP. Moreover, functional imaging studies have shown mixed findings, with some reporting no metabolic change,^25,132,135^ and others reporting hypo-^112,131^ or hypermetabolism.^110,133,136^ A compensatory increase in cerebellar function has been proposed to underlie this.

The use of molecular imaging has been rapidly evolving, with multiple tau tracers discussed in the literature. This an exciting development as the ability to detect tau using *in vivo* imaging theoretically conveys the potential for earlier detection of disease. There are multiple reports of increased tau in the midbrain^33,48,69^ and dentate nucleus^33,48,147^ using these methods. Additionally, this appears to coincide with regional volume loss.^33^ Whilst this is promising, concerns of off-target binding are not insignificant, and this approach requires further refinement.

There is a smaller body of research exploring the use of newer imaging methods in PSP. These include MRI studies of iron deposition, neuromelanin loss, viscoelasticity, the glymphatic system, as well as molecular studies of specific neurotransmitters, markers of inflammation or synaptic density. The brainstem and cerebellum are implicated in a number of these, highlighting a spectrum of regional neurodegenerative alterations, and potential biomarkers for future research. Due to the small number of studies and novel approaches, further investigation is needed to clarify their validity and applicability.

It is important to emphasize that the aim of this review was to summarize the infratentorial imaging findings in PSP. This was not a rigorous appraisal of biomarker quality, which would require a comprehensive review of imaging studies across the whole brain, with a particular emphasis on longitudinal outcomes. There is a desperate need for well-powered longitudinal imaging studies, ideally including multiple imaging modalities, to directly establish the relative sensitivity of individual or composite imaging metrics outside of the midbrain to disease progression or subtyping. Moreover, confirmation of imaging outcomes in definite, post-mortem PSP cases is necessary, as most current studies include clinically possible or probable PSP. Lastly, despite increasing recognition of variant presentations, the literature is heavily focused on PSP-RS. Though some differences have been reported in the results, this review was focused primarily on providing an overview of salient findings in the literature. This is not a complete report of findings across variants. The radiological features discussed mainly apply to PSP-RS, unless otherwise specified.

## Conclusion

The brainstem and cerebellum are critical structures affected in PSP. Midbrain atrophy is an established supportive finding, and significant infratentorial findings beyond midbrain atrophy are also reported in the literature. Multiple structural and functional approaches have been investigated and show great potential in improving our understanding and diagnosis of this disease.

## Supplementary Material

fcaf051_Supplementary_Data

## Data Availability

Data sharing is not applicable to this article as no new data were created or analysed in this study.
