# Supplementary material for: Brainstem and cerebellar radiological findings in progressive supranuclear palsy
Source: Brain Commun. 2025 Feb 5;7(1):fcaf051. doi: 10.1093/braincomms/fcaf051 (PMC11829206; doi:10.1093/braincomms/fcaf051)
Supplement: fcaf051_Supplementary_Data [file fcaf051_supplementary_data.docx]

**Supplementary table 1:** Main demographics and findings of studies reviewed

| Study | Patient population | Modality | Measures | Disease duration (mean or median) | Imaging findings and correlations |
| --- | --- | --- | --- | --- | --- |
| Chatterjee et al 2023^1^ | 18 PSP-RS  13 VaP | 3T MRI | Volumetry (freesurfer) | 2.94+/-1.86 | PSP-RS vs VaP  Bilateral cerebellar cortex volume lower  Stance time variability correlates with R cerebellar cortex volume |
| Ota et al 2023^2^ | 24 PSP (10 RS, 4 P, 6 PAGF, 3 C, 1 CBS)  42 HC | 3T MRI | DTI along the perivascular space (DTI-ALPS) index  Automated volumetric measures | 7+/-4 | PSP vs HC  Reduced DTI-ALPS  Correlated with midbrain and pons volumes |
| Nobileau et al 2023^3^ | 19 PSP  98 HC  47 iRBD  75 PD+RBD  142 PD-RBD  19 MSA (12 with proven RBD) | 3T MRI | Neuromelanin MRI automated software looking at locus coeruleus/subcoeruleus complex (avg L+R) | 4.9+/-3.1 | HC vs PSP  Significantly higher signal intensity  No significant difference comparing PSP with PS+RBD, or MSA |
| Oliveira Hauer et al 2023^4^ | 23 PSP (19 RS, 6 P, 4 F, 2 PGF, 1 SL)  47 SYN  61 HC | PET  MRI | [18F]RO948  *Tau tracer*  ROI include SN | 3.55+/-2.0 | PSP vs HC  Significantly lower M/P  Lower tau PET uptake in substantia nigra  No significant correlations between these measures and NfL/other cognitive measures (but PSP group noted to have higher NfL) |
| Matsuoka et al 2023^5^ | 20 PSP  23 HC | PET  3T MRI  MR spectroscopy | 18F-PM-PBB3  Volumetry  Whole brain voxel based analysis of PET (using SPM)  Glutathione levels (to assess oxidative stress) - using VOI in ACC/PCC only | 3.5 +/- 1.7 | PSP vs HC  Increased SUVR in midbrain (also putamen, pallidum, motor cortex)  Also reduced volume in midbrain and exterior cerebellum (no MNI)  No significant difference in glutathione levels  No correlations with infratentorial regions |
| Welter et al 2023^6^ | 15 PSP-RS  16 Carribean AP  17 HC | 3T MRI | VBM  Midbrain metrics | 5 (1-8) | PSP-RS vs HC  Midbrain lower  White matter volume loss in mesencephalon  Superior and middle cerebellar peduncle diameters lower  White matter volume loss in bilateral cerebellar hemispheres  PSP-RS vs Carribean AP  Midbrain diameters lower  Superior and middle cerebellar peduncle diameters lower  Lower grey and white matter volumes in mesencephalon, cerebellar hemispheres  In both groups - severity of gait and balance disorder correlated with lateral cerebellum (among other regions) grey matter volume  In PSP-RS group - severity of gait and balance disorder correlated with cerebellar, midbrain white matter volume  Also total recall / OD-80 scores correlated with midbrain grey matter volume (among other regions) |
| Painous et al 2023^7^ | 21 PSP  14 CBD  26 MSA  12 PD  11 HC | 3T MRI | Automated volumetry (Freesurfer)  Shape MRI  Pons and midbrain | 5.4 (3.1-7.3) | PSP vs MSA  Upper posterior midbrain and small areas of rostral pons atrophy  Greater brainstem atrophy correlated with PSPRS  Negative correlation between brainstem atrophy and SEADL score  Midbrain/pons atrophy correlated with CSF NfL |
| Chougar et al 2023^8^ | 19 PSP  25 PD  27 MSA  22 HC | 3T MRI | DTI  Midbrain metrics  ROI including red nucleus | 4.7+/-3.2 | PSP vs HC/ PD  Higher MD and R2* in red nucleus  Similar accuracy with MD in red nucleus (AUC 0.871) compared with MRPI / M/P (AUC 0.93 / 0.912)  Slightly better using posterior putamen (0.911) |
| Gatto et al 2022^9^ | Definite cases  12 PSP-RS  10 PSP-SL  11 AD-LPA  10 HC | 3T MRI | Whole brain DTI tractography  Specifically tractography of SCP / DRTT  Connectivity using ROI of GM along DRTT | unspec | PSP-RS vs HC / AD-LPA  Increased MD and decreased FA in SCP  Lower FA in DRTT  PSP-SL vs HC / AD-LPA  Decreased FA in SCP  Lower FA in DRTT  PSP-RS vs PSP-SL  No difference in SCP FA  Increased MD in SCP  Lower FA in DRTT  No difference in DRTT MD  Correlation between SCP FA and saccadic impairment PSP-RS>PSP-SL, but stronger association between DRTT FA and saccadic impairment in PSP-SL  Correlation between PSPRS/UPDRS III and SCP/DRTT DA in PSP-SL but not PSP-RS  Correlations did not survive corrections |
| Tomse et al 2022^10^ | 20 MSA-P  20 PSP  20 HC  Validation group  56 MSA  45 PSP  116 PD  61 HC | FDG PET  CT | Principal component analysis to identify specific MSA and PSP patterns | 5+/- 1.6 | PSP vs HC  PSP related pattern included decreased metabolism in mesencephalon, and relative hyper-metabolism in the cerebellum  No correlation between PSP related pattern expression score and disease duration |
| Kanel et al 2022^11^ | 8 PSP  107 PD  19 HC | PET  3T MRI | [ 18F]-FEOBV  *Vesicular acetylcholine transporter* | 3-16 years | PSP vs PD  Lower binding in superior colliculus, inferior colliculus, inferior olivary nuclei, cerebellum (bilat crus I, II and lobules IV, V, VI, VIIB, VIII, IX  Post-hoc VOI-based comparison shows lower binding in midbrain, pons, medulla  PSP vs HC  Lower binding in inferior olivary nuclei, dorsal medulla, left>right crus I and crus VIIB |
| Whitwell et al 2022^12^ | 29 PSP-RS, 12 PSP-P  9 PSP-SL  22 HC | 3T MRI | DTI | RS 5(3.2-5.8)  P  6.5(5-8.1)  SL  7(4.9-8.3) | PSP-RS vs HC  Increased MD/reduced FA in cerebellum on voxel-level analysis  PSP-RS vs HC / PSP-SL  Reduced FA in SCP  PSP-RS vs P/SL  Increased MD in SCP  PSP-P/SL vs HC No significant brainstem/cerebellar changes in FA  PSP-RS/P/SL vs HC  Increased MD in SCP |
| Tessema et al 2022^13^ | 11 PSP  51 PD  31 HC | 3T MRI | SWI  R2*  Automated volumetric measurement of iron-rich regions in substantia nigra using deep learning | 4.4+/-2.1 | PSP vs HC / PD  Reduced substantia nigra volume and surface area (which were highly associated with each other)  Mean curvature different  R2* vol to distinguish PSP  AUC 0.96 vs HC  AUC 0.98 vs PD |
| Buciuc et al 2022^14^ | *Definite*  30 PSP  20 CBD  7 GGT | 3T MRI | VBM | unspec | PSP vs GGT  Midbrain and medulla white matter atrophy  Cerebellar grey matter atrophy |
| Hosapatna et al 2022^15^ | 15 PSP  35 PD  50 HC | 1.5T MRI | Substantia nigra pars compacta width | unspec | PSP vs PD / HC  Reduced mean pars compacta thickness |
| Seniaray et al 2022 | 88 PSP (46 RS, 7 PGF, 9 PI, 3 SL, 6 F, 11 P, 6 CBS)  15 HC | FDG PET | SPM | Mean 1.25 years  (1.4-3) | Overall PSP vs HC  Significant hypometabolism in brainstem/mesencephalon (among other regions)  67% had hypometabolic clusters in brainstem (highest in SL 100% and lowest in PI 44.4%)  28% had hypometabolic clusters in cerebellum (highest in PGF 42.8% and lowest F 0%) |
| Mazzucchi et al 2022^16^ | 24 PSP  5 MSA-P  9 MSA-C  31 PD | 3T MRI | Morphometric indices such as MRPI  QSM  ROI | 3.5 (3.0-5.0) | PSP vs PD / MSA-P  Significant differences in P/M and MRPI (AUC 0.976)  SNImed did not differentiate  PSP vs PD  Increased susceptibility in red nucleus and all regions of substantia nigra  AUC for RN 0.942  AUC for SN (medial part) 0.854 |
| Chougar et al 2022^17^ | 22 PSP-RS  38 PD  22 HC  20 MSA  7 DLB  4 CBS | 3T MRI | Neuromelanin-sensitive MRI SNc | 4.7 +/-3.2 | PSP vs HC / PD / MSA  Lower SNR in SNc and lower volume  SNR reduced in associative region in particular (no significant difference in limbic or sensorimotor territories) |
| Wang et al 2022^18^ | 39 PSP-RS  73 HC  31 CBS  47 bvFTD  36 nfvPPA  50 svPPA | 3T MRI | Perivascular spaces,  white matter hyperintensities | unspec | PSP-RS vs HC  Larger brainstem perivascular spaces |
| Zhang et al 2022^19^ | 18 PSP  28 MSA  47 PD  28 HC | 3T MRI | SWI  QSM | 2+/-3.25 | PSP vs HC / PD  Higher susceptibility values in substantia nigra, red nucleus, dentate nucleus  PSP vs HC  Substantia nigra AUC 0.853  Red nucleus AUC 0.855  Red nucleus + NfL 0.994  PSP vs PD  Substantia nigra AUC 0.764  Red nucleus AUC 0.829  Dentate nucleus AUC 0.826 |
| Alster et al 2022^20^ | 16 PSP-P  20 MSA-P | SPECT | 99mTc-HMPAO  ROI | 3-6 years | PSP-P vs MSA-P  No significant difference in the cerebellum |
| Ye et al 2021^21^ | 14 PSP-RS  25 PD  24 HC | 7T MRI | ultra high field 7T MRI - neuromelanin sensitive  Atlas based  Voxelwise analyses | 4.24+/-2.68 | PSP vs HC  Caudal LC signal change  PSP vs PD  No difference  Apathy score negatively correlated with LC contrast to noise ratio  Bilateral LC clusters in voxelwise analysis correlated with MoCA scores |
| Zhao et al 2021^22^ | *Pre-training*  28 PSP  342 PD  45 MSA  43 HC  *Training*  54 PSP  146 PD  79 MSA | PET  MRI | DAT [11C]CFT PET  SPM, saliency maps  Also comparison of deep-learning radiomics vs conventional | 32.4±21.4 months | PSP vs HC  Saliency map includes significant contribution of midbrain uptake *exact regions not listed  Good accuracy of deep learning method (including a range of regions) |
| Aghakhanyan et al 2021^23^ | 14 PSP-RS  10 vPSP  10 MCI | MRI  PET | Resting state fMRI  [18F]PI-2620  *Tau tracer* | unspec | PSP vs MCI  Cerebellum showed hyperconnectivity with cortical associative nodes  (ie. likely adaptive changes in cortico-cerebellar networks)  Cerebellum showed lower connectivity with globus pallidus, brainstem, limbic cortex (ie, mainly dysfunction in the subcortical-cerebellar networks)  PSP-RS vs vPSP  No significant differences in uptake  Increased uptake in red nucleus and cerebellar dentate nucleus in PSP (based on Z scores) |
| Choi et al 2021^24^ | 66 PSP-RS  28 PSP-P  29 PSP-PGF  80 PD | 3T MRI | Volumetric measurement of brainstem and superior cerebellar peduncle | 3.0 (2.2) 5.0 (3.1) 4.2 (2.5)  Overall 3.7 (2.6) | PSP RS / P / PGF vs PD  Midbrain atrophy  No significant difference for SCP  PSP-P / PGF vs PD  Pons and midbrain smaller vol  No significant difference between PSP-RS vs PSP-P vs PSP-PGF  SCP volume correlated with vertical saccadic peak velocity and saccadic accuracy |
| Woo et al 2021^25^ | 12 PSP  22 HC | 3T MRI | Volumetry  Freesurfer | 2.2 +/-1.3 | PSP vs HC  Lower midbrain volume and M/P  No difference in medulla, pons, superior cerebellar peduncle or cerebellum  No significant correlation with peripapillary retinal nerve fibre layer thickness on OCT |
| Lee et al 2021^26^ | 5 PSP  5 PD  5 HC  Also post-mortem histopathological exam | 3T MRI | R2* and QSM (quantitative susceptibility mapping**)** between substantia nigra and red nucleus | 3.4 +/-0.5 years | PSP vs HC / PSP vs PD  Significantly higher R2* and QSM suggesting iron deposition along the myelinated fibres (in the anterior substantia nigra/oculomotor nerve fascicles) |
| Buch et al 2021^27^ | 14 PSP  15 HC | MRI | Manual volumetric measurement of brainstem structures | unspec | PSP vs HC  Midbrain, tegmentum, tectum, medulla all significantly reduced volume  No difference in pons  Midbrain atrophy significantly correlated with PSPRS  Extent of limited upward gaze correlated with midbrain atrophy  Trended for down-gaze (p=0.08) |
| Matsuura et al 2021^28^ | 49 PSP  244 PD  19 MSA-P  11 disease control | 3T MRI | Neuromelanin-sensitive MRI  CNR in LC  Area of SN | 3.69+/-1.89 | PSP vs disease controls  Smaller SNc  Lower LC contrast signal  PSP vs PD / MSA-P  Higher LC contrast signal |
| Li et al 2021^29^ | 20 PSP  7 MSA-P  10 PD  13 HC | PET  MRI | 18F-APN-1607  *Tau tracer*  ROI  SUVR  Cerebellar GM reference | 45.1+/-30.0 months | PSP vs HC / PD / MSA-P  Increased uptake in midbrain, tegmentum, substantia nigra, red nucleus, pontine base, raphe nuclei, locus coeruleus  No significant difference in dentate nucleus |
| Marti-Andres et al 2020^30^ | 47 PSP-RS  18 PSP-P  8 PSP-PAGF  55 HC  58 PD | FDG PET | SPM  PCA/SSM  PSP-related pattern, cross validated | 2.5 (1.6-3.6) | PSP vs HC  Hypometabolism in midbrain, among other regions  Hypermetabolism in cerebellum  PSP vs PD  Hypometabolism in midbrain, among other regions  PSP-RS vs HC  Hypometabolism in midbrain  Hypermetabolism in cerebellum  PSP-P vs HC  Hypometabolism in midbrain  Hypermetabolism in cerebellum  PSP-PAGF vs HC  Hypermetabolism in cerebellum |
| Whitwell et al 2020^31^ | 53 PSP-RS  23 PSP-SL  12 PSP-P  8 PSP-CBS  5 PSP-F  4 PSP-PGF  30 HC  *21 of these autopsy confirmed* | 3T MRI  PET | Volumetry  Flortaucipir ([18F]AV-145)  voxel/region level analyses | RS 3.4 (2-4)  P 6.6 (4.8-8.5)  PGF 2.5 (2-3)  SL 6.3 (4.2-7.6)  3.8 (3-4)  F 3.4 (2-4) | Volumetry  PSP-RS / PSP-F / PSP-CBS vs HC  White matter loss in superior cerebellar peduncle  White matter loss in midbrain  PSP-P / PSP-PGF vs HC  White matter loss in midbrain  PSP-RS vs PSP-SL / PSP-P  Smaller midbrain, substantia nigra, red nucleus volume  PSP-CBS / PSP-F vs PSP-SL  Smaller midbrain, substantia nigra, red nucleus volume  PSP-F vs PSP-P  Greater white matter loss in superior cerebellar peduncle  No differences in cerebellar vol across variants  PET  PSP-RS vs HC  Increased uptake in dentate nucleus, cerebellar grey matter  Increased uptake in red nucleus, midbrain  PSP-SL vs HC  Increased uptake in cerebellar grey matter  Increased uptake in midbrain  Did not correlate findings with pathology |
| Holland et al 2020^32^ | 14 PSP-RS  15 CBS  15 HC | PET | [11C]UCB-J  *Binds synaptic vescicle Glycoprotein 2A - marker of synaptic density* | 4.28 +/- 2.57 | PSP vs HC  Reduced binding in midbrain  Reduced binding in cerebellum  PSP vs CBS  No significant difference  Positive correlation between global binding and ACE-R  Negative correlation between global binding and PSP-RS |
| Palmisano et al 2020^33^ | 14 PSP  14 HC | PET | [18F]FDG  SPM | 5.3+/-3.1 | PSP vs HC  Hypometabolic region in midbrain in PSP  Not in cerebellum  TOCoPCoM (CoP-CoM distance at stance foot toe-off) correlated with midbrain uptake  CoP = center of pressure, CoM = center of mass |
| Tse et al 2020^34^ | 16 PSP  33 CBS  33 HC | 3T MRI | VBM of cerebellum | 44.3 +/- 37.4 months | PSP vs HC  Lower grey matter density in posterior lobe (Crus I-II, lobules VIIb and VIIa bilaterally, and smaller clusters in left lobules VIIIb, IX) and flocculonodular lobe (X)  PSP vs CBS - no differences  Language semantics correlate with atrophy in vermis of lobule I-IV |
| Murakami et al 2020^35^ | 8 PSP  16 MSA  24 PD  27 HC | SPECT | *123I -IMP-SPECT*  ROI - bilateral whole cerebellum | 29.4 +/- 31.5 months | PSP vs HC / PD / MSA  No significant difference in cerebellar perfusion |
| Ghirelli et al 2020^36^ | *Definite*  10 PSP  10 CBD  3 FTLD-TDP  1 Pick’s disease | PET  3T MRI  Cerebellar crus standardised | *Tau tracer*  18F-flortaucipir  (formerly 18F-AV-1451/18F-T807)  *Amyloid beta tracer*  Pittsburgh compound B | Age at onset 71 (54-74)  Age at PET 76 (61-83) | PSP vs HC / CBD / FTLD-TDP  Increased flortaucipir uptake in midbrain  Increased flortaucipir uptake in left dentate nucleus  PSP vs CBD  Higher uptake in red nucleus (AUC 0.93)  Higher uptake in cerebellar dentate (AUC 0.88) |
| Anagnostou et al 2020^37^ | 12 PSP  12 PD  12 HC | 3T MRI | VBM | unspec | PSP vs PD  Lower midbrain voume  Smaller cerebellar lobules VI (posterior lobe) and X (flocculonodular lobe)  No correlation of brainstem/cerebellar atrophy with square wave jerk parameters |
| Brendel et al 2020^38^ | 40 PSP-RS  20 vPSP  10 SYN  10 AD  10 HC | PET | *Tau tracer*  18F-PI-2620  ROI | PSP-RS 49+/- 38mths  vPSP 42+/- 37 | PSP-RS vs HC  Greater uptake in dentate nucleus (also compared with AD)  Greater uptake in substantia nigra (likely off-target neuromelanin binding)  No significant difference in dorsal midbrain  vPSP vs HC  No significant binding differences in brainstem/cerebellum  PSP-RS or vPSP vs SYN  No differences in brainstem/cerebellum  No significant correlation with PSPRS / disease duration |
| Schroter et al 2020^39^ | 7 PSP  2 AD  2 CBS | PET | FDG  11C-pyridinyl-butadienyl-benzothiazole  3 (11C-PBB3)  *Tau tracer* | 29 months (7-47) | PSP vs AD  Mean 11C-PBB3 SUVR in 18F-FDGPET-derived hypometabolic clusters showed a significant difference in the midbrain  Not significant 11C-PBB3 SUVR when derived from volumes of interest  PSP vs CBS  No significant difference in uptake in brainstem |
| Potrusil et al 2020^40^ | 15 PSP-RS  13 PSP-P  18 PD  20 HC | 3T MRI | DTI with probabilistic tractography  DRTT in addition to other tracts  Volumetry - automated segmentation including midbrain - doesn’t include MCP/ICP | 2.7+/- 1.8 | PSP-RS / PSP-P vs HC / PD  Lower volume in midbrain and SCP  Decreased FA and increased MD in DRTT  PSP-RS vs PSP-P  Lower SCP volume  Decreased FA and increased MD in DRTT  No significant volume loss in pons, medulla, cerebellar GM/WM |
| Abos et al 2019^41^ | 19 PSP  20 HC | 3T MRI | DWI  Structural connectivity derived from probabilistic tractography using ROI (68 cortical regions + 18 deep GM regions - listed in supp table)  DGM regions include cerebellar WM/MCPs, SN/RN  Also volumetry (ROI includes cerebellum) | 4.16 +/-2.03 | PSP vs HC  Reduced FA in cerebellar peduncles (?MCPs only assessed)  Smaller bilateral cerebellum  Multiple connectivity changes between cortico-cortical, cortico-DGM (some include cerebellum) and DGM-DGM tracts (1x R hippocampus - R cerebellum)  Connectivity changes used to differentiate PSP from HC with 82.23% accuracy |
| Mazzucchi et al 2019^42^ | 15 PSP  14 MSA  36 PD | 3T MRI | QSM of specific regions | 3.8+/-1.6 | PSP vs PD  Higher susceptibility in medial, lateral and ventral substantia nigra  Higher susceptibility in red nucleus  AUC PD vs PSP  RN 0.929  SNImed 0.865  SNIlat 0.818  SNIIventr 0.75  PSP vs MSA  Higher susceptibility in medial substantia nigra and red nucleus  AUC MSA vs PSP  RN 0.826  SNImed 0.785  No significant correlations in PSP group |
| Whitwell et al 2019^43^ | 20 SL-progressors (meeting PSP-RS criteria at follow up)  23 SL-stable  (progressive apraxia of speech not meeting criteria at follow up)  20 HC | 3T MRI | VBM | Unspec at time of MRI | PSP-RS (SL-progressors) vs HC / SL-stable  Brainstem, SCP WM  and cerebellar dentate loss |
| Nicastro et al 2019^44^ | 23 PSP-RS  23 HC | 3T MRI  PET | Voxel-based / surface based morphometry  DTI  18F-AV1451  *Tau tracer - in high binding regions (thalamus, putamen, cerebellar dentate)* | 4.6±2.1 | PSP vs HC  Grey matter volume loss in midbrain  Grey matter volume loss in cerebellum  High binding in midbrain was related to right temporal grey matter loss  Higher dentate nucleus 18F-AV1451 binding correlated with lower grey matter volume in bilateral cerebellum, as well as volume loss/cortical  PSP-RS correlated with grey matter loss in left cerebellum  No correlation with 18F-AV1451 binding  ACE-R did not correlate |
| Sintini et al 2019^45^ | 34 PSP  29 HC | PET  3T MRI | Flortaucipir  Volumetry  DTI  voxel-wise | 3.3 (2-4.8) | PSP vs HC  Reduced GM vol in midbrain and WM vol in midbrain, substantia nigra, SCP  Elevated flortaucipir signal in cerebellar dentate, midbrain, red nucleus  Reduced FA and elevated MD in midbrain, SCP  associations between elevated flortaucipir uptake in the cerebellar dentate, red nucleus, and decreased volume in the same regions, and decreased fractional anisotropy and increased mean diffusivity in tracts including the superior cerebellar peduncle, sagittal striatum and posterior corona radiata |
| Ghourchian et al 2019^46^ | 17 PSP  18 PD | TCS | Midbrain area, diameter of third ventricle and substantia nigra echogenicity | 3.35+/-1.5 | PSP vs PD  Smaller midbrain area  Larger third ventricle diameter  Lower substantia nigra echogenicity  Though, low accuracy on ROC analysis |
| Ramanan et al 2019^47^ | 20 PSP  29 HC  29 AD  44 CBS | 3T MRI | VBM  DTI (white matter tracts not including brainstem or cerebellum) | unspec | PSP vs HC / AD / CBS  Grey matter atrophy in cerebellar cortices (supplemental) |
| Pyatigorskaya et al 2019^48^ | 11 PSP-RS  26 HC  51 PD | 3T + 7T MRI | Diffusion  Volumetry  ROI analysis  Grades require AUC >0.7 (PSP vs HC) and then calculated based on combination of changes | 3.8+/-1.5 | PSP vs HC  Grade 3 change in substantia nigra (SN), midbrain, locus coeruleus (LC), pedunculopontine nucleus  Grade 2 change in pons  Best predictors of PSP: SN vol, FA midbrain, vol midbrain, vol brainstem  Grade 2 change in dentate nucleus, cerebellar cortex  PSP vs PD  Best predictors of PSP: SN vol, pons FA, midbrain vol |
| Sjostrom et al 2019^49^ | 11 PSP  10 MSA  44 HC  134 PD | 3T MRI | Susceptibility  ROI including RN, SN, dentate nucleus | 5.5 +/- 2.8 | PSP vs HC / MSA / PD  Higher susceptibility in red nucleus  PSP vs PD  Higher susceptibility in substantia nigra  Higher susceptibility in dentate nucleus |
| Schonecker et al 2019^50^ | 13 PSP-RS  9 MSA-P  6 MSA-C  6 PD | PET  MRI | [18F]-THK5351  *Tau and MAO-B tracer (thought to be astrogliosis related MAO-B elevation)*  VOI in target regions known to be affected by MAO-B elevation and tau (includes midbrain and pons and cerebellar deep white matter)  SUVR | 33.7 ± 19.5 months | PSP vs PD / MSA-P / MSA-C  Higher mean SUVR in midbrain  No significant increase in pons or cerebellar white matter (MSA-C>PSP)  Significant correlation between H&Y and midbrain uptake |
| Mitchell et al 2019^51^ | 26 PSP  44 PD  21 MSA-P  24 HC | 3T MRI | Free water (FW) imaging  Neurite orientation dispersion and density imaging (NODDI) | 3 +/- 2.5 (since Dx) | PSP vs HC  Significant differences FW and NODDI measures in the ant/post substantia nigra, red nucleus, pedunclulopontine nucleus, (among other regions of interest)  Significant differences FW and NODDI measures in superior cerebellar peduncles, lobule V and VI (among other regions of interest)  Also dentate and vermis on analysis of FW imaging |
| Endo et al 2019^52^ | 13 PSP  18 HC | PET | [11C]pyridinyl-butadienyl-benzothiazole 3  *Tau fibril tracer*  ROI including midbrain, pons, medulla, cerebellar WM + cerebellar and midbrain GM + SN/RN/dentate | 4.9 +/- 2.9 | PSP vs HC  Increased uptake in red nucleus, midbrain WM, cerebellar dentate  No significant correlations with brainstem or cerebellar findings and PSPRS  Not pons, medulla, SN, cerebellar WM/GM |
| Spotorno et al 2019^53^ | Cohort 1:  16 PSP  34 DLB  44 HC  Cohort 2:  34 PSP  25 DLB  32 HC | 3T MRI | DTI  *Fractional anisotropy (FA) score (median values from white matter + superior cerebellar peduncle)* | Cohort 1:  6+/-2  Cohort 2:  3+/-1 | PSP vs DLB / HC  Significantly different FA score  High classification score using this in validation cohort (AUC 0.96) |
| Sjostrom et al 2019^54^ | 29 PSP  27 MSA  140 PD | 1.5 or 3T MRI | Automated volumetry of brainstem and superior cerebellar peduncles  (freesurfer)  Only includes SCPs, not MCP/ICP/cerebellum | 3.1+/-1.8 | PSP vs PD / MSA  Smaller midbrain  Smaller SCP  Smaller medulla  PSP vs PD  Smaller pons  Midbrain AUC 0.90 for PSP vs PD  Midbrain + medulla AUC 0.92 for PSP vs PD and 0.85 for PSP vs MSA |
| Nigro et al 2019^55^ | 31 PSP-P  36 PSP-RS  36 PD  37 HC | 3T MRI | Track density imaging | PSP-P  7.1 +/- 3.5  PSP-RS  3.3+/- 1.3 | PSP-P vs HC / PD  Decreased track density in brainstem, superior cerebellar peduncle  PSP-RS vs PD  Decreased track density in brainstem, superior cerebellar peduncle  PSP-P vs PSP-RS  Significantly higher values of track density in superior cerebellar peduncle  No correlation with MMSE/UPDRS/H&Y  Support vector machine analysis differentiated PSP-P from PSP-RS (AUC 0.76), PSP-RS from PD (0.96) using superior cerebellar peduncle voxels |
| Alonso-Canovas et al 2019^56^ | 98 PSP  40 MSA  14 CBD  254 PD  145 HC | Transcranial ultrasound | Substantia nigra hyperechogenicity  Third ventricle enlargement | 5.2+/-2.5 | PSP vs PD / MSA / HC  More likely to have enlarged 3rd ventricle  PD / CBD vs PSP  More likely to have hyperechogenic substantia nigra  PSP not compared with controls |
| Azuma et al 2019^57^ | 8 PSP  18 PD  18 HC | 3T MRI | Midbrain area  QSM  ROI involving the red nucleus and substantia nigra  Created decision tree | 40.5 ± 40.3 months | PSP vs HC  Higher mean susceptibility value in all regions of the substantia nigra and the red nucelus  PSP vs PD  Higher mean susceptibilityvalues in all regions of the substantia nigra |
| Alster et al 2019^58^ | 21 PSP  14 CBS  10 MSA-P | SPECT  MRI | 99mTc-HMPAO  *Perfusion*  ROI | Range 2-6 years | PSP vs CBS  No difference  PSP vs MSA  Only difference is reduced thalamic perfusion  MSA vs PSP  Reduced left cerebellar perfusion |
| Quattrone et al 2019^59^ | 48 PSP-RS  30 PSP-P  37 PD  38 HC | 3T MRI | VBM (Grey matter)  DTI (whole brain FA)  planimetry | PSP-RS 4+/-1.7  PSP-P  8.2+/-3.1 | VBM:  PSP-RS vs PSP-P  No difference  PSP (O1) vs PSP (O2)  No difference  PSP (O2) vs PD  Significant GM volume loss in regions including midbrain and cerebellar cortex (not all MNI specified)  DTI:  PSP-RS vs PSP-P  Lower FA in SCPs  PSP-P vs PD  Lower FA in SCPs and midbrain  PSP (O1) vs PSP (O2)  Lower midbrain FA  PSP (O2) vs PD  Lower midbrain and SCP FA  Midbrain area and SCP width correlated with FA values in each region |
| Albrecht et al 2019^60^ | Meta-analysis of 18 studies  315 PSP  393 HC  809 PD (from 29 gray matter and 75 white matter studies)  Studies included with whole-brain approach VBM | MRI | Grey matter analysis  White matter analysis | 4.0 ± 1.0 | PSP vs HC  SDM analysis  Regional gray matter atrophy in midbrain and white matter atrophy in the SCPs + R MCP  Anatomical likelihood analysis  Gray matter atrophy of cerebellum and white matter atrophy of midbrain, pons, SCP  Overlap analysis  Gray matter atrophy clusters in midbrain and white matter clusters in SCP, MCP, midbrain  PSP vs PD  Subtraction analysis  Midbrain white matter atrophy  No infratentorial gray matter atrophy |
| Cope et al 2018^61^ | 17 PSP-RS  17 AD  12 HC | PET  3T MRI | [18F]AV-1451  Resting state fMRI  Whole brain graph theoretical analysis includes brainstem, cerebellum, subcortical structures | unspec | PSP-RS vs AD / HC  Increasing tau burden in midbrain and deep nuclei associated with strengthened cortico-cortical functional connectivity |
| Passamonti et al 2018^62^ | 16 PSP-RS  13 HC  16 AD | PET  3T MRI | [11C]PK11195  *Marker of microglial activation* |  | PSP-RS vs AD / HC  No significant differences in brainstem or cerebellum (dentate/grey matter)  There was, however, a correlation between PSPRS and midbrain/pons uptake |
| Taniguchi et al 2018^63^ | 11 PSP  24 PD  10 HC | 3T MRI | Neuromelanin sensitive MRI of the substantia nigra pars compacta  Midbrain volume | 5.8 +/-3.3 | PSP vs PD / HC  Smaller substantia nigra pars compacta  Lower midbrain volume  PSP vs PD  AUC 0.85 (neuromelanin MRI)  AUC 0.96 (midbrain volume)  AUC 0.99 (combined)  No correlation between substantia nigra pars compacta or midbrain volume and clinical characteristics in PSP |
| Constantinides et al 2018^64^ | 24 PSP (PSP-RS probable)  9 CBD  19 MSA  18 PD  15 HC | 1.5T or 3T MRI | Simple linear MRI brainstem measurements  No measure of medulla |  | PSP vs HC / PD / MSA / CBD  Smaller midbrain and SCP diameters  Mild pons and MCP atrophy too |
| Lipp et al 2018^65^ | 20 PSP  17 PD  12 HC | 1.5T MRI | Brain elasticity  \|G*\| and φ  ROI include mesencephalon | 5.5 ± 2.3 | PSP vs HC / PD  \|G*\| lower in mesencephalon  PSP vs HC  Φ lower in mesencephalon |
| Seki et al 2018^66^ | 12 PSP-RS  12 PSP-P  20 PD  23 HC | 3T MRI | DTI  Voxel-based morphometry  voxel based analysis of entire infratentorial region  Focus on ROI of the dentatorubrothalamic tract (DRTT) | 2.4+/-1.7 | PSP-RS vs PD  Increased MD / decreased FA in midbrain tegmentum and pons white matter loss  Increased MD / decreased FA in SCP decussation, SCPs, dentate nucleus  Increased MD in cerebellar cortex and white matter  Cerebellar grey matter loss and cerebellar (dentate)/ SCP/brainstem white matter loss  PSP-RS vs HC  Increased MD / decreased FA in midbrain tegmentum and pons white matter loss  Also increased MD in MCP  Left MCP white matter loss  Increased MD / decreased FA in SCP decussation, SCPs, dentate nucleus  Increased MD in cerebellar cortex and white matter  Cerebellar grey matter loss and cerebellar (dentate)/ SCP/brainstem white matter loss  PSP-P vs HC/ PD  Increased MD / decreased FA in midbrain tegmentum and pons white matter loss  Reduced FA in SCP, dentate nucleus  Cerebellar grey matter loss and cerebellar white matter loss  PSP-RS vs PSP-P  Increased MD in SCPs and decussation  Reduced FA in dentate nucleus and midbrain tegmentum  Cerebellar white matter loss  MD increase in DRTT correlates with disease duration in PSP-RS. No significant correlations in other groups |
| Nigro et al 2018^55^ | 20 PSP-RS  21 PD  23 HC | 3T MRI | DWI  Track density imaging | 3.1 +/- 1.37 | PSP vs HC / PD  Decreased track density in brainstem  Decreased track density in cerebellum  Using whole-brain support vector machine approach PD differentiated from PSP-RS with AUC 0.82, which increased to 0.98 if only considering superior cerebellar peduncles  No significant correlation with behavioural data |
| Whitwell et al 2018^67^ | Longitudinal (2x scans over 12 months)  16 PSP (all prob, 14 PSP-RS, 1x PSP-P, 1x PSP-PAGF)  39 HC | PET  3T MRI | Tau tracer  [18F]AV-1451  Includes midbrain, pons, dentate nucleus | unspec | PSP vs HC  Significantly increased SUVR in dentate nucleus  Increased midbrain uptake  No significant uptake changes over time |
| Yu et al 2018^68^ | Meta-analytic connectivity modelling using brainmap database for control group  176 PSP and 404 iPD from prior 2014 VBM study | MRI | fMRI  Meta-analytic connectivity modelling  Seeds based on previous VBM study | unspec | PSP vs ‘control’  MACM of thalamic, insular, striatum seeds involves co-activity of cortical, striatal areas and cerebellum  Overlap in co-activity among PSP seeds within cerebellar hemispheres and vermis  PSP vs PD  Greater function co-activation in midbrain, cerebellar hemispheres and vermis  ?Compensatory cerebellar changes |
| Coakeley et al 2018^69^ | 6 PSP  6 PD  10 HC | PET | [18F]AV-1451  SUVRs - calculated from cerebellar cortex | 4 +/- 1.41 | PSP vs HC  Lower substantia nigra SUVR  *off target (neuromelanin) binding noted previously*  PSP vs PD  No difference |
| Kamada et al 2018^70^ | 6 PSP-RS  22 PD  2 MSA-P  6 CBS  20 other diseases | MRI  Cardiac MIBG | MRI planimetry for MRPI calculation | 4 | PSP vs MSA-p / other disease  Decreases heart/mediastium ratio and increased washout rate  Relationship between H&Y and pons area (not midbrain area, SCP width or MRPI)  They suggest that central autonomic dysfunction may result from brainstem atrophy, particularly if it includes the pons or medulla given relevant regions to sympathetic control |
| Talai et al 2018^71^ | 21 PSP-RS  52 PD | 3T MRI | DTI | 5.9 +/- 3.3 | PSP vs PD  Top 17 brain regions significantly higher brainstem MD, AD and RD  Other infratentorial regions possible examined, but not included in article  Using top 17 variables automatic classification has an AUC of 0.913 |
| Rosskopf et al 2017^72^ | 22 PSP-RS  12 PSP-P  35 HC | 1.5T MRI | Resting-state fMRI  In the default mode, midbrain and motor networks  VBM | 44.1 months +/-24.9 | PSP (all) vs HC  VBM showing no significant white matter change. There was significant grey matter loss (regions not specified)  Increased thalamocerebello-midbrain connectivity  PSP-RS vs PSP-P  No significant differences on VBM  No significant differences in functional connectivity (affecting brainstem/cerebellar regions)  Peak eye velocity significantly correlated with reduced midbrain intrinsic functional connectivity |
| Gellersen et al 2017^73^ | Meta-analysis of cerebellar GM VBM  9 PSP studies included | MRI | ALE | unspec | PSP vs HC (ALE)  GM vol loss in left anterior lobe (culmen of vermis ~ lobules I-IV), left posterior lobe (crus I,II, lobule VIIb), right posterior lobe (lobule IX) |
| Passamonti et al 2017^74^ | 19 PSP-RS  15 AD  13 HC | PET | 18F-AV-1451  ROI approach (including midbrain and dentate nucleus)  Then SVM | unspec | PSP vs HC  Increased binding in midbrain and dentate nucleus  PSP vs AD  Increased binding in midbrain  Also did autoradiographic study highlighting off target binding / lower affinity in PSP |
| Zanigni et al 2017^75^ | 25 PSP-RS  47 PD  9 MSA-C  9 MSA-P  27 HC | 1.5T MRI | DTI + tractography  Vertex based cortical thickness analysis  Didn’t include ICP | Median 3.9 (0.5-9.4) | No significant difference in cortical thickness in brainstem/cerebellum  PSP vs PD  Decreased fractional anisotropy and increased mean diffusivity in infratentorial white matter tracts  PSP vs HC / MSA-P / MSA-C  Decreased fractional anisotropy and increased mean diffusivity in superior cerebellar peduncles |
| Gorges et al 2017^76^ | 30 PSP  36 PD  18 MSA  23 HC | 1.5T MRI | DTI - whole brain  Whole brain-based voxelwise spatial statistics | 3 (1-4) | PSP vs HC  Decreased FA in SCPs, MCPs and pontine crossing tract  Correlation between FA in brainstem and horizontal peak eye velocities  Correlation between FA in midbrain and vertical peak eye velocity |
| Brendel et al 2017^77^ | 11 PSP-RS  1 PSP-PNFA  9 HC | PET  Tau tracer | [18F]- THK5351 | 36+/-19 months | PSP vs HC  Increased midbrain and medulla oblongata uptake  Strongest discrimination using midbrain uptake  Correlation of PSPRS with midbrain uptake  No correlations with SEADL or disease duration  No correlations with uptake in other regions |
| Ge et al 2017^78^ | Chinese cohort:  20 PSP  20 MSA  40 PD  American cohort:  10 PSP  10 MSA  20 PD  10 HC | FDG PET | SPM / PCA  validity of PSP related pattern | 3.1+/-1.7 combined | PSP vs HC  In both groups - reduced uptake in midbrain  Increased cerebellar uptake in combined/Chinese cohort (culmen and dentate)  More pronounced in Chinese cohort >American |
| Sakurai et al 2017^79^ | 12 PSP  12 PD  13 HC  13 MSA-P | 1.5T MRI | PRESTO MRI  (Principles of Echo-Shifting with a Train of Observation) | 5.5+/- 3.2 years | PSP vs HC / PD / MSA-P  Lower signal intensity ratio (SIR) in red nucleus in PSP suggesting iron deposition  No significant signal intensity difference in the dentate nucleus |
| Mueller et al 2017^80^ | 20 PSP  20 HC | 3T MRI | VBM  SVM training and classification | unspec | PSP vs HC  Reduced grey matter density in the brainstem (midbrain, pons, medulla)  No significant grey matter atrophy in cerebellum, however relevant voxels in cerebellum for support vector machine classification |
| Whitwell et al 2017^81^ | 10 PSP (probable)  50 HC  10 AD | PET  Tau tracer [18F]AV-1451 | ROI analysis | 3.5 (2.5-4) | PSP vs HC / AD  Increased uptake in midbrain  Increased uptake in dentate nucleus  No significant increase in pons  Correlation with PSPRS |
| Agosta et al 2017^82^ | 21 PSP-RS  36 HC | 1.5T MRI | Cortical thickness  Diffusion tensor imaging  Midbrain volume  Voxel-wise WM change maps  Longitudinal: ~1.4 years  ROI includes MCP, SCP (no ICP) | 2.9±1.2 | PSP-RS vs HC  Smaller midbrain vol at baseline  Diffusion changes reflecting white matter damage in superior cerebellar peduncles, cerebellum, midbrain, pons.  MD increased in SCP over time  Midbrain volume loss over time  Midbrain vol loss correlated with UPDRS. Neg correlation with MMSE.  No correlations with PSPRS |
| Lee et al 2017^83^ | 48 PSP  (25 RS, 23 PAGF)  39 PD  34 HC | 3T MRI | Whole and segmented cerebellar volumes | PSP-RS 34.6 months +/-24.6  PSP-PAGF 43.3 months +/-33 | PSP vs PD / HC  Lower cerebellar volume  Decreased volume in crus I, lobule VIIIa, VIIIb  Shorter SCP and MCP width  PSP-RS vs PSP-PAGF  No significant cerebellar volume differences  Shorter width of superior cerebellar peduncle (and higher MCP/SCP ratio)  SCP/midbrain measures did not seem to correlate with whole or segmental cerebellar volume |
| Lee et al 2017^84^ | 24 PSP  20 HC | 3T MRI | R2* values for five brain regions (putamen, globus pallidus, substantia nigra, subthalamic nucleus, dentate nucleus) | 5.9 +/- 4.3 | PSP vs HC  Increased R2* in all five brain regions (including substantia nigra and dentate nucleus)  UPDRS scores of unilateral tremor correlated with R2* of ipsilateral substantia nigra and dentate nucleus |
| Pan et al 2017^85^ | Meta-analysis of 18 VBM studies  284 PSP (predominantly RS)  364 HC | MRI  9/18 1.5T  8/13 3T  1/13 1.5/3T | Voxel-wise meta-analysis, specifically assessing grey matter | 2.5-4.8 in 15/18 | PSP vs HC  Grey matter loss in midbrain  Grey matter loss in left anterior cerebellum (lobule III,IV,V) |
| Ofori et al 2017^86^ | 71 PSP  63 MSA  184 PD  107 HC | 3T MRI | Diffusion weighted imaging of substantia nigra (SN)  Single tensor metrics - fractional anisotropy (FA) and mean diffusivity (MD)  Bi-tensor metrics - free water, corrected fractional anisotropy, corrected mean diffusivity | unspec | Anterior SN  PSP vs HC / PD  Bi-tensor: Higher free water / corrected FA  Single tensor: Lower FA  Posterior SN  PSP vs HC / PD / MSA  Bi-tensor: Higher free water  PSP vs HC  Single tensor: Higher MD  Posterior free water correlated with UPDRS-III, H&Y, cognitive status  Posterior MD correlated with UPDRS III, H&Y at 2/4 sites  Anterior free water correlated with motor severity and H&Y |
| Sako et al 2017^87^ | Meta-analysis of 6 studies of SCP size (5x width, 1x vol) and 3 studies of ADC  Size  142 PSP  480 PD  41 MSA  ADC  67 PSP  44 PD  51 MSA | Mostly 1.5T MRI  2x 3T | Size: width or volume  DWI: ADC | Range 3.2-13.3 for all diseases | PSP vs MSA / PD  SCP size smaller, however heterogeneous  No significant difference in ADC |
| Sugiyama et al 2017^88^ | 8 PSP  39 PD  13 MSA  34 HC | 3T MRI | SWI  Phase difference enhanced imaging (PADRE) to look at the boundary between the crural fibers and the substantia nigra (BCS) | 7.2+/-3.3 | PSP vs HC  More likely to have bilateral obscuration of BCS and absence of dorolateral nigral hyperintensity on SWI  PSP vs MSA  No significant differences in dorolateral nigral hyperintensity  PSP vs PD  More likely to have bilateral obscuration of BCS  No difference in dorsolateral nigral hyperintensity  Bilateral BCS accuracy 83.3% |
| Brown et al 2017^89^ | 12 PSP  20 HC | 3T MRI | Voxel-based morphometry  Task-free fMRI of rostral midbrain tegmentum ICN  Longitudinal - 6 months | unspec | Midbrain ICN module 3 involving cerebellum and brainstem nodes shows reduced functional connectivity at baseline and longitudinally  No significant cerebellar GM/WM atrophy on VBM  Atrophy of SCP/midbrain over time |
| Schonhaut et al 2017^90^ | 33 PSP  26 PD  46 HC | PET  3T MRI | 18F-flortaucipir (18F-AV1451)  Voxelwise and ROI  11C-PiB (positive scans excluded) |  | PSP vs HC / PD  Increased uptake in midbrain, red nucleus, substantia nigra  Not pons  Increased uptake in dentate nucleus, cerebellar white matter  No correlation with disease severity |
| Ito et al 2017^91^ | 16 PSP  41 PD  6 MSA-P  7 MSA-C  20 HC | 3T MRI | Diffusion kurtosis imaging  QSM - putamen and globus pallidus | 1.5 (1-3) | PSP vs PD / HC / MSA  Lower midbrain to pons diffusion ratio  PD vs PSP - AUC 0.91 using this ratio |
| Pan et al 2017^85^ | 19 PSP  11 CBS  14 HC | 3T MRI | Resting state fMRI  Volumetry | 3.21+/-1.8 | PSP vs HC  Smaller dentate nucleus area  fMRI  Left cerebellum affected by reduced thalamic functional connectivity  Lower dentate nucleus functional connectivity with basal ganglia, thalamus and prefrontal cortex  PSP vs CBS  Post-hoc analysis showed greater reduction in dentate nucleus area compared with CBS  No significant correlations |
| Nicoletti et al 2017^92^ | 21 PSP-RS  9 PSP-P  20 PD  30 HC | 3T MRI | Volumetric and diffusion tensor imaging of SCP | PSP-P  6.3+/-3.7  PSP-RS  3.1+/-1.4 | PSP-RS vs HC / PD  Lower SCP volume  Lower SCP FA  Higher SCP MD  PSP-P vs HC  Lower volume, but no significant difference in FA/MD  PSP-P vs PD  Lower volume  Lower FA  No significant difference in MD  PSP-P vs PSP-RS  No significant difference in volume  Higher FA, lower MD on the left  Discriminant analysis of these metrics could distinguish with accuracy of 70% |
| Bologna et al 2016^93^ | 18 PSP  13 HC | 3T MRI | VBM  ROI  Not including cerebellum | 1-7 years | PSP vs HC  Lower white matter volumes in brainstem  No correlation between blinking variables and midbrain atrophy |
| Huppertz et al 2016^94^ | 106 PSP-RS  204 PD  60 MSA  73 HC | 1.5T MRI | Atlas-based volumetry  Support vector machine (SVM) classification of single subjects (uses combination of supra and infratentorial structures) | unspec | PSP-RS vs HC  Midbrain, pons, medulla, brainstem atrophy  Superior cerebellar peduncle, cerebellum WM/GM, middle cerebellar peduncle atrophy  For SVM  PSP-RS vs HC accuracy 91.1  PSP-RS vs PD 85.3  Midbrain, midbrain tegmentum plane, superior cerebellar peduncle and pallidum contributed most  Not included in table as not actually assessing for significant difference on individual anatomical level |
| Scherfler et al 2016^95^ | 30 PSP  (20 PSP-RS, 10 PSP-P)  40 PD  40 MSA | 1.5T MRI | Volumetric analysis of 22 subcortical regions  Incluldes brainstem, cerebellar WM  No peduncles  Training set  Then Validation set (to test decision tree prediction model)  Did not include medulla | 2.8+/-1.7 | PSP vs HC  Reduced WM cerebellar volume, no significant asymmetry  Reduced midbrain volume  PSP vs PD  Reduced WM cerebellar volume, no significant asymmetry  Reduced midbrain and pons volume  PSP vs MSA / PD -  midbrain volume loss helps to differentiate in decision tree (AUC 0.95) |
| Upadhyay et al 2016^96^ | 19 PSP  11 CBS  14 HC | 3T MRI | Grey matter: automated surface-based analysis  DTI | 3.21 ± 1.8 | PSP vs HC  Lower brainstem volume  Widespread FA reduction  PSP vs CBS  Fewer abnormalities in DTI measures  No significant correlation between clinic and morphometric measures  MMSE correlated with mean FA (not specific region) |
| Caso et al 2016^97^ | 23 PSP-RS  15 HC | 3T MRI | Cortical thickness and DTI measures of white matter tracts of interest  TBSS | 4.2 ± 3.1 | PSP-RS vs HC  Lower midbrain volume  Increased MD in right SCP, pontine crossing fibres  No MCP / ICPs  No significant correlation with cognitive measures |
| Cho et al 2016^98^ | 14 PSP  15 PD  15 HC  *PSP included if nigrostriatal degeneration on PET first* | PET  Tau tracer [18F]AV-1451  3T MRI | VOI template +PET  Dentate and SN included | 4.5 +/- 3.1 | PSP vs HC  Increased tau tracer uptake in midbrain  Increased tau tracer uptake in dentate nucleus  PSP vs PD  Increased uptake in midbrain, substantia nigra  No significant difference in uptake in cerebellum |
| Coakeley et al 2016^99^ | 6 PSP  6 PD  10 HC | PET | Tau tracer [18F]AV-1451  SUVRs in ROIs including dentate nucleus (no brainstem) | 4+/-1.41 | PSP vs HC / PD  No significant difference  No significant correlation |
| Zhang et al 2016^100^ | 35 PSP  25 CBS  23 HC | 3T MRI | DTI  6 month interval scans  ROI | 5.2+/-4.3 | PSP vs HC  Reduced FA in SCPs over time  Increased axial and radial diffusivity in midbrain  PSPRS ocular subscore correlated with increase in axial diffusivity in SCPs |
| Planetta et al 2016^101^ | 18 PSP  18 MSA  18 PD  18 HC | 3T MRI | DTI - bi-tensor diffusion analysis model  ROI: SN (ant, post), RN, PPN, dentate nucleus, MCP, SCP, cerebellar lob V, VI, vermis (VIIIa, VIIIb, IX) | unspec | PSP vs HC  Increased free water in ant/post SN, RN, PPN, DN, MCP, SCP, cerebellar vermis / lobules V/VI  Decreased free water corrected FA in SCP, increased in cerebellar vermis  PSP vs PD  Increased free water in post SN, RN, PPN, DN, SCP, cerebellar lobules V/VI  Decreased free water corrected FA in SCP, increased in cerebellar vermis  PSP vs MSA  Increased free water in posterior SN, SCP, PPN, RN  Decreased free water corrected FA in SCP  Whole group correlations  SCP free water values correlated negatively with MoCA  UPDRS III positive correlated with free water in lobule V and vermis |
| Dutt et al 2016^102^ | 55 PSP  33 CBS  30 HC  Also cohort of from davunetide trial  102 HC  226 PSP-DAV  *Longitudinal*  37 PSP  22 CBS | MRI | Volumetry  Voxel-level differences in grey and white matter | 4.8 +/- 3.1 | PSP / PSP-DAV vs HC  Midbrain, pons  Over 6 mths dorsal midbrain, pontine tegmentum vol loss  Over 12 months vol loss of whole midbrain, pons  Bilateral cerebellar white matter atrophy  Over 12 months vol loss of cerebellar white matter and all 3 peduncles  PSP vs CBS  Lower cerebellar white matter volume  No significant difference over 6 and 12 months  Annual midbrain, pontine volume loss correlates with PSPRS  PSP vs CBS  Lower midbrain and posterior medulla volume  No significant difference over 6 and 12 months |
| Burciu et al 2016^103^ | 19 PSP  46 PD  13 MSA  34 HC | 3T MRI | Task based fMRI  ROI including ipsilateral superior cerebellum (lobules V-VI)  Longitudinal 1 year | 3.15+/-2.95 | PSP vs HC  Reduced mean signal change in cerebellum (along with all other striatal/cortical ROI)  Significantly declined at 1 year  PSP vs MSA/PD - no significant differences  Indicating distinct pattern of functional change in cerebello-thalamo-cortical loops |
| Piattella et al 2015^104^ | 16 PSP  16 HC | 3T MRI | Volumetric and DTI  ROI for DTI - doesn’t include cerebellum | Unspec  ~3 years (difference between mean age of onset and age) | PSP vs HC  Grey matter loss in left cerebellum  Lower regional brainstem volume  Decreased FA in cerebellar peduncles  No significant correlations with MMSE, PSPRS, FAB, UPDRS, H&Y |
| Piattella et al 2015^105^ | 19 PSP  12 HC | 3T MRI | Resting state fMRI  Seed regions thalami, caudate, putamen, and pallidum nuclei (obtained from T1 scan)  DTI  (mean values - regions not specified) | unspec | PSP vs HC  Smaller brainstem volume  Decreased functional connectivity in the posterior lobe of the cerebellum (crus I, lobules VI, VIIb, VIIIa, VIIIb, IX) using caudate or putamen maps  Decreased functional connectivity in crus I, lobules VI, VIIb, VIIIa in pallidum map  Ie. overlapping crus I, lobule VI, VIIb, VIIa in 3/5  connectivity maps |
| Wang et al 2015^106^ | 24 PSP  23 HC | 3T MRI | VBM of T1 weighted FLAIR looking for regions of grey matter atrophy | 3.87 ± 2.62 | PSP vs HC  Significantly decreased grey matter density in left hemisphere of cerebellum, midbrain, pons, red nucleus |
| Reiter et al 2015^107^ | 22 PSP  22 MSA  104 PD  42 HC | 3T MRI | SWI of substantia nigra | 1.64 +/-1.15 | PSP vs HC  More likely to have absence of dorsolateral nigral hyperintensity bilaterally (accuracy 100%)  More likely to have unilateral absence of dorsolateral nigral hyperintensity (accuracy 98.2%)  PSP vs PD / MSA  Did not compare between these groups - combines them to compare with HC - also significant |
| Fukui et al 2015^108^ | 35 PSP  33 PD | 1.5T MRI | CSF flow study  Calculated using 15 images in an equidistant MRI sequence taken throughout cardiac cycle  oblique plane transversal to the midbrain aqueduct to measure aqueduct | 49.6 ± 35.8 months | PSP vs PD  Midbrain atrophy with dilation of midbrain aqueduct  Reduced mean velocity of CSF flow  Also reduced range (Vheight lower - which represents the difference between max and min velocity) |
| Meijer et al 2015^109^ | 3 PSP  12 MSA-P  3 DLB  1 CBD  30 PD  diagnoses made ~28.3 months after MRI | 3T MRI | Manual measures  DTI - TBSS  ROI – includes SCP/dentate nucleus, not other brainstem regions | 28.1 (1.6) for collective (PSP, MSA, DLB, CBD) group | PSP vs PD / MSA-P / CBD / DLB  Higher mean diffusivity in midbrain  Higher mean diffusivity right superior cerebellar peduncle, but not dentate nucleus |
| Zanigni et al 2015^110^ | 21 PSP  15 MSA  21 PD  14 HC | 1.5T MRI | MR spectroscopy of the left cerebellar hemisphere | 4 (1-11) | PSP vs PD  Lower NAA/Cr  PSP vs HC  Lower NAA/Cr and NAA/ml |
| Surova et al 2015^111^ | Cohort 1:  8 PSP  30 HC  Cohort 2:  27 PSP  21 HC  10 PD  11 MSA-P | 3T MRI | DTI  ROI + TBSS  Tractography  Volumetry  No cerebellar ROI - but DRTT tract included | Cohort 1:  5 (4-7)  Cohort 2  3 (3-4) | Cohort 1: PSP vs HC  Higher mean diffusivity (MD) in the midbrain and right dentatorubrothalamic tract (DRTT)  Lower fractional anisotropy (FA) in superior cerebellar peduncle (SCP), midbrain and right DRTT  Cohort 2 (validation):  PSP vs HC  higher MD in pons, midbrain, bilat DRTT, SCP, red nucleus  Lower FA in midbrain, blat DRTT  PSP vs PD  Higher MD in midbrain, bilat DRTT, SCP  Lower FA in SCP, midbrain bilat DRTT  PSP vs MSA-P  Higher MD in midbrain, bilat DRTT  Lower FA in SCP, midbrain, bilat DRTT  Midbrain ROC 0.90  R DRTT ROC 0.94  Impaired balance correlated with MD in R DRTT |
| Burciu et al 2015^112^ | 20 PSP  20 PD  20 HC | 3T MRI | fMRI (BOLD)  *Force control paradigm*  Voxel-based morphometry | 30.6 +/- 31.6 months | PSP vs HC  No BOLD signal change in brainstem  Reduced white matter volume in ventral tegmental area  Reduced BOLD signal in lobules I through IV, V, and VI  Increased BOLD signal in ipsilateral IX and contralateral crus I  Reduced white matter volume in ipsilateral superior cerebellar peduncle  No significant grey matter difference in brainstem/cerebellum  PSP vs PD  No BOLD signal change in brainstem  Reduced white matter volume in ventral tegmental area  Increased BOLD signal in posterior vermis VI and vermis VIIIa/b, IX bilaterally, and crus I/II contralaterally  Reduced white matter volume in ipsilateral superior cerebellar peduncle  No significant grey matter difference in brainstem/cerebellum |
| Ito et al 2015^113^ | 5 PSP  4 MSA  5 PD  6 HC | 1.5T MRI | Diffusion kurtosis imaging (DKI)  ROI | Median 2.5 (1-2.5) | PSP vs HC / PD / MSA  Lower M/P  Mean kurtosis decreased in midbrain  Mean diffusivity increased in midbrain tegmentum / pontine crossing tract  No significant differences in fractional anisotropy |
| Yu et al 2015^114^ | Meta-analysis of VBM studies  176 PSP patients in total | MRI | VBM (grey matter)  ALE  Contrast analysis  Conjunction analysis | unspec | ALE  PSP vs controls  GM reduction in thalamus with extension to the midbrain  No other infratentorial regions |
| Sadowski et al 2015^115^ | 20 PSP  13 CBS | Transcranial ultrasound | Lenticular nucleus and SN echogenicity (iso or hyper)  SN area | 3.9+/-2.3 | PSP vs CBS  Smaller SN area  More likely to have LN hyperechogenicity (9/20)  CBS vs PSP  More unilateral SN hyperechogenicity  (no hyperechogenicity in PSP) |
| Worker et al 2014^116^ | 16 PSP-RS  17 MSA  14 PD  17 HC | 1.5T MRI | DTI - TBSS | 5.2 +/-2.5 | PSP vs HC / MSA  Increased MD in SCP  No difference in FA  PSP vs PD  Reduced FA, increased MD in SCP |
| Ohtsuka et al 2014^117^ | 53 ‘early parkinsonism’  13 PSP  10 MSA-P  30 PD  22 HC | 3T MRI | Neuromelanin sensitive MRI  Contrast ratio  Substantia nigra and Locus coeruleus | Median 2 years (0.5-3) | PSP vs HC  No significant difference  PD vs PSP  Lower contrast ratio laterally and centrally in SNc and LC  MSA-P vs PSP  Lower contrast ratio laterally and centrally in SNc |
| Tessitore et al 2014^118^ | 18 PSP  18 HC | 3T MRI | White matter hyperintensity (WMH) volume  DTI  TBSS | 3.38+/-1.57 | PSP vs HC  Higher WMH volume  Specific diffusivity changes in superior cerebellar peduncles  Some cognitive, phonological, oculomotor and motor measures significantly correlated with cerebellar and superior cerebellar peduncle changes  No significant correlation between imaging and disease duration, UPDRS or MMSE |
| Shao et al 2014^119^ | Meta-analysis (1995-2013)  PSP (9 studies)  PD (24 studies) | 1T, 1.5T or 3T MRI | VBM grey matter | unspec | PSP vs PD  Midbrain volume loss on subtraction analysis  No significant difference in cerebellum |
| Hara et al 2014^120^ | 20 PSP  24 PD  13 MSA-P  18 MSA-C  24 HC | 3T MRI | Diffusion imaging - RESOLVE (readout segmentation of long variable echo-trains)  Specifically decussation of SCP | 3.8 +/- 2.0 | PSP vs HC / PD / MSA  Significantly more isointense signal, however 50% still had hyperintense signal as seen in control group  Therefore low sensitivity 50%, but high specificity 100% |
| Hughes et al 2014^121^ | 13 PSP  13 HC | 3T MRI | VBM | 4.3 +/- 3.1 | PSP vs HC  White matter atrophy in midbrain and upper pons, surrounding the inferior colliculus/cerebral peduncles  (Not medulla)  WM atrophy in ‘cerebellar tracts’  No GM atrophy in cerebellum |
| Whitwell et al 2014^122^ | 9 definite PSP  9 probable PSP  9 CBS  50 HC | 3T MRI | DTI | 3.1+/-1.2 | PSP vs HC  Reduced fractional anisotropy/ increased mean diffusivity midbrain  Also reduced FA in pons  Reduced fractional anisotropy/ increased mean diffusivity in bilat superior cerebellar peduncles  Also increased MD in cerebellum  PSP vs CBS  Increased MD in midbrain  Reduced FA in left superior cerebellar peduncle |
| Agosta et al 2014^123^ | 37 PSP (35 prob, 2 pos)  41 PD  34 HC | 1.5T MRI | White matter hyperintensity load  Tractography  *5 tracts, not involving cerebellum* | unspec | PSP vs HC / PD  Smaller superior cerebellar peduncles  Also diffusion changes in SCP  Not MCP  Left superior cerebellar peduncle of high relative variable importance (100) for PSPRS |
| Reginold et al 2014^124^ | 6 PSP  12 MSA  18 HC | 1.5T MRI | GRE  ADC  In selected cross-sectional areas (ROI including midbrain, pons, MCP - only looked at midbrain for PSP group) - this was a 1cm2 area of midbrain  Longitudinal - MRIs ~2 years apart | unspec | PSP vs HC  Higher GRE signal in midbrain  No change in GRE signal over time  No ADC abnormality  No significant correlations with motor scores |
| Hutchinson et al 2014^125^ | 7 PSP  7 HC | 1.5T MRI | Superchared T1 weighted technique:  Spin lattice relaxation time in substantia nigra (SN) | unspec | PSP vs HC  Specific topography with medial portion of SN more affected using spin lattice distribution index (SI)  And negative radiological index (RI) which is the opposite to what is seen in PD |
| Gardner et al 2013^126^ | 18 PSP  25 young controls (22-49 yo)  26 older controls (60-70yo) | 3T MRI | ROI-based task-free fMRI - ICN (intrinsic connectivity networks) analysis, seeding with dorsal midbrain tegmentum  (dMT)  VBM | 4.8 +/- 2.3 | PSP vs HC3 (combined)  Reduced connectivity, prominent in cerebellum (dentate, vermis), and other regions  This correlated with clinical severity (CDR sum of boxes score), however one outlier was removed. Also was not significant for UPDRS, PSPRS  VBM larger effect sizes where significant atrophy already present  dMT connectivity larger effect sizes in network regions functionally connected to these areas of atrophy (ie. complementary) |
| Zwergal et al 2013^127^ | 12 PSP  12 HC | FDG PET | Focus on locomotor centers | 3.9+/-2.3 | PSP vs HC  Decreased regional glucose metabolism at rest in the pedunculopontine/cunieform nucleus complex (mesencephalic locomotor region)  also in superior cerebellar peduncle/ mesencephalon  No hypometabolism in the superior vermis (cerebellar locomotor region)  During locomotion:  Gait velocity, step length, PSPRS/gait were associated with increased cerebellar glucose metabolism  (possibly suggesting compensatory mechanism) |
| Salvatore et al 2013^128^ | 28 PSP  28 PD  28 HC | 1.5T MRI | Voxel based pattern distribution  Support vector machine classifier | 3.0+/-1.6 | PSP vs HC / PD  Midbrain/pons voxels among most relevant to classifying PSP |
| Marquand et al 2013^129^ | 17 PSP-RS  14 PD  19 MSA  19 HC | 1.5T MRI | Spoilt gradient echo (SPGR) structural images  Probabilistic pattern recognition approach | 5.3+/-2.4 years | PSP-RS vs HC / PD / MSA  Classifier using midbrain/cerebellum can predict PSP with reasonable accuracy (73.6%) - midbrain (71.7%) better than cerebellum (60.0%) |
| Nicoletti et al 2013^130^ | 17 PSP-RS  10 PD  9 MSA-P  7 MSA-C  10 HC | 1.5T MRI | DWI  specifically looking at mean diffusivity in infratentorial structures | 5.0+/-1.8 | PSP-RS vs PD / HC  Higher median MD values in infratentorial compartment, brainstem, cerebellar vermis  Not cerebellar hemispheres  MSA-P / MSA-C vs PSP-RS  Higher median MD in infratentorial compartment, brainstem, cerebellar hemispheres, but no significant difference in the cerebellar vermis |
| Botha et al 2013^131^ | 38 PSP (18 possible, 17 probable, 3 definite)  26 CBS  3 MSA | FDG-PET  MRI | Volumetry  Rating of “pimple sign” *midbrain hypometabolism* | 3.1 (1.4-4.8) | Definite pimple sign only in PSP group  Significantly smaller midbrain in definite group compared with absent group (combination of PSP, CBS, MSA)  PSP vs CBS / MSA  Pimple sign spec 100% sens 28.95% |
| Kepe et al 2013^132^ | 15 PSP  8 PD  5 HC | PET | [18F]FDDNP  *Tau tracer*  ROI including midbrain, cerebellar white matter + deep nuclei | 4.7+/-2 | PSP vs HC / PD  Increased uptake in midbrain and cerebellar white matter/deep nuclei |
| Josephs et al 2013^133^ | 28 PSP  16 HC | 3T MRI | Longitudinal volumetric study  0, 6, 12, 18, 24 month MRIs  13 regions; midbrain only brainstem structure, cerebellum not included | 3.1+/- 1.2 | PSP vs HC  Midbrain atrophy  AND estimated rate of change per year ~10.553% |
| Giordano et al 2013^134^ | 15 PSP  15 PD  15 HC | 3T MRI | VBM of grey matter | 3.16 +/- 1.3 | PSP vs HC  Reduced grey matter volume left cerebellum (anterior lobe) and right cerebellum (posterior lobe)  PSP vs PD  Reduced grey matter volume in left cerebellum (anterior lobe)  Significant correlation between cerebellar atrophy and PIGD, FAB, pVF |
| Sastre-Bataller et al 2013^135^ | 15 PSP (11 RS, 4 P)  45 PD | Transcranial ultrasound | Mesencephalic area and third ventricle width | 58.8+/-30.2 months | PSP vs PD  Smaller mesencephalic area  Increased third ventricle width  PSP-RS vs PSP-P  Third ventricle width greater  Slight correlation between mesencephalic area and duration of disease (r0.39, p<0.001, R2 0.13)  Mean cut off of 4.26cm2 discriminates PD from PSP with accuracy 99.7% |
| Surova et al 2013^136^ | 16 PSP  12 MSA-P  10 PD  16 HC | 3T MRI | DTI + tractography  ROI | 3.5 (2.2–4.0) | PSP vs HC / PD / MSA-P  No significant diffusion differences in MCPs |
| Coon et al 2012^137^ | 10 PSP  3 PLS  20 HC | 3T MRI | DTI  WM tracts, not involving cerebellum | 4 (2-9) | PSP vs HC  Decreased FA in SCP |
| Jesse et al  2012^138^ | 70 PD  170 HC  22 PSP  11 MSA  5 CBS | 1.5T MRI | T2 signal intensity in globus pallidus (GPi) and substantia nigra (SN) | 3.0 (2.4) | PSP vs HC / PD / MSA / CBS  No difference in signal intensity |
| Agosta et al 2012^139^ | 37 PSP (21 PSP-RS, 16 PSP-P)  35 prob 2 pos  42 HC | 1.5T MRI | DTI  Volumetry - VBM  MRPI | PSP-RS 4+/-1  PSP-P 5+/-2 | PSP-RS vs HC  Significant diffusivity differences in superior and middle cerebellar peduncles  White matter loss in cerebellum, superior and middle cerebellar peduncles.  No significant gray matter loss  PSP-P vs HC - no significant differences  PSP-RS vs PSP-P  Right cerebellar crus gray matter loss  Greater white matter loss in superior cerebellar peduncle and midbrain  *Not significant when corrected* |
| Saini et al 2012^140^ | 17 PSP-RS  11 PSP-P  (22 prob, 6 pos)  25 HC | 3T MRI | Volumetry  Does not include cerebellum | 2.6+/-2 | PSP-RS vs HC  No difference in brainstem volume  PSP-P  Lower brainstem volume - not significant after correcting for multiple comparisons |
| Josephs et al 2012^141^ | 6 PSP-CBS  10 PSP  5 CBS | 3T MRI | VBM | unspec | PSP vs HC  White matter loss in midbrain  White matter loss in superior cerebellar peduncle, cerebellum  PSP-CBS vs HC  White matter loss in brainstem  Gray matter loss in cerebellum  White matter loss in cerebellum  PSP-CBS vs PSP  Greater brainstem white matter loss  Greater grey matter loss in cerebellum |
| Tsukamoto et al 2012^142^ | 20 PSP  25 MSA  17 PD  18 HC | 3T MRI | DWI - specifically the regional apparent diffusion coefficient (rADC)  ROI: midbrain, pons, putamen, globus pallidus, caudate nucleus, thalamus, superior cerebellar peduncle, middle cerebellar peduncle, cerebellar white matter, and cerebellar dentate nucleus | 4+/-3 | PSP vs MSA / PD / HC  Higher rADC in midbrain  Not pons  PSP vs MSA / HC  Higher rADC in superior cerebellar peduncle |
| Saini et al 2011^143^ | 24 PSP  (13 RS, 11 P)  26 HC | 3T MRI | DTI  Voxel-wise approach  TBSS  VBM | 2.4+/-2.0 | PSP vs HC  Significant white matter loss in midbrain and pons  (not medulla)  Increased MD in dorsal pons, midbrain  Increased AD in midbrain  Increased RD in midbrain, dorsal pons  Increased MD in superior cerebellar peduncle  Increased AD in superior cerebellar peduncle  Increased RD in superior, middle cerebellar peduncles, cerebellar white matter  PSP-RS vs PSP-P  No significant differences in brainstem or cerebellum  No significant correlation with UPDRS motor scores |
| Kimura et al 2011^144^ | 19 PSP  12 MSA  28 PD  17 HC | SPECT | 99mTc ethylcysteinate dimer  regional cerebral blood flow (rCBF)  SPM | 2.7+/-1.4 | PSP vs HC / PD  No significant differences in cerebellum  PSP vs MSA  Significantly less rCBF in MSA |
| Canu et al 2011^145^ | 5 PSP  13 HC | 1.5T MRI | White matter hyperintensity detection  DTI  VBM | 4.6 | PSP vs HC  Significant diffusivity changes in superior cerebellar peduncles and middle cerebellar pecuncles (though relatively spared)  Reduced white matter volume in midbrain and pons |
| Rolland et al 2011^146^ | 297 PSP  330 MSA | >1T MRI | 32 item ordinal scale  PCA and reliability of factorial scores | 3.9 +/- 1.9 | PSP vs MSA  Meaningful clusters in brainstem and cerebellum (reliability 0.92), midbrain (0.79) |
| Kashihara et al 2011^147^ | 11 PSP  28 MSA  10 CBD  80 PD  9 SCA  54 mild stroke controls | 3T MRI | Manual tracing of Neuromelanin-positive region of substantia nigra pars compacta (SNc) | 3.4+/-2.8 | PSP vs controls  smaller neuromelanin-positive SNc volume |
| Whitwell et al 2011^148^ | 20 PSP  20 HC | 3T MRI | DTI - TBSS | unspec | PSP vs HC  Increased MD in SCP  Decreased FA in SCP (AUC 0.96)  FA in SCP correlated with PSPRS and FBI, MMSE, UPDRS I, II, III - but not significant when adjusted for PSPRS (reflecting disease severity) |
| Whitwell et al 2011^149^ | 18 PSP  18 HC | 3T MRI | fMRI (resting state)  Seed based analysis using thalamus  Voxel-based morphometry  Diffusion tensor imaging | 3.3+/-1.8 | PSP vs HC  Reduced ‘in-phase’ connectivity in cerebellum  No significant connectivity changes in brainstem/cerebellum on assessing the default mode or salience networks  White matter loss in cerebellum, brainstem  Reduced FA in superior cerebellar peduncles  Mean FA in the superior cerebellar peduncles correlated with functional connectivity changes in the thalamus |
| Zwergal et al 2011^127^ | 16 PSP  16 HC | PET  3T MRI | FDG PET  SPM  ROI analysis includes midbrain, vermis among other regions involved in postural /motor control  fMRI (mental imagery of standing) | 4.3+/-2.1 | PSP vs HC  PET  Hypometabolism in mesencephalon, SCP, medulla  No significant correlations between sway path / falls and metabolism in the brainstem (ROI - SN/mesencephalon/pons)  fMRI  Higher frequency of falls associated with BOLD signal decrease during standing in the mesencephalic brainstem tegmentum  Strongest correlation w high sway path values with thalamic/caudate metabolism |
| Focke et al 2011^150^ | 10 PSP  21 PD  11 MSA-P  22 HC | 3T MRI | VBM  SVM analysis | 2.4 +/- 2.3 | PD vs HC - no significant differences  PSP vs PD  Reduced GM vol in cerebellum bilaterally  Reduced WM loss in dorsal aspect of pons, midbrain and cerebellar peduncles  SVM had accuracy 96.8% differentiating PSP from PD |
| Zhao et al 2011^151^ | 13 PSP  18 PD  22 MSA  5 CBD  7 DLB  1 NPH  40 HC | FDG PET |  | unspec | PSP vs HC  Hypometabolism in midbrain in 12/13 (92.31%)  1/13 hypometabolism in superior cerebellar peduncle |
| Agosta et al 2010^152^ | 10 PSP-RS  10 PSP-P  24 HC | 1.5T MRI | VBM | PSP-RS 3.8 (2.5-7)  PSP-P  5.1 (3-10) | PSP vs HC  GM loss in central midbrain, cerebellar lobes  WM loss in midbrain, left SCP  PSP-RS vs PSP-P  Additional GM loss in midbrain, left cerebellar lobe, dentate nuclei  Additional WM loss in midbrain  L SCP loss correlated with disease duration, UPDRS III, letter fluency test  Left cerebellum loss correlated with letter fluency test |
| Teune et al 2010^153^ | 17 PSP  20 PD  21 MSA  10 CBD  6 DLB  15 AD  7 FTD  18 HC | FDG PET | SPM | 2+/-1 | PSP vs controls  Decreased metabolic activity in mesencephalon  Relatively increased metabolic activity in cerebellar white matter |
| Fukui et al 2010^154^ | 18 PSP with obsessive compulsive Sx (OCS)  56 PSP without obsessive compulsive Sx (OCS) | MRI  SPECT  99m Tc-ECD | Middle cerebellar peduncle width | 2.6+/-1.3 | PSP with OCS vs without OCS  More atrophic middle cerebellar peduncles  Decreased uptake in orbitofrontal, caudate, thalamus, cerebellum (OCTC - to reflect frontal-basal ganglia-cerebellum loop)  Reduced uptake in cerebellar tonsils  OCTC uptake and MCP width predicted OCS  MCP width also predicted Y-BOCS |
| Knake et al 2010^155^ | 13 PSP-RS  10 HC | 3T MRI | DTI  TBSS  Whole brain | 3.6 +/- 2.1 | PSP vs HC  Decreased axial diffusivity in brainstem/pons and right substantia nigra  Increase in fractional anisotropy in cerebellum and bilateral superior cerebellar peduncle  Reduced axial diffusivity in cerebellar white matter  Increased radial diffusivity in bilateral superior cerebellar peduncle, cerebellar white matter and vermis  Radial diffusivity in superior cerebellar peduncles discriminates PSP from controls  left: sens 0.92 spec 0.8 PPV 0.86  Right sens 0.92 spec 1.0 PPV 0.91 |
| Messina et al 2010^156^ | 32 PSP  72 PD  15 MSA-P  46 HC | 1.5T MRI | Volumetry  Automated segmentation | 3.53 +/-3.5 | PSP vs HC / PD  Atrophy of cerebellar cortex and brainstem  PSP vs MSA  No significant differences in brainstem/cerebellum |
| Ebentheuer et al 2010^157^ | 27 PSP-RS  7 PSP-P | Transcranial ultrasound | SN echogenicity  Third ventricle width | unspec | PSP-P vs PSP-RS  Significantly more likely to have hyperechogenic SN |
| Wang et al 2010^158^ | 17 PSP  (6 PSP-P, 7 PSP-RS, 4 PSP-PAGF)  17 HC | 3T MRI | DTI - ROI including SN, MCP, unilateral pons, midbrain region between RN and aqueduct) | PSP-P  6.5+/-3.3  PSP-RS 5.7+/-2.1  PSP-PAGF 4.5+/-1.3 | PSP vs HC  No significant difference in FA in pons, midbrain, MCP  Elevated FA in SN  Elevated MD in midbrain  Significant correlation between disease duration / UPDRS III and midbrain FA  PSP-P vs PSP-RS / HC  Higher IVDC (intervoxel diffusion coherence) in MCP |
| Roselli et al 2010^159^ | 8 PSP  15 DLB  15 PD  15 ET  9 HC | PET | 123I-FP-CIT  midbrain specifically | 17.7 +/- 13.9 months | PSP vs HC / ET / PD  Significantly reduced uptake in midbrain |
| Gupta et al 2010^160^ | 12 PSP  11 PD  12 MSA-P  11 HC | 1.5T MRI | SWI - Assessing hypointensity in red nucleus, substantia nigra, dentate nucleus (0-3) |  | PSP vs HC / MSA / PD  Higher hypointensity score for red nucleus, substantia nigra  No significant difference for dentate nucleus |
| Gama et al 2010^161^ | 14 PSP  13 MSA  16 PD  12 HC | 1.5T MRI | Area and diameter  ROI not including cerebellum | 5.7+/-2.28 | PSP vs HC  Smaller SCP width  Smaller midbrain area  No significant difference in pons area  No significant difference in MCP width |
| Gilman et al 2010^162^ | 4 PSP  13 MSA-P  12 PD  22 HC | PET | [11C]PMP  *AChE substrate*  ROI | unspec | PSP vs HC  Lower subcortical K3 (hydrolysis rate of tracer to acetate and choline) in cerebellum, pons, midbrain |
| Lehericy et al 2010^163^ | 10 PSP  9 Gd-PSP  9 HC | 1.5T MRI | VBM, DTI  Voxel-based and ROI  Also MR spectroscopy but only of lenticular nucleus  Did not include medulla | 4.3+/-1.0 | PSP vs HC  Lower grey matter volume in midbrain  Lower white matter volume in midbrain, pons, SCPs, left ICP and cerebellar hemisphere  Increased ADC in white matter of midbrain, SCPs, and right cerebellum  Lower FA in the decussation of SCPs, midbrain and cerebellum  Significant difference on all midbrain metrics as well as smaller pons and MCP width  PSP vs Gd-PSP  Lower FA in white matter of cerebellum and decussation of SCPs  Smaller midbrain and lower M/P  Higher MRPI and lower AP midbrain diameter |
| Vasconcellos et al 2009^164^ | 11 PSP  12 PD  10 HC  7 MSA | 1.5T MRI | MR spectroscopy frontal, lentiform nucleus, midbrain and hippocampus  Diameters of medulla, pons, midbrain, ventricles | Mean 6.6 (3.1) | PSP vs HC  Lower NAA/Cho in midbrain  PSP vs PD  No significant findings in midbrain  PSP vs MSA  Lower NAA/Cho in midbrain |
| Stamelou et al 2009^165^ | 8 PSP  13 HC | 1.5T MRI  PET | VBM  Binding potential  5-HT2A receptor tracer [18F]altanserin binding  VOI analysis  Cerebellum reference | 4.1 +/- 1.4 | PSP vs HC  Increased binding potential in substantia nigra  This correlated with PSPRS, UPDRS III |
| Borroni et al 2008^166^ | 21 PSP  20 CBD  44 FTD  29 AD  10 PD  15 DLB  27 HC | 1.5T MRI | VBM  Assessing correlation with tau 33kDa/55kDa form ratio | 3.5+/-3.1 | PSP with low tau form ratio vs other with normal tau form ratio  Significant clusters of reduced GM in the brainstem  No significant clusters in cerebellum |
| Eckert et al 2008^167^ | Pattern identifying gorup  10 PSP  10 MSA  10 HC | FDG PET | Identifying disease related pattern  SVM and applying to validation group | 2.8 +/- 1.1 | PSP vs HC  Identifies PSP related pattern which includes metabolic decrease in upper brainstem |
| Nicoletti et al 2008^168^ | 28 PSP (14 possible, 14 probable)  15 PD  15 MSA-P  16 HC | 1.5T MRI | DWI of SCP | 3.2+/- 1.7 | PSP vs HC / PD / MSA-P  Higher apparent diffusion coefficient values in superior cerebellar peduncles  This discrimated PSP from PD (spec 100% sens 100%) and MSA-P (sens 96.4% spec 93.3%) |
| Rizzo et al 2008^169^ | 10 PSP-RS  13 PD  7 CBS  9 HC | 1.5T MRI | DWI  ADC average maps  ROI not including brainstem/cerebellum, but does include superior cerebellar peduncles | 4+/- 3 | PSP-RS vs HC / PD  Higher ADC values in superior cerebellar peduncles |
| Park et al 2008^170^ | 14 PSP  11 PAGF  11 HC | FDG PET | SPM | PSP 4 +/-1.7  PAGF 7.2 +/-2.6 | PSP vs HC  Reduced midbrain uptake  PAGF vs HC  Reduced midbrain uptake |
| Herting et al 2007^171^ | 9 PSP + depression  11 MSA + depression  25 HC | FDG PET  1.5T MRI | SPM | 5.4+/-2.34 | PSP vs HC  Decreased uptake in midbrain (as well as frontal regions)  PSP vs MSA  Midbrain hypometabolism  MSA vs PSP  Decreased uptake in cerebellar cortex  Correlation between depression scores and hypometabolism in the fronta regions (not brainstem/cerebellum) |
| Bartels et al 2007^172^ | 5 PSP  10 PD  10 PD de novo  4 MSA  10 HC | PET | [11C]-verapamil  *P-glycoprotein substrate- to assess Blood Brain Barrier efflux transport system potentially resulting in accumulation of toxic compounds*  SPM | 3+/-2 | PSP vs HC  No difference in brainstem or cerebellum  (only basal ganglia) |
| Paviour et al 2006^173^ | 24 PSP  11 MSA-P  12 PD  18 HC | 1.5T MRI | Volumetry  Longitudinal atrophy rate (% tissue loss per annum)  ROI including midbrain, pons, cerebellum | 4.6 +/- 1.6 | PSP vs HC  Greater midbrain % tissue loss per annum  Greater cerebellum % tissue loss per anum  Greater SCP % tissue loss per anum  PSP vs PD  Greater midbrain and pons % tissue loss per annum  Greater SCP % tissue loss per anum  Midbrain atrophy associated with decline in Mattis Dementia Rating Scale-attention sub-test, errors on WCST, decrease in PASAT, MMSE and FAB, increase in UPDRS III  Pons and cerebellar atrophy associated with decreased FAB score  Cerebellar atrophy also associated with decreased MMSE |
| Paviour et al 2006^174^ | 18 PSP  9 MSA-P  9 PD  18 HC | 1.5T MRI | Volumetry  ROI including midbrain, pons, cerebellum | 4.6 +/- 1.6 | PSP vs HC  Lower midbrain volume  Lower SCP volume  Larger third ventricle volume  (not pons or cerebellar vol)  PSP vs PD  Lower midbrain volume  Lower SCP volume  PSP vs MSA-P  Lower midbrain volume  Midbrain volume correlation with UPDRS II,III, H&Y, postural stability, swallowing, gaze palsy score  SCP vol correlation with falls and postural stability |
| Seppi et al 2006^175^ | 14 PSP  17 PD  15 MSA-P  13 HC | SPECT | [123I]-CIT  *DAT tracer*  ROI  SPM | 2.2+/-0.7 | PSP vs HC / PD  Reduced DAT tracer uptake in ventral/dorsal midbrain, pons  PSP vs MSA-P  No significant difference |
| Nicoletti et al 2006^176^ | 16 PSP  16 MSA-P  16 PD  15 HC | 1.5T MRI | DWI – rADC (regional apparent diffusion coefficients)  ROI including pons and MCP | 3.3 +/- 2.5 | PSP vs HC / PD  Increased rADC in pons  No significant difference in middle cerebellar peduncle |
| Blain et al 2006^177^ | 17 PSP  17 MSA  12 PD  12 HC | 1.5T MRI | DTI  Specifically decussation of the SCP (dSCP), MCP and pons  T2 weighted images for measuring diameter | 5.3+/-2.4 | PSP vs HC / PD / MSA  Higher MD in dSCP  This was moderately related to the degree of atrophy (r=-0.62)  MSA vs PSP  MCP/pons significantly different  PSP vs PD  Lower FA in dSCP  PSP vs HC / PD  No differences in pons / MCP  No correlation of these findings with clinical severity |
| Josephs et al 2008^178^ | 13 definite PSP  11 definite CBD  24 HC | 1.5T MRI | VBM | 7 (4-10) | PSP vs HC  Mesencephalic/diencephalic junction, cerebral peduncle of midbrain atrophy  Grey matter atrophy in the lingula of the cerebellum |
| Juh et al 2005^179^ | 8 PSP  8 CBD  22 HC | FDG PET | SPM | unspec | PSP vs HC  Reduced uptake in brainstem/midbrain  PSP vs CBS  Reduced uptake in left brainstem/midbrain |
| Paviour et al 2005^180^ | 19 PSP  10 MSA  12 PD  12 HC | 1.5T MRI | SCP volume | 4.6 +/- 1.6 | PSP vs PD / MSA / HC  Lower volume of SCP |
| Gerhard et al 2005^181^ | 4 PSP (probable)  7 HC  2x PSP had repeat scans at 6 and 10 months, respectively | PET | benzodiazepine binding site tracer (microglia activation)  [11C](R)-PK11195 | 3.5 | PSP vs HC  Increased binding in pons, midbrain, substantia nigra  Increased binding in cerebellum  No longitudinal change |
| Oba et al 2005^182^ | 21 PSP  23 PD  25 MSA-P  31 HC | 1.5T MRI | Mid-sagittal MR used to assess midbrain and pons areas | 33.6 +/- 15.6 months | PSP vs HC / PD / MSA-P  Smaller midbrain area and M/P ratio  PSP vs HC / PD  Smaller pons area too |
| Walter et al 2004^183^ | 8 CBD  13 PSP | Brain parenchyma sonography (11/13 PSP)  1T MRI in 7/13 PSP  CT in 2 PSP | Substantia nigra, brainstem raphe echogenicity  Width of third ventricle | 3.5 +/-2.1 | PSP vs CBD  Smaller substantia nigra, less hyperechogenicity, wider third ventricle (corresponded with MRI measurement)  No difference in brainstem raphe  Width of third ventricle negatively correlated with ACE cognitive Ax, but not with age/disease severity or duration |
| Klein et al 2005^184^ | 10 PSP  9 PD | FDG PET | SPM | 4.7 +/- 3.4 | PSP vs PD  Reduced midbrain / superior colliculus uptake |
| Mishina et al 2004^185^ | 15 PSP  16 HC | FDG PET | SPM | 1-16 years | PSP vs HC  Midbrain hypometabolism  No significant cerebellar hypometabolism, however correlation between cerebellar vermis uptake and UPDRS III |
| Taki et al 2004^186^ | 19 PSP  19 CBD | 1.5T MRI | Volumetry  33 cortical regions each side  Includes midbrain and pons  Also calculated TIV, cerebellar, brainstem volume | unspec | PSP vs CBD  Significant midbrain atrophy  No significant difference in pons, cerebellar volume |
| Groschel et al 2003^187^ | 33 PSP (6 possible, 20 probable, 8 definite)  18 CBD (7 definite)  22 HC | 1.5T MRI | volumetry | 3.6+/- 2.2 | PSP vs HC  Smaller brainstem, midbrain and medulla volumes  PSP vs CBD  Smaller midbrain, pons, medulla volumes |
| Brenneis et al 2003^188^ | 12 PSP  12 HC | 1.5T MRI | VBM | 2.7 +/- 0.9 | PSP vs HC  Mesencephalon atrophy  No significant difference in the cerebellar WM/GM |
| Eckert et al 2003^189^ | 10 PSP  15 PD  12 MSA  20 HC | 1.5T MRI | Manual segmentation (Globus pallidus, Putamen, substantia nigra, caudate, white matter) | 4.2 +/- 2.7 | PSP vs HC / PD / MSA  Lower magnetization transfer ratio in substantia nigra |
| Walter et al 2003^190^ | 9 PSP  16 MSA  25 PD | Transcranial sonography |  | 4.8+/-1.7 | PSP vs PD  Smaller area of echogenic substantia nigra |
| Kato et al 2003^191^ | 8 PSP  12 PD  10 HC | 1.5T MRI | Manual measures | 6.4+/-6.2 | PSP vs HC  Smaller rostral, caudal midbrain tegmentum, inferior colliculus, pontine base and tegmentum  PSP vs PD  Shorter A-P diameter of midbrain tegmentum, larger ratio of interpeduncular fossa to midbrain tegmentum larger |
| Juh et al 2003^192^ | 7 PSP  9 MSA  8 PD  22 HC | FDG PET | SPM | 30+/-18.5 months | PSP vs HC  Midbrain hypometabolism  PSP vs PD  Midbrain hypometabolism  PSP vs MSA  higher metabolism in pons, right brainstem, left cerebellum (likely reflects relative hypometabolic areas in MSA) |
| Hosaka et al 2002^193^ | 12 PSP  12 CBD  12 HC | MRI +  FDG PET | SPM + ROI analysis for asymmetry | unspec | PSP vs HC / CBD  Midbrain hypometabolism |
| Yekhlef et al 2002^194^ | 30 PSP  32 PD  28 MSA | 0.5,1 or 1.5T MRI | Presence or absence of certain signs  Severity score | unspec | PD vs PSP  Less midbrain atrophy and third ventricle dilatation  No difference in rates of pontine atrophy, but less on median severity score  Less cerebellar vermian atrophy  Lower median severity score for cerebellar vermian atrophy, pontine atrophy, third and fourth ventricle dilatation, midbrain atrophy |
| Zhang et al 2001^195^ | 12 PSP  12 CBD | SPECT | 99Tcm-ECD  ROI including brainstem and cerebellar cortex |  | PSP vs CBD  No significant differences in regional cerebral blood flow |
| Warmuth-Metz et al 2001^196^ | 16 PSP  20 PD  12 HC  14 MSA-P | 1.5T MRI | Manual measures of pons, midbrain, collicular plate | 41.9+/-15.6 months | PSP vs HC  Smaller midbrain diameter  Smaller collicular plate  Smaller pons  PSP vs PD  Smaller midbrain diameter  Smaller pons  PSP vs MSA  Smaller midbrain diameter  Smaller collicular plate  No significant correlations |
| Asato et al 2000^197^ | 8 PSP  30 MSA  10 HC | 1.5T MRI | Manual brainstem measures on T1  Divergence of the red nucleus on T2 | 2.3+/-0.93 | PSP vs HC / MSA  Smaller rostral and caudal midbrain diameter  No difference in the pons/medulla  Divergence of RN in all PSP and none of other groups - not statistically compared  MSA vs PSP / HC  Smaller pons |
| Garraux et al 2000^198^ | 22 CBS  21 PSP (prob)  46 HC | FDG-PET | SPM | 4.2 +/- 2.6 years | PSP vs HC / CBD  Midbrain hypometabolism |
| Garraux et al 1999^199^ | 20 PSP  21 HC  6 FTD | FDG PET | SPM  Correlation analysis | 4.4+/- 2.6 | PSP vs HC / FTD  Reduced uptake in midbrain tegmental region  PSP vs HC  Functional connectivity with the midbrain tegmentum was different in the left temp region, parahippocampal gyrus, left cerebellar cortex and left pallidum |
| Schulz et al 1999^200^ | 6 PSP  11 PD  12 MSA-P  17 MSA-C  46 HC | 1.5T MRI | Volumetry (manual) | 2.3+/-0.95 | PSP vs HC  Smaller brainstem volume  No significant difference in cerebellum volume  PSP v MSA-P  No significant difference in cerebellum volume |
| Tedeschi et al 1997^201^ | 12 PSP  10 PD  9 CBS  11 HC | 1.5T MRI | MR spectroscopy  ROI including brainstem | 48+/-9 months | PSP vs HC  Lower NA/Cre in brainstem  Not lower than PD or CBS |
| Karbe et al 1992^202^ | 9 PSP  9 HC | FDG PET | VOI | Range 0.5-3 | PSP vs HC  Reduced in brainstem (midbrain) /cerebellum on region |
| Stern et al 1989^203^ | 6 PSP  12 MSA  37 PD  16 ATYP  24 HC  ATYP: progressive akinetic rigid syndrome, no evidence heterogeneous systems degeneration, failed response to antiparkinons medication | 1.5T MRI | Focal atrophy qualitatively determined - absent, mild, moderate or severe (brainstem/cortex only)  Subcortical / periventricular hyperintensity also qualitatively determined  Substantia nigra signal | 5.3 +/- 0.56 | More brainstem atrophy in combined (MSA, PSP, ATYP) vs PD, but no significant difference between PSP, MSA, ATYP |
| Foster et al 1988^204^ | 14 PSP  21 HC | FDG PET |  | unspec | PSP vs HC  Lower glucose uptake in the brainstem (brainstem including pons +partial midbrain/medulla)  No significant differences in cerebellum |
| Abe et al 1983^205^ | 3 PSP  20 HC  25 SCD  5 shy Drager  10 phenytoin  6 alcohol | CT | Manual measures | unspec | PSP vs HC / SCD / shy Drager / phenytoin / alcohol  Midbrain atrophy on AP measurement |

PSP-RS: Progressive supranuclear palsy – Richardson’s Syndrome

PSP-P: Progressive supranuclear palsy – Parkinsonism variant

PSP-PAGF/PGF: Progressive supranuclear palsy – Pure Akinesia with Gait Freezing variant

PSP-C: Progressive supranuclear palsy – Cerebellar variant

PSP-CBS: Progressive supranuclear palsy – Corticobasal Syndromevariant

PSP-F: Progressive supranuclear palsy –Frontal variant

PSP-SL: Progressive supranuclear palsy – Speech/Language variant

PSP-PI: Progressive supranuclear palsy – Postural Instability variant

PSP-PNFA: Progressive supranuclear palsy – Progressive Non-Fluent Aphasia variant

vPSP: variant Progressive Supranuclear Palsy

Gd-PSP: Guadeloupean Progressive Supranuclear Palsy

PSP-DAV: PSP cohort from davenutide trial

HC: Healthy Controls

PD: Parkinson’s Disease

PIGD: Postural Instability Gait Difficulty

iPD: idiopathic Parkinson’s Disease

iRBD: idiopathic REM sleep Behaviour Disorder

RBD: REM sleep Behaviour Disorder

MSA: Multiple System Atrophy

MSA-P: Multiple System Atrophy – Parkinsonism variant

MSA-C: Multiple System Atrophy – Cerebellar variant

VaP: Vascular Parkinsonism

GGT: Globular Glial Tauopathy

PLS: Progressive Lateral Sclerosis

SCA: Spinocerebellar Ataxia

SCD: Spinocerebellar Degeneration

NPH: Normal Pressure Hydrocephalus

1.5T: 1.5 Tesla

3T: 3 Tesla

7T: 7 Tesla

MRI: Magnetic Resonance Imaging

DTI: Diffusion Tensor Imaging

DWI: Diffusion Weighted Imaging

SYN: Synucleinopathy

FDG: Fludeoxyglucose

PET: Positron Emission Tomography

CT: Computed Tomography

FLAIR: Fluid Attenuated Inversion Recovery

NAA: N-acetylaspartate

Cr: Creatine

mI: myoinositil

Cho: Choline

TBSS: Tract-Based Spatial Statistics

NfL: Neurofilament Light chain

SPM: Statistical Parametric Mapping

PPV: Positive Predictive Value

VOI: Volume of interest

SUVR: Standardised Uptake Value Ratio

MNI: Montreal Neurological Index

AP: Atypical parkinsonism

VBM: Voxel Based Morphometry

CBS: Corticobasal Syndrome

CBD: Corticobasal Degeneration

PSPRS: Progressive Supranucelar Palsy Rating Scale

SEADL: Schwab and England Activities of Daily Living scale

CSF: Cerebrospinal fluid

ROI: Region of Interest

ADC: Apparent Diffusion Coefficient

MD: Mean diffusivity

FA: Fractional Anisotropy

RD: Radial Diffusivity

IVDC: Intervoxel Diffusion Coherence

MRPI: Magnetic Resonance Parkinonism Index

M/P: Midbrain to Pons ratio

P/M: Pons to Midbrain ratio

FTD: Frontotemporal dementia

AD: Alzheimer Dementia

AD-LPA: Alzheimer Dementia – Logopenic/Phonological Aphasia variant

UPDRS: Movement Disorders Society – Unified Parkinson’s Disease Rating Scale

UPDRS I: Movement Disorders Society – Unified Parkinson’s Disease Rating Scale (part I)

UPDRS II: Movement Disorders Society – Unified Parkinson’s Disease Rating Scale (part II)

UPDRS III: Movement Disorders Society – Unified Parkinson’s Disease Rating Scale (part III)

GM: Grey Matter

WM: White Matter

SCP: Superior Cerebellar Peduncle

MCP: Middle Cerebellar Peduncle

ICP: Inferior Cerebellar Peduncle

DRTT: Dentato-Rubro-Thalamic Tract

dMT: dorsal Midbrain Tegmentum

SN: Substantia Nigra

SNImed: Medial part of the Substantia Nigra

SNIlat: Lateral part of the Substantia Nigra

SNIventr: Ventral part of the Substantia Nigra

SNc: Substantia Nigra Pars Compacta

RN: Red Nucleus

PPN: Pedunculopontine Nucleus

DGM: Deep Grey Matter

AUC: Area Under the Curve

ROC: Receiver Operating Characteristic

DLB: Dementia with Lewy Bodies

SNR: Signal to Noise Ratio

bvFTD: behavioural variant Frontotemporal Dementia

nfvPPA: non-fluent variant Primary Progressive Aphasia

svPPA: sematic variant Primary Progressive Aphasia

unspec: unspecified

LC: Locus Coeruleus

MoCA: Montreal Cognitive Assessment
MMSE: Mini Mental State Examination

FAB: Frontal Assessment Battery

FBI: Frontal Behaviour Inventory

pVF: phonological Verbal Fluency

WCST: Wisconsin Card Scoring Test

PASAT: Paced Auditory Serial Addition Test

DAT: Dopamine Transporter

fMRI: functional Magnetic Resonance Imaging

BOLD: Blood Oxygenation Level Dependent imaging

MCI: Mild Cognitive Impairment

OCT: Optical Coherence Tomography

CNR: Contrast to Noise Ratio

ACE-R: Addenbrookes Cognitive Examination – R

SPECT: Single-Photon Emission Computed Tomography

FTLD-TDP: Frontotemporal Lobar Degeneration with TDP-43 immunoreactive pathology

H&Y: Hoehn and Yahr

FW: Free Water

NODDI: Neurite Orientation Dispersion and Density Imaging

RESOLVE: Readout Segmentation of Long Variable Echo-Trains

O1: Vertical supranuclear gaze palsy

O2 Slow velocity of vertical saccades

MIBG: Meta-Iodo-Benzyl-Guanidine

ALE: Activation Likelihood Estimation

SVM: Support Vector Machine

SPM: Spatial Predictive Modelling

PCA: Principal Component Analysis

SSM: Scaled Subprofile Model

SWI: Susceptibility Weighted Imaging

QSM: Quantitative Susceptibility Mapping

PRESTO: Principles of Echo-Shifting with a Train of Observation

PADRE: Phase Difference Enhanced Imaging

GRE: Gradient Echo Sequence

ICN: Intrinsic Connectivity Network

PiB: Pittburgh B

**Supplementary table 2:** MRI studies of established midbrain metrics

| Study | PSP variant  (if specified) | Control/ comparator | Significant difference/  accuracy (highest; if measured) |
| --- | --- | --- | --- |
| Onder et al 2023^206^ | PSP | PD | PSP vs PD - yes (accuracy 83.9%) |
| Shir et al 2023^207^ | Definite PSP in patients with CBS | CBS with other pathology (AD/DLB, AD, CBD, FTLD) | CBS-PSP vs CBS (other) – Yes |
| Muller et al 2023^208^ | PSP-RS | DLB  PD  HC | PSP-RS vs DLB / PD / HC - Yes |
| Miyata et al 2023^209^ | PSP | PD  HC | PSP vs PD / HC - yes  *Using PADRE MRI methodology*  *Highest AUC 0.97 using SCP width* |
| Onder et al 2022^210^ | PSP | iNPH | PSP vs iNPH - no |
| Compta et al 2022^211^ | PSP-RS | HC  PD  MSA  CBD | PSP vs HC / PD / MSA - yes  PSP vs CBD - no |
| Illan-Gala et al 2022^212^ | PSP-RS | CBS  Other pathological Dx (DLB/PD/FTLD/  cerebrovasc) | PSP vs CBS - no  PSP vs other - yes (accuracy 0.87) |
| Madetko et al 2022^213^ | PSP-RS  PSP-P | CBS  MSA-P  HC | PSP-P vs control - yes (accuracy 0.966)  PSP-P vs MSA-P - yes (accuracy 0.882)  PSP-RS vs PSP-P - no |
| Alster et al 2022^213^ | PSP-P | MSA-P | PSP-P vs MSA-P - yes |
| Luca et al 2021^214^ | PSP | PD | PSP vs PD - yes |
| Virhammar et al 2021^215^ | PSP | iNPH  HC | PSP vs HC - yes  PSP vs iNPH - yes (AUC 0.86) |
| Grijalva et al 2021^216^ | PSP-RS  PSP-SL  PSP-P  PSP-CBS  PSP-PGF  PSP-F  PSP-corticospinal | HC | PSP RS / SL / CBS vs HC - yes  PSP-PGF vs HC - no  PSP-RS vs SL/PGF - yes  PSP-CBS vs PGF - yes |
| Kannenberg et al 2021^217^ | PSP | PD  HC | PSP vs HC - yes (accuracy 95.6%)  PSP vs PD - yes (accuracy 95.6%) |
| Heim et al 2021^218^ | PSP (PSP-RS + PSP-P) | PD  MSA | PSP vs PD - yes (accuracy 0.98)  PSP vs MSA - yes (accuracy 0.98) |
| Herwig et al 2021^219^ | PSP | PD  HC | PSP vs HC - yes  PSP vs PD – yes (AUC 0.96) |
| Janarthanan et al 2021^220^ | PSP | PD  HC | PSP vs HC - yes  PSP vs PD - yes |
| Cooperrider et al 2020^221^ | PSP-RS | PD | PSP vs PD - yes |
| Oktay et al 2020^222^ | PSP | PD  MSA  HC | PSP vs PD - yes (Akdeniz index accuracy 82% MRPI 80%)  PSP vs MSA - no |
| Ugga et al 2020^223^ | PSP | iNPH  HC | iNPH+PSP vs HC - yes  PSP vs iNPH - no |
| Nigro et al 2020^224^ | PSP-RS  PSP-P | *Non-PSP:*  PD  MSA  HC | PSP-RS vs *non-PSP* - yes  (accuracy 93.6% / 90.1% < 4 years)  PSP-P vs *non-PSP* - yes  (accuracy 86.5% / 85.9% < 4 years) |
| Picillo et al 2019^225^ | PSP-RS  PSP-P  vPSP | PD  HC | PSP (combined) vs PD / HC - yes  PSP-RS vs PD / HC(accuracy 91.5 / 97.3%)  vPSP vs PD / HC (accuracy 74 / 74%)  PSP-P vs PD / HC (accuracy 75 / 88.1%)  PSP-RS vs PSP-P - yes (accuracy 77.9%)  PSP-RS vs vPSP - yes (accuracy 68.4%) |
| Quattrone et al 2019^226^ | PSP-P (initial PD Dx) | PD  HC | PSP-P vs PD - yes (accuracy 100%)  *Also longitudinally in PD → PSP-P group* |
| Ahn et al 2019^227^ | PSP-RS | PD  HC | PSP-RS vs HC - yes (accuracy 96.3%)  PSP-RS vs PD - yes (accuracy 94.4%)  *Also longitudinally in pre-PSP-RS → PSP-RS group (accuracy of Pre-PSP-RS vs HC 92.6% and Pre-PSP-RS vs PD 88.9%)* |
| Nakahara et al 2019^228^ | PSP-RS  PSP-PAGF | PD | PSP-RS vs PAGF - yes (accuracy 45.7-91.4%)  PSP-PAGF vs PD - yes (accuracy 30.4-68.6%)  PSP-RS vs PD - yes (accuracy 69.7-86.6%) |
| Sako et al 2019^229^ | PSP | MSA  PD  CBS  HC | PSP vs HC - yes  PSP vs PD / MSA - yes (AUC 0.97) |
| Eraslan et al 2019^230^ | PSP | MSA  PD  HC | PSP vs PD / MSA / HC – yes (AUC 0.956) |
| Constantinides et al 2019^231^ | PSP-RS | iNPH  HC | PSP-RS vs HC - yes  PSP-RS vs iNPH – yes (AUC 0.79) |
| Mueller et al 2018^232^ | PSP | PD  MSA  HC | PSP vs HC / MSA / PD - yes (high spec 97.7-100% / sens lower 37.7-55.3%) |
| Constantinides et al 2018^233^ | PSP-RS | CBD  MSA  PD  HC | PSP-RS vs HC / PD / CBD / MSA - yes  (AUC 0.98) |
| Quattrone et al 2018^234^ | PSP-RS  PSP-P | PD  HC | PSP-RS vs HC - yes (accuracy 100%)  PSP-RS vs PD - yes (accuracy 100%)  PSP-P vs HC - yes (accuracy 98.9%)  PSP-P vs PD - yes (accuracy 96.6%) |
| Kim et al 2017^235^ | PSP | VaP  PD | PSP vs VaP - yes  PSP vs PD - yes |
| Silsby et al 2017^236^ | PSP | PNFA  LPA  HC | PSP vs PNFA / LPA – yes (AUC 0.904)  PSP vs HC – yes (AUC 0.969) |
| Nizamani et al 2017^237^ | PSP | HC  PD | PSP vs HC - yes  PSP vs PD – yes (accuracy 100%) |
| Mangesius et al 2017^238^ | PSP | PD  MSA | PSP vs PD - yes (AUC 0.95)  PSP vs MSA - yes (AUC 0.95) |
| Nigro et al 2017^239^ | PSP | PD  HC | PSP vs HC - yes  PSP vs PD – yes  (probable PSP AUC 1.00)  (possible PSP AUC 0.998) |
| Moller et al 2017^240^ | PSP | PD  MSA-C  MSA-P  HC | PSP vs HC - yes (AUC 0.932)  PSP vs PD – yes (AUC 0.900)  PSP vs MSA-P – yes (AUC 0.897)  PSP vs MSA-C – yes (AUC 0.954) |
| Mostile et al 2016^241^ | PSP | VaP | PSP vs VaP - yes (AUC 0.993) |
| Zanigni et al 2016^242^ | PSP-RS | PD | PSP-RS vs PD - yes (accuracy 99%) |
| Pasha et al 2016^243^ | PSP-RS  PSP-P | HC | PSP-RS / PSP-P vs HC - yes  PSP-RS vs PSP-P - yes |
| Sankhla et al 2016^244^ | PSP | PD  HC | PSP vs PD - yes (accuracy 98.21%)  PSP vs HC - yes (accuracy 100%) |
| Tipton et al 2016^245^ | PSP | CBS  MS  DLB | PSP vs CBD / MS / DLB (cerebral peduncle angle only) - no |
| Hwang et al 2016^246^ | PSP-RS  PSP-P | PD | Early (<3y)  PSP-RS vs PD / PSP-P - yes  PSP-P vs PD - no  Late (>3y)  PSP-RS vs PD / PSP-P - yes  PSP-P vs PD - yes |
| Owens et al 2016^247^ | PSP | PD  MSA | PSP vs MSA - yes  PSP vs PD - yes  *Predated PSP diagnosis by at least a month in 82% of PSP group* |
| Fatterpekar et al 2015^248^ | PSP-RS | HC  MSA  PD | PSP vs HC / MSA / PD (cerebral peduncle angle only) - yes (AUC 0.795) |
| Kaasinen et al 2015^249^ | PSP (definite) | Non-PSP:  PD  MSA  DLB  CBD | PSP vs non-PSP - yes |
| Whitwell et al 2013^250^ | PSP (definite)  *PSP-other (not diagnosed PSPS)*  *PSP-PSPS (PSPS + pathology)* | Disease controls  HC  CBD  CBD-PSPS | PSP-PSPS vs other group - yes  PSP-other vs other groups – no  All PSPS (PSP-PSPS, CBD-PSPS) vs no PSPS (CBD, PSP-other) – yes (AUC 0.99) |
| Massey et al 2013^251^ | PSP | HC  PD  MSA | PSP vs HC - yes  PSP vs MSA – yes (AUC 0.95)  PSP vs PD - yes |
| Massey et al 2012^252^ | PSP | HC  MSA  PD  CBD | PSP vs HC - yes  PSP vs MSA – yes (accuracy 86%) |
| Whitwell et al 2012^253^ | PSP | HC | PSP vs HC - yes |
| Morelli et al 2011^254^ | PSP | HC  PD | PSP vs HC – yes (accuracy 100%)  PSP vs PD - yes (accuracy 99.5%) |
| Looi et al 2011^255^ | PSP | HC | PSP vs HC - yes |
| Choi et al 2011^256^ | PSP | VaP  PD | PSP vs PD - yes  PSP vs VaP - yes |
| Gama et al 2010^257^ | PSP | PD  MSA-C  MSA-P | PSP vs PD / MSA-C / MSA-P – yes  (accuracy 96.7%) |
| Longoni et al 2010^258^ | PSP-RS  PSP-P | PD  HC | PSP vs PD - yes (accuracy 97%)  PSP-P vs PD - yes (accuracy 86%) |
| Emam et al 2010^259^ | PSP | PD  MSA-P  HC | PSP vs PD / MSA-P / HC - yes |
| Hussl et al 2010^260^ | PSP | PD  MSA-P | PSP vs PD - yes (accuracy 87%)  PSP vs MSA-P - yes (accuracy 87.5%) |
| Rohrer et al 2010^261^ | PSP-RS  PSP-PNFA | HC  PNFA | PSP-RS vs HC/ PNFA / PSP-PNFA – yes  PSP-PNFA vs HC / PNFA - yes |
| Sung et al 2009^262^ | PSP | SIVD | PSP vs SIVD - no |
| Slowinski et al 2008^263^ | PSP (definite) | HC | PSP vs HC – yes |
| Quattrone et al 2008^264^ | PSP (16 possible, 17 probable) | PD  MSA-P  HC | PSP vs HC - yes  PSP vs PD - yes  PSP vs MSA-P - yes |
| Cosottini et al 2007^265^ | PSP | MSA-P  HC | PSP vs HC / MSA-P – yes (AUC 0.99) |
| Barsottini et al 2007^266^ | PSP | PD | PSP vs PD - yes |
| Groschel et al 2006^267^ | PSP | CBD  HC | PSP vs HC / CBD - yes |
| Strecker et al 2006^268^ | PSP | WD  HC | PSP vs HC - yes  PSP vs WD - no |
| Righini et al 2004^269^ | PSP | PD | PSP vs PD - yes |

PSP: Progressive supranuclear palsy

PSPS: Progressive supranuclear palsy syndrome

PSP-RS: Progressive supranuclear palsy – Richardson’s Syndrome

PSP-P: Progressive supranuclear palsy – Parkinsonism variant

PSP-PAGF/PGF: Progressive supranuclear palsy – Pure Akinesia with Gait Freezing variant

PSP-CBS: Progressive supranuclear palsy – Corticobasal Syndrome variant

PSP-F: Progressive supranuclear palsy –Frontal variant

PSP-SL: Progressive supranuclear palsy – Speech/Language variant

PSP-corticospinal: Progressive supranuclear palsy – with prominent corticospinal signs

vPSP: variant Progressive Supranuclear Palsy

iNPH: idiopathic Normal Pressure Hydrocephalus

DLB: Dementia with Lewy Bodies

CBS: Corticobasal Syndrome

CBD: Corticobasal Degeneration

HC: Healthy Controls

PD: Parkinson’s Disease

MSA: Multiple System Atrophy

MSA-P: Multiple System Atrophy – Parkinsonism variant

MSA-C: Multiple System Atrophy – Cerebellar variant

FTLD: Frontotemporal Lobar Degeneration

VaP: Vascular Parkinsonism

AD: Alzheimer’s Disease

PNFA: Progressive Non-Fluent Aphasia

LPA: Logopenic/Phonological Aphasia

MS: Multiple Sclerosis

SIVD: subcortical ischaemic vascular dementia

WD: Wilson’s Disease

**Supplementary table 3:** fMRI studies in PSP

| Study | Approach | Main Findings |
| --- | --- | --- |
| Aghakhanyan et al 2021^23^ | Resting state/task-free | Hypoactivation between cerebellum and brainstem/globus pallidus, and limbic regions  Hyperactivation between cerebellum and cortical associative node |
| Cope et al 2017^61^ | Resting state/task-free | Increasing tau burden in midbrain and deep nuclei, and disrupted cortico-brainstem and cortico-subcortical interactions was associated with strengthening of cortico-cortical connectivity |
| Rosskopf et al 2017^72^ | Resting state/task-free | Increased functional connectivity between midbrain and thalamus  Correlation between vertical gaze impairment and lower midbrain connectivity |
| Brown et al 2017^89^ | Resting state/task-free | Functional connectivity deficits includ subcortical-brainstem modules on analysis of rostral midbrain tegmentum ICN |
| Upadhyay et al 2017^270^ | Resting state/task-free | Reduced functional connectivity between dentate nucleus and basal ganglia, thalamus, prefrontal cortex |
| Yu et al 2018^68^ | Task-based meta-analytical connectivity modelling | Co-activation of multiple seeds (regions selected based on voxel based morphometry studies) with cerebellum |
| Burciu et al 2016^103^ | Task-based | Decreased functional activity in all regions-of-interest along cerebello-thalamo-cortical network, including ipsilateral cerebellum |
| Burciu et al 2015^112^ | Task-based | Co-occuring regions of reduced and increased cerebellar connectivity in analysis of cerebello-thalamo-cortical and basal ganglia-thalamo-cortical networks |
| Piattella et al 2015^105^ | Resting state/task-free | Reduced connectivity between caudate, putamen, pallidum and posterior cerebellum |
| Gardner et al 2013^126^ | Resting state/task-free | Connectivity reductions throughout dorsal midbrain tegmentum ICN, including prominent decrease in cerebellum |
| Whitwell et al 2011^149^ | Resting state/task-free | Reduced thalamic connectivity with cerebellum (among other regions)  No connectivity changes involving cerebellum on analysis of the basal ganglia, salience or default mode networks |
| Zwergel et al 2011^271^ | Imagined standing | Higher fall frequency associated with decreased BOLD signal in the midbrain during imagined standing |

BOLD: Blood-Oxygen-Level-Dependent signal, ICN: Intrinsic Connectivity Network

References

1. Chatterjee K, Paul S, Banerjee R, et al. Characterizing gait and exploring neuro-morphometry in patients with PSP-Richardson’s syndrome and vascular parkinsonism. *Park Relat Disord*. 2023;113:105483. doi:10.1016/j.parkreldis.2023.105483

2. Ota M., Sato N., Takahashi Y., et al. Correlation between the regional brain volume and glymphatic system activity in progressive supranuclear palsy. *Dement Geriatr Cogn Disord*. Published online 2023. doi:10.1159/000530075

3. Nobileau A, Gaurav R, Chougar L, et al. Neuromelanin-Sensitive Magnetic Resonance Imaging Changes in the Locus Coeruleus/Subcoeruleus Complex in Patients with Typical and Atypical Parkinsonism. *Mov Disord*. 2023;38(3):479-484. doi:10.1002/mds.29309

4. Oliveira Hauer K., Pawlik D., Leuzy A., et al. Performance of [18F]RO948 PET, MRI and CSF neurofilament light in the differential diagnosis of progressive supranuclear palsy. *Parkinsonism Relat Disord*. 2023;106:105226. doi:10.1016/j.parkreldis.2022.11.018

5. Matsuoka K., Takado Y., Tagai K., et al. Two pathways differentially linking tau depositions, oxidative stress, and neuronal loss to apathetic phenotypes in progressive supranuclear palsy. *J Neurol Sci*. 2023;444:120514. doi:10.1016/j.jns.2022.120514

6. Welter M.-L., Vasseur A., Edragas R., et al. Brain dysfunction in gait disorders of Caribbean atypical Parkinsonism and progressive supranuclear palsy patients: A comparative study. *NeuroImage Clin*. 2023;38:103443. doi:10.1016/j.nicl.2023.103443

7. Painous C., Pascual-Diaz S., Munoz-Moreno E., et al. Midbrain and pons MRI shape analysis and its clinical and CSF correlates in degenerative parkinsonisms: a pilot study. *Eur Radiol*. Published online 2023. doi:10.1007/s00330-023-09435-0

8. Chougar L, Lejeune FX, Faouzi J, et al. Comparison of mean diffusivity, R2* relaxation rate and morphometric biomarkers for the clinical differentiation of parkinsonism. *Parkinsonism Relat Disord*. 2023;108(9513583):105287. doi:10.1016/j.parkreldis.2023.105287

9. Gatto R.G., Martin P.R., Ali F., et al. Diffusion tractography of superior cerebellar peduncle and dentatorubrothalamic tracts in two autopsy confirmed progressive supranuclear palsy variants: Richardson syndrome and the speech-language variant. *NeuroImage Clin*. 2022;35:103030. doi:10.1016/j.nicl.2022.103030

10. Tomse P., Rebec E., Studen A., et al. Abnormal metabolic covariance patterns associated with multiple system atrophy and progressive supranuclear palsy. *Phys Med*. 2022;98:131-138. doi:10.1016/j.ejmp.2022.04.016

11. Kanel P., Spears C.C., Roytman S., et al. Differential cholinergic systems’ changes in progressive supranuclear palsy versus Parkinson’s disease: an exploratory analysis. *J Neural Transm*. 2022;129(12):1469-1479. doi:10.1007/s00702-022-02547-9

12. Whitwell J.L., Tosakulwong N., Clark H.M., et al. Diffusion tensor imaging analysis in three progressive supranuclear palsy variants. *J Neurol*. 2021;268(9):3409-3420. doi:10.1007/s00415-020-10360-1

13. Tessema AW, Lee H, Gong Y, et al. Automated volumetric determination of high R(2) (*) regions in substantia nigra: A feasibility study of quantifying substantia nigra atrophy in progressive supranuclear palsy. *NMR Biomed*. 2022;35(11):e4795. doi:10.1002/nbm.4795

14. Buciuc M., Koga S., Pham N.T.T., et al. The many faces of globular glial tauopathy: A clinical and imaging study. *Eur J Neurol*. 2023;30(2):321-333. doi:10.1111/ene.15603

15. Hosapatna M., Verma A., D’souza A.S., Prasanna L.C. The role of width of pars compacta of substantia nigra and the midbrain area in patients with Parkinson’s disease and progressive supranuclear palsy with healthy aged individuals. *Biomed India*. 2022;42(4):666-670. doi:10.51248/.v42i4.1180

16. Mazzucchi S., Del Prete E., Costagli M., et al. Morphometric imaging and quantitative susceptibility mapping as complementary tools in the diagnosis of parkinsonisms. *Eur J Neurol*. 2022;29(10):2944-2955. doi:10.1111/ene.15447

17. Chougar L, Arsovic E, Gaurav R, et al. Regional Selectivity of Neuromelanin Changes in the Substantia Nigra in Atypical Parkinsonism. *Mov Disord*. 2022;37(6):1245-1255. doi:10.1002/mds.28988

18. Wang M.-L., Sun Z., Li W.-B., et al. Enlarged perivascular spaces and white matter hyperintensities in patients with frontotemporal lobar degeneration syndromes. *Front Aging Neurosci*. 2022;14:923193. doi:10.3389/fnagi.2022.923193

19. Zhang P., Chen J., Cai T., et al. Quantitative susceptibility mapping and blood neurofilament light chain differentiate between parkinsonian disorders. *Front Aging Neurosci*. 2022;14:909552. doi:10.3389/fnagi.2022.909552

20. Alster P., Nieciecki M., Migda B., et al. The Strengths and Obstacles in the Differential Diagnosis of Progressive Supranuclear Palsy-Parkinsonism Predominant (PSP-P) and Multiple System Atrophy (MSA) Using Magnetic Resonance Imaging (MRI) and Perfusion Single Photon Emission Computed Tomography. *Diagnostics*. 2022;12(2):385. doi:10.3390/diagnostics12020385

21. Ye R, O’Callaghan C, Rua C, et al. Locus Coeruleus Integrity from 7 T MRI Relates to Apathy and Cognition in Parkinsonian Disorders. *Mov Disord*. 2022;37(8):1663-1672. doi:10.1002/mds.29072

22. Zhao Y., Wu P., Wu J., et al. Decoding the dopamine transporter imaging for the differential diagnosis of parkinsonism using deep learning. *Eur J Nucl Med Mol Imaging*. 2022;49(8):2798-2811. doi:10.1007/s00259-022-05804-x

23. Aghakhanyan G., Rullmann M., Rumpf J., et al. Interplay of tau and functional network connectivity in progressive supranuclear palsy: a [18F]PI-2620 PET/MRI study. *Eur J Nucl Med Mol Imaging*. 2022;50(1):103-114. doi:10.1007/s00259-022-05952-0

24. Choi J.-H., Kim H., Shin J.H., et al. Eye movements and association with regional brain atrophy in clinical subtypes of progressive supranuclear palsy. *J Neurol*. 2021;268(3):967-977. doi:10.1007/s00415-020-10230-w

25. Woo K.A., Shin J.Y., Kim H., Ahn J., Jeon B., Lee J.-Y. Peripapillary retinal nerve fiber layer thinning in patients with progressive supranuclear palsy. *J Neurol*. 2022;269(6):3216-3225. doi:10.1007/s00415-021-10936-5

26. Lee H, Lee MJ, Kim EJ, Huh GY, Lee JH, Cho H. Iron accumulation in the oculomotor nerve of the progressive supranuclear palsy brain. *Sci Rep*. 2021;11(1):2950. doi:10.1038/s41598-021-82469-w

27. Buch K.A., Bouffard M.A., Kardon R.H., et al. Clinical Correlation Between Vertical Gaze Palsy and Midbrain Volume in Progressive Supranuclear Palsy. *J Neuro-Ophthalmol Off J North Am Neuro-Ophthalmol Soc*. 2022;42(2):246-250. doi:10.1097/WNO.0000000000001393

28. Matsuura K, Ii Y, Maeda M, et al. Neuromelanin-sensitive magnetic resonance imaging in disease differentiation for parkinsonism or neurodegenerative disease affecting the basal ganglia. *Park Relat Disord*. 2021;87:75-81. doi:10.1016/j.parkreldis.2021.05.002

29. Li L., Liu F.-T., Li M., et al. Clinical Utility of 18F-APN-1607 Tau PET Imaging in Patients with Progressive Supranuclear Palsy. *Mov Disord*. 2021;36(10):2314-2323. doi:10.1002/mds.28672

30. Marti-Andres G., van Bommel L., Meles S.K., et al. Multicenter Validation of Metabolic Abnormalities Related to PSP According to the MDS-PSP Criteria. *Mov Disord*. 2020;35(11):2009-2018. doi:10.1002/mds.28217

31. Whitwell J.L., Tosakulwong N., Botha H., et al. Brain volume and flortaucipir analysis of progressive supranuclear palsy clinical variants. *NeuroImage Clin*. 2020;25:102152. doi:10.1016/j.nicl.2019.102152

32. Holland N., Jones P.S., Savulich G., et al. Synaptic Loss in Primary Tauopathies Revealed by [11C]UCB-J Positron Emission Tomography. *Mov Disord*. 2020;35(10):1834-1842. doi:10.1002/mds.28188

33. Palmisano C., Todisco M., Marotta G., et al. Gait initiation in progressive supranuclear palsy: brain metabolic correlates. *NeuroImage Clin*. 2020;28:102408. doi:10.1016/j.nicl.2020.102408

34. Tse NY, Chen Y, Irish M, et al. Cerebellar contributions to cognition in corticobasal syndrome and progressive supranuclear palsy. *Brain Commun*. 2020;2(2):fcaa194. doi:10.1093/braincomms/fcaa194

35. Murakami N., Sako W., Haji S., et al. Differences in cerebellar perfusion between Parkinson’s disease and multiple system atrophy. *J Neurol Sci*. 2020;409:116627. doi:10.1016/j.jns.2019.116627

36. Ghirelli A, Tosakulwong N, Weigand SD, et al. Sensitivity-Specificity of Tau and Amyloid β Positron Emission Tomography in Frontotemporal Lobar Degeneration. *Ann Neurol*. 2020;88(5):1009-1022. doi:10.1002/ana.25893

37. Anagnostou E., Karavasilis E., Potiri I., et al. A cortical substrate for square-wave jerks in progressive supranuclear palsy. *J Clin Neurol Korea*. 2020;16(1):37-45. doi:10.3988/jcn.2020.16.1.37

38. Brendel M., Barthel H., Van Eimeren T., et al. Assessment of 18F-PI-2620 as a Biomarker in Progressive Supranuclear Palsy. *JAMA Neurol*. 2020;77(11):1408-1419. doi:10.1001/jamaneurol.2020.2526

39. Schroter N., Blazhenets G., Frings L., et al. Tau Imaging in the 4-Repeat-Tauopathies Progressive Supranuclear Palsy and Corticobasal Syndrome: A 11C-Pyridinyl-Butadienyl-Benzothiazole 3 PET Pilot Study. *Clin Nucl Med*. 2020;45(4):283-287. doi:10.1097/RLU.0000000000002949

40. Potrusil T., Krismer F., Beliveau V., et al. Diagnostic potential of automated tractography in progressive supranuclear palsy variants. *Parkinsonism Relat Disord*. 2020;72:65-71. doi:10.1016/j.parkreldis.2020.02.007

41. Abos A., Segura B., Baggio H.C., et al. Disrupted structural connectivity of fronto-deep gray matter pathways in progressive supranuclear palsy. *NeuroImage Clin*. 2019;23:101899. doi:10.1016/j.nicl.2019.101899

42. Mazzucchi S, Frosini D, Costagli M, et al. Quantitative susceptibility mapping in atypical Parkinsonisms. *Neuroimage Clin*. 2019;24:101999. doi:10.1016/j.nicl.2019.101999

43. Whitwell J.L., Stevens C.A., Duffy J.R., et al. An Evaluation of the Progressive Supranuclear Palsy Speech/Language Variant. *Mov Disord Clin Pract*. 2019;6(6):452-461. doi:10.1002/mdc3.12796

44. Nicastro N., Rodriguez P.V., Malpetti M., et al. 18F-AV1451 PET imaging and multimodal MRI changes in progressive supranuclear palsy. *J Neurol*. 2020;267(2):341-349. doi:10.1007/s00415-019-09566-9

45. Sintini I., Schwarz C.G., Senjem M.L., et al. Multimodal neuroimaging relationships in progressive supranuclear palsy. *Parkinsonism Relat Disord*. 2019;66:56-61. doi:10.1016/j.parkreldis.2019.07.001

46. Ghourchian S., Mousavi A., Zamani B., Shahidi G., Rohani M. Midbrain area for differentiating Parkinson’s disease from progressive supranuclear palsy. *Clin Neurol Neurosurg*. 2019;183:105383. doi:10.1016/j.clineuro.2019.105383

47. Ramanan S, Strikwerda-Brown C, Mothakunnel A, Hodges JR, Piguet O, Irish M. Fronto-parietal contributions to episodic retrieval-evidence from neurodegenerative disorders. *Learn Mem Cold Spring Harb N*. 2019;26(7):262-271. doi:10.1101/lm.048454.118

48. Pyatigorskaya N., Yahia-Cherif L., Gaurav R., et al. Multimodal Magnetic Resonance Imaging Quantification of Brain Changes in Progressive Supranuclear Palsy. *Mov Disord*. 2020;35(1):161-170. doi:10.1002/mds.27877

49. Sjostrom H., Surova Y., Nilsson M., et al. Mapping of apparent susceptibility yields promising diagnostic separation of progressive supranuclear palsy from other causes of parkinsonism. *Sci Rep*. 2019;9(1):6079. doi:10.1038/s41598-019-42565-4

50. Schonecker S., Brendel M., Palleis C., et al. PET Imaging of Astrogliosis and Tau Facilitates Diagnosis of Parkinsonian Syndromes. *Front Aging Neurosci*. 2019;11:249. doi:10.3389/fnagi.2019.00249

51. Mitchell T., Archer D.B., Chu W.T., et al. Neurite orientation dispersion and density imaging (NODDI) and free-water imaging in Parkinsonism. *Hum Brain Mapp*. 2019;40(17):5094-5107. doi:10.1002/hbm.24760

52. Endo H., Shimada H., Sahara N., et al. In vivo binding of a tau imaging probe, [11C]PBB3, in patients with progressive supranuclear palsy. *Mov Disord*. 2019;34(5):744-754. doi:10.1002/mds.27643

53. Spotorno N., Hall S., Irwin D.J., et al. Diffusion tensor MRI to distinguish progressive supranuclear palsy from a-synucleinopathies. *Radiology*. 2019;293(3):646-653. doi:10.1148/radiol.2019190406

54. Sjostrom H, Granberg T, Hashim F, Westman E, Svenningsson P. Automated brainstem volumetry can aid in the diagnostics of parkinsonian disorders. *Parkinsonism Relat Disord*. 2020;79(9513583):18-25. doi:10.1016/j.parkreldis.2020.08.004

55. Nigro S., Barbagallo G., Bianco M.G., et al. Track density imaging: A reliable method to assess white matter changes in Progressive Supranuclear Palsy with predominant parkinsonism. *Parkinsonism Relat Disord*. 2019;69((Nigro) Department of Experimental and Clinical Medicine, Magna Graecia University, Catanzaro, Italy(Barbagallo, Morelli, Arabia, Quattrone, Gasparini) Department of Medical and Surgical Sciences, Institute of Neurology, Magna Graecia University, Catanzar):23-29. doi:10.1016/j.parkreldis.2019.10.020

56. Alonso-Canovas A, Tembl Ferrairó JI, Martínez-Torres I, et al. Transcranial sonography in atypical parkinsonism: How reliable is it in real clinical practice? A multicentre comprehensive study. *Park Relat Disord*. 2019;68:40-45. doi:10.1016/j.parkreldis.2019.09.032

57. Azuma M, Hirai T, Nakaura T, et al. Combining quantitative susceptibility mapping to the morphometric index in differentiating between progressive supranuclear palsy and Parkinson’s disease. *J Neurol Sci*. 2019;406:116443. doi:10.1016/j.jns.2019.116443

58. Alster P., Nieciecki M., Koziorowski D.M., et al. Thalamic and cerebellar hypoperfusion in single photon emission computed tomography may differentiate multiple system atrophy and progressive supranuclear palsy. *Medicine (Baltimore)*. 2019;98(30):e16603. doi:10.1097/MD.0000000000016603

59. Caligiuri M.E., Morelli M., Nigro S., et al. Imaging counterpart of postural instability and vertical ocular dysfunction in patients with PSP: A multimodal MRI study. *Parkinsonism Relat Disord*. 2019;63:124-130. doi:10.1016/j.parkreldis.2019.02.022

60. Albrecht F, Bisenius S, Neumann J, Whitwell J, Schroeter ML. Atrophy in midbrain & cerebral/cerebellar pedunculi is characteristic for progressive supranuclear palsy - A double-validation whole-brain meta-analysis. *NeuroImage Clin*. 2019;22:101722. doi:10.1016/j.nicl.2019.101722

61. Cope T.E., Rittman T., Borchert R.J., et al. Tau burden and the functional connectome in Alzheimer’s disease and progressive supranuclear palsy. *Brain*. 2018;141(2):550-567. doi:10.1093/brain/awx347

62. Passamonti L., Rodriguez P.V., Hong Y.T., et al. PK11195 binding in Alzheimer disease and progressive supranuclear palsy. *Neurology*. 2018;90(22):e1989-e1996. doi:10.1212/WNL.0000000000005610

63. Taniguchi D., Hatano T., Kamagata K., et al. Neuromelanin imaging and midbrain volumetry in progressive supranuclear palsy and Parkinson’s disease. *Mov Disord*. 2018;33(9):1488-1492. doi:10.1002/mds.27365

64. Constantinides V.C., Paraskevas G.P., Stamboulis E., Kapaki E. Simple linear brainstem MRI measurements in the differential diagnosis of progressive supranuclear palsy from the parkinsonian variant of multiple system atrophy. *Neurol Sci*. 2018;39(2):359-364. doi:10.1007/s10072-017-3212-2

65. Lipp A., Skowronek C., Fehlner A., Streitberger K.-J., Braun J., Sack I. Progressive supranuclear palsy and idiopathic Parkinson’s disease are associated with local reduction of in vivo brain viscoelasticity. *Eur Radiol*. 2018;28(8):3347-3354. doi:10.1007/s00330-017-5269-y

66. Seki M., Seppi K., Mueller C., et al. Diagnostic potential of dentatorubrothalamic tract analysis in progressive supranuclear palsy. *Parkinsonism Relat Disord*. 2018;49:81-87. doi:10.1016/j.parkreldis.2018.02.004

67. Whitwell JL, Tosakulwong N, Schwarz CG, et al. MRI Outperforms [18F]AV-1451 PET as a Longitudinal Biomarker in Progressive Supranuclear Palsy. *Mov Disord Off J Mov Disord Soc*. 2019;34(1):105-113. doi:10.1002/mds.27546

68. Yu F., Barron D.S., Tantiwongkosi B., Fox M., Fox P. Characterisation of meta-analytical functional connectivity in progressive supranuclear palsy. *Clin Radiol*. 2018;73(4):e1-415. doi:10.1016/j.crad.2017.11.007

69. Coakeley S., Cho S.S., Koshimori Y., et al. [18F]AV-1451 binding to neuromelanin in the substantia nigra in PD and PSP. *Brain Struct Funct*. 2018;223(2):589-595. doi:10.1007/s00429-017-1507-y

70. Kamada T., Miura S., Kida H., et al. MIBG myocardial scintigraphy in progressive supranuclear palsy. *J Neurol Sci*. 2019;396((Kamada, Miura, Kida, Irie, Hoshino, Taniwaki) Division of Respirology, Neurology and Rheumatology, Department of Medicine, Kurume University School of Medicine, 67 Asashi-machi, Kurume 830-0011, Japan(Yamanishi) Department of Bioscience and Bioinformatic):3-7. doi:10.1016/j.jns.2018.10.019

71. Talai A.S., Sedlacik J., Boelmans K., Forkert N.D. Widespread diffusion changes differentiate Parkinson’s disease and progressive supranuclear palsy. *NeuroImage Clin*. 2018;20:1037-1043. doi:10.1016/j.nicl.2018.09.028

72. Rosskopf J., Gorges M., Muller H.-P., et al. Intrinsic functional connectivity alterations in progressive supranuclear palsy: Differential effects in frontal cortex, motor, and midbrain networks. *Mov Disord*. 2017;32(7):1006-1015. doi:10.1002/mds.27039

73. Gellersen HM, Guo CC, O’Callaghan C, Tan RH, Sami S, Hornberger M. Cerebellar atrophy in neurodegeneration—a meta-analysis. *J Neurol Neurosurg Psychiatry*. 2017;88(9):780-788. doi:10.1136/jnnp-2017-315607

74. Passamonti L., Rodriguez P.V., Hong Y.T., et al. 18F-AV-1451 positron emission tomography in Alzheimer’s disease and progressive supranuclear palsy. *Brain*. 2017;140(3):781-791. doi:10.1093/brain/aww340

75. Zanigni S., Evangelisti S., Testa C., et al. White matter and cortical changes in atypical parkinsonisms: A multimodal quantitative MR study. *Parkinsonism Relat Disord*. 2017;39:44-51. doi:10.1016/j.parkreldis.2017.03.001

76. Gorges M., Maier M.N., Rosskopf J., et al. Regional microstructural damage and patterns of eye movement impairment: a DTI and video-oculography study in neurodegenerative parkinsonian syndromes. *J Neurol*. 2017;264(9):1919-1928. doi:10.1007/s00415-017-8579-8

77. Brendel M., Schonecker S., Hoglinger G., et al. [18F]-THK5351 PET correlates with topology and symptom severity in progressive supranuclear palsy. *Front Aging Neurosci*. 2018;9(JAN):440. doi:10.3389/fnagi.2017.00440

78. Ge J., Wu J., Peng S., et al. Reproducible network and regional topographies of abnormal glucose metabolism associated with progressive supranuclear palsy: Multivariate and univariate analyses in American and Chinese patient cohorts. *Hum Brain Mapp*. 2018;39(7):2842-2858. doi:10.1002/hbm.24044

79. Sakurai K., Imabayashi E., Tokumaru A.M., et al. Volume of Interest Analysis of Spatially Normalized PRESTO Imaging to Differentiate between Parkinson Disease and Atypical Parkinsonian Syndrome. *Magn Reson Med Sci MRMS Off J Jpn Soc Magn Reson Med*. 2017;16(1):16-22. doi:10.2463/mrms.mp.2015-0132

80. Mueller K., Jech R., Bonnet C., et al. Disease-specific regions outperform whole-brain approaches in identifying progressive supranuclear palsy: A multicentric MRI study. *Front Neurosci*. 2017;11(MAR):100. doi:10.3389/fnins.2017.00100

81. Whitwell J.L., Lowe V.J., Tosakulwong N., et al. [18F]AV-1451 tau positron emission tomography in progressive supranuclear palsy. *Mov Disord*. 2017;32(1):124-133. doi:10.1002/mds.26834

82. Agosta F., Caso F., Jecmenica-Lukic M., et al. Tracking brain damage in progressive supranuclear palsy: A longitudinal MRI study. *J Neurol Neurosurg Psychiatry*. 2018;89(7):696-701. doi:10.1136/jnnp-2017-317443

83. Lee Y., Lee D.K., Lee J.M., et al. Volumetric analysis of the cerebellum in patients with progressive supranuclear palsy. *Eur J Neurol*. 2017;24(1):212-218. doi:10.1111/ene.13185

84. Lee S.H., Lyoo C.H., Ahn S.J., Rinne J.O., Lee M.S. Brain regional iron contents in progressive supranuclear palsy. *Parkinsonism Relat Disord*. 2017;45:28-32. doi:10.1016/j.parkreldis.2017.09.020

85. Pan P., Liu Y., Zhang Y., Zhao H., Ye X., Xu Y. Brain gray matter abnormalities in progressive supranuclear palsy revisited. *Oncotarget*. 2017;8(46):80941-80955. doi:10.18632/oncotarget.20895

86. Ofori E, Krismer F, Burciu RG, et al. Free water improves detection of changes in the substantia nigra in parkinsonism: A multisite study. *Mov Disord*. 2017;32(10):1457-1464. doi:10.1002/mds.27100

87. Sako W., Murakami N., Izumi Y., Kaji R. Usefulness of the superior cerebellar peduncle for differential diagnosis of progressive supranuclear palsy: A meta-analysis. *J Neurol Sci*. 2017;378:153-157. doi:10.1016/j.jns.2017.05.005

88. Sugiyama A, Sato N, Kimura Y, et al. MR findings in the substantia nigra on phase difference enhanced imaging in neurodegenerative parkinsonism. *Park Relat Disord*. 2018;48:10-16. doi:10.1016/j.parkreldis.2017.12.021

89. Brown J.A., Hua A.Y., Trujllo A., et al. Advancing functional dysconnectivity and atrophy in progressive supranuclear palsy. *NeuroImage Clin*. 2017;16((Brown, Hua, Trujllo, Attygalle, Spina, Lee, Kramer, Miller, Rosen, Boxer, Seeley) Memory and Aging Center, Department of Neurology, University of California San Francisco, San Francisco, CA, United States(Binney) Temple University, Eleanor M. Saffran Cen):564-574. doi:10.1016/j.nicl.2017.09.008

90. Schonhaut D.R., McMillan C.T., Spina S., et al. 18F-flortaucipir tau positron emission tomography distinguishes established progressive supranuclear palsy from controls and Parkinson disease: A multicenter study. *Ann Neurol*. 2017;82(4):622-634. doi:10.1002/ana.25060

91. Ito K., Ohtsuka C., Yoshioka K., et al. Differential diagnosis of parkinsonism by a combined use of diffusion kurtosis imaging and quantitative susceptibility mapping. *Neuroradiology*. 2017;59(8):759-769. doi:10.1007/s00234-017-1870-7

92. Nicoletti G., Caligiuri M.E., Cherubini A., et al. A fully automated, atlas-based approach for superior cerebellar peduncle evaluation in progressive supranuclear palsy phenotypes. *Am J Neuroradiol*. 2017;38(3):523-530. doi:10.3174/ajnr.A5048

93. Bologna M., Piattella M.C., Upadhyay N., et al. Neuroimaging correlates of blinking abnormalities in patients with progressive supranuclear palsy. *Mov Disord*. 2016;31(1):138-143. doi:10.1002/mds.26470

94. Huppertz H.-J., Moller L., Sudmeyer M., et al. Differentiation of neurodegenerative parkinsonian syndromes by volumetric magnetic resonance imaging analysis and support vector machine classification. *Mov Disord*. 2016;31(10):1506-1517. doi:10.1002/mds.26715

95. Scherfler C., Gobel G., Muller C., et al. Diagnostic potential of automated subcortical volume segmentation in atypical parkinsonism. *Neurology*. 2016;86(13):1242-1249. doi:10.1212/WNL.0000000000002518

96. Upadhyay N., Suppa A., Piattella M.C., et al. MRI gray and white matter measures in progressive supranuclear palsy and corticobasal syndrome. *J Neurol*. 2016;263(10):2022-2031. doi:10.1007/s00415-016-8224-y

97. Caso F., Agosta F., Volonte M.A., et al. Cognitive impairment in progressive supranuclear palsy-Richardson’s syndrome is related to white matter damage. *Parkinsonism Relat Disord*. 2016;31:65-71. doi:10.1016/j.parkreldis.2016.07.007

98. Cho H., Choi J.Y., Hwang M.S., et al. Subcortical 18F-AV-1451 binding patterns in progressive supranuclear palsy. *Mov Disord*. 2017;32(1):134-140. doi:10.1002/mds.26844

99. Coakeley S., Cho S.S., Koshimori Y., et al. Positron emission tomography imaging of tau pathology in progressive supranuclear palsy. *J Cereb Blood Flow Metab*. 2017;37(9):3150-3160. doi:10.1177/0271678X16683695

100. Zhang Y., Walter R., Ng P., et al. Progression of microstructural degeneration in progressive supranuclear palsy and corticobasal syndrome: A longitudinal diffusion tensor imaging study. *PLoS ONE*. 2016;11(6):e0157218. doi:10.1371/journal.pone.0157218

101. Planetta PJ, Ofori E, Pasternak O, et al. Free-water imaging in Parkinson’s disease and atypical parkinsonism. *Brain J Neurol*. 2016;139(Pt 2):495-508. doi:10.1093/brain/awv361

102. Dutt S., Binney R.J., Heuer H.W., et al. Progression of brain atrophy in PSP and CBS over 6 months and 1 year. *Neurology*. 2016;87(19):2016-2025. doi:10.1212/WNL.0000000000003305

103. Burciu R.G., Chung J.W., Shukla P., et al. Functional MRI of disease progression in Parkinson disease and atypical parkinsonian syndromes. *Neurology*. 2016;87(7):709-717. doi:10.1212/WNL.0000000000002985

104. Piattella M.C., Upadhyay N., Bologna M., et al. Neuroimaging evidence of gray and white matter damage and clinical correlates in progressive supranuclear palsy. *J Neurol*. 2015;262(8):1850-1858. doi:10.1007/s00415-015-7779-3

105. Piattella M.C., Tona F., Bologna M., et al. Disrupted Resting-State Functional Connectivity in Progressive Supranuclear Palsy. *Am J Neuroradiol*. 2015;36(5):915-921. doi:10.3174/AJNR.A4229

106. Wang G., Wang J., Zhan J., et al. Quantitative assessment of cerebral gray matter density change in progressive supranuclear palsy using voxel based morphometry analysis and cerebral MR T1-weighted FLAIR imaging. *J Neurol Sci*. 2015;359(1-2):367-372. doi:10.1016/j.jns.2015.11.007

107. Reiter E, Mueller C, Pinter B, et al. Dorsolateral nigral hyperintensity on 3.0T susceptibility-weighted imaging in neurodegenerative Parkinsonism. *Mov Disord*. 2015;30(8):1068-1076. doi:10.1002/mds.26171

108. Fukui Y., Hishikawa N., Sato K., et al. Differentiating progressive supranuclear palsy from Parkinson’s disease by MRI-based dynamic cerebrospinal fluid flow. *J Neurol Sci*. 2015;357(1-2):178-182. doi:10.1016/j.jns.2015.07.026

109. Meijer F.J.A., van Rumund A., Tuladhar A.M., et al. Conventional 3T brain MRI and diffusion tensor imaging in the diagnostic workup of early stage parkinsonism. *Neuroradiology*. 2015;57(7):655-669. doi:10.1007/s00234-015-1515-7

110. Zanigni S., Testa C., Calandra-Buonaura G., et al. The contribution of cerebellar proton magnetic resonance spectroscopy in the differential diagnosis among parkinsonian syndromes. *Parkinsonism Relat Disord*. 2015;21(8):929-937. doi:10.1016/j.parkreldis.2015.05.025

111. Surova Y., Nilsson M., Latt J., et al. Disease-specific structural changes in thalamus and dentatorubrothalamic tract in progressive supranuclear palsy. *Neuroradiology*. 2015;57(11):1079-1091. doi:10.1007/s00234-015-1563-z

112. Burciu R.G., Ofori E., Shukla P., et al. Distinct patterns of brain activity in progressive supranuclear palsy and Parkinson’s disease. *Mov Disord*. 2015;30(9):1248-1258. doi:10.1002/mds.26294

113. Ito K., Sasaki M., Ohtsuka C., et al. Differentiation among parkinsonisms using quantitative diffusion kurtosis imaging. *NeuroReport*. 2015;26(5):267-272. doi:10.1097/WNR.0000000000000341

114. Yu F., Barron D.S., Tantiwongkosi B., Fox P. Patterns of gray matter atrophy in atypical parkinsonism syndromes: A VBM meta-analysis. *Brain Behav*. 2015;5(6):1-10. doi:10.1002/brb3.329

115. Sadowski K, Serafin-Król M, Szlachta K, Friedman A. Basal ganglia echogenicity in tauopathies. *J Neural Transm Vienna*. 2015;122(6):863-865. doi:10.1007/s00702-014-1310-3

116. Worker A., Blain C., Jarosz J., et al. Diffusion tensor imaging of Parkinson’s disease, multiple system atrophy and progressive supranuclear palsy: A tract-based spatial statistics study. *PLoS ONE*. 2014;9(11):e112638. doi:10.1371/journal.pone.0112638

117. Ohtsuka C, Sasaki M, Konno K, et al. Differentiation of early-stage parkinsonisms using neuromelanin-sensitive magnetic resonance imaging. *Park Relat Disord*. 2014;20(7):755-760. doi:10.1016/j.parkreldis.2014.04.005

118. Tessitore A., Giordano A., Caiazzo G., et al. Clinical correlations of microstructural changes in progressive supranuclear palsy. *Neurobiol Aging*. 2014;35(10):2404-2410. doi:10.1016/j.neurobiolaging.2014.03.028

119. Shao N., Yang J., Li J., Shang H.-F. Voxelwise meta-analysis of gray matter anomalies in progressive supranuclear palsy and Parkinson’s disease using anatomic likelihood estimation. *Front Hum Neurosci*. 2014;8(1 FEB):63. doi:10.3389/fnhum.2014.00063

120. Hara K., Ito M., Tsuboi T., et al. Potential of a new MRI for visualizing cerebellar involvement in progressive supranuclear palsy. *Parkinsonism Relat Disord*. 2014;20(2):157-161. doi:10.1016/j.parkreldis.2013.10.007

121. Hughes L.E., Rowe J.B., Ghosh B.C., Carlyon R.P., Plack C.J., Gockel H.E. The binaural masking level difference: Cortical correlates persist despite severe brain stem atrophy in progressive supranuclear palsy. *J Neurophysiol*. 2014;112(12):3086-3094. doi:10.1152/jn.00062.2014

122. Whitwell J.L., Schwarz C.G., Reid R.I., Kantarci K., Jack Jr. C.R., Josephs K.A. Diffusion tensor imaging comparison of progressive supranuclear palsy and corticobasal syndromes. *Parkinsonism Relat Disord*. 2014;20(5):493-498. doi:10.1016/j.parkreldis.2014.01.023

123. Agosta F., Galantucci S., Svetel M., et al. Clinical, cognitive, and behavioural correlates of white matter damage in progressive supranuclear palsy. *J Neurol*. 2014;261(5):913-924. doi:10.1007/s00415-014-7301-3

124. Reginold W., Lang A.E., Marras C., Heyn C., Alharbi M., Mikulis D.J. Longitudinal quantitative MRI in multiple system atrophy and progressive supranuclear palsy. *Parkinsonism Relat Disord*. 2014;20(2):222-225. doi:10.1016/j.parkreldis.2013.10.002

125. Hutchinson M., Raff U., Chana P., Huete I. Spin-lattice distribution MRI maps nigral pathology in Progressive Supranuclear Palsy (PSP) during life: A pilot study. *PLoS ONE*. 2014;9(1):e85194. doi:10.1371/journal.pone.0085194

126. Gardner R.C., Boxer A.L., Trujillo A., et al. Intrinsic connectivity network disruption in progressive supranuclear palsy. *Ann Neurol*. 2013;73(5):603-616. doi:10.1002/ana.23844

127. Zwergal A., La Fougere C., Lorenzl S., et al. Functional disturbance of the locomotor network in progressive supranuclear palsy. *Neurology*. 2013;80(7):634-641. doi:10.1212/WNL.0b013e318281cc43

128. Salvatore C., Cerasa A., Castiglioni I., et al. Machine learning on brain MRI data for differential diagnosis of Parkinson’s disease and Progressive Supranuclear Palsy. *J Neurosci Methods*. 2014;222:230-237. doi:10.1016/j.jneumeth.2013.11.016

129. Marquand A.F., Filippone M., Ashburner J., et al. Automated, High Accuracy Classification of Parkinsonian Disorders: A Pattern Recognition Approach. *PLoS ONE*. 2013;8(7):e69237. doi:10.1371/journal.pone.0069237

130. Nicoletti G., Rizzo G., Barbagallo G., et al. Diffusivity of cerebellar hemispheres enables discrimination of cerebellar or parkinsonian multiple system atrophy from progressive supranuclear palsy-Richardson syndrome and Parkinson disease. *Radiology*. 2013;267(3):843-850. doi:10.1148/radiol.12120364

131. Botha H, Whitwell JL, Madhaven A, Senjem ML, Lowe V, Josephs KA. The pimple sign of progressive supranuclear palsy syndrome. *Parkinsonism Relat Disord*. 2014;20(2):180-185. doi:10.1016/j.parkreldis.2013.10.023

132. Kepe V., Bordelon Y., Boxer A., et al. PET imaging of neuropathology in tauopathies: Progressive supranuclear palsy. *J Alzheimers Dis*. 2013;36(1):145-153. doi:10.3233/JAD-130032

133. Josephs K.A., Xia R., Mandrekar J., et al. Modeling trajectories of regional volume loss in progressive supranuclear palsy. *Mov Disord*. 2013;28(8):1117-1124. doi:10.1002/mds.25437

134. Giordano A., Tessitore A., Corbo D., et al. Clinical and cognitive correlations of regional gray matter atrophy in progressive supranuclear palsy. *Parkinsonism Relat Disord*. 2013;19(6):590-594. doi:10.1016/j.parkreldis.2013.02.005

135. Sastre-Bataller I., Vazquez J.F., Martinez-Torres I., et al. Mesencephalic area measured by transcranial sonography in the differential diagnosis of parkinsonism. *Parkinsonism Relat Disord*. 2013;19(8):732-736. doi:10.1016/j.parkreldis.2013.04.010

136. Surova Y., Szczepankiewicz F., Latt J., et al. Assessment of Global and Regional Diffusion Changes along White Matter Tracts in Parkinsonian Disorders by MR Tractography. *PLoS ONE*. 2013;8(6):e66022. doi:10.1371/journal.pone.0066022

137. Coon E.A., Whitwell J.L., Jack C.R., Josephs K.A. Primary lateral sclerosis as progressive supranuclear palsy: Diagnosis by diffusion tensor imaging. *Mov Disord*. 2012;27(7):903-906. doi:10.1002/mds.24990

138. Jesse S, Kassubek J, Müller HP, Ludolph AC, Unrath A. Signal alterations of the basal ganglia in the differential diagnosis of Parkinson’s disease: a retrospective case-controlled MRI data bank analysis. *BMC Neurol*. 2012;12:163. doi:10.1186/1471-2377-12-163

139. Agosta F., Pievani M., Svetel M., et al. Diffusion tensor MRI contributes to differentiate Richardson’s syndrome from PSP-parkinsonism. *Neurobiol Aging*. 2012;33(12):2817-2826. doi:10.1016/j.neurobiolaging.2012.02.002

140. Saini J., Bagepally B.S., Sandhya M., et al. Subcortical structures in progressive supranuclear palsy: Vertex-based analysis. *Eur J Neurol*. 2013;20(3):493-501. doi:10.1111/j.1468-1331.2012.03884.x

141. Josephs K.A., Eggers S.D.Z., Jack C.R., Whitwell J.L. Neuroanatomical correlates of the progressive supranuclear palsy corticobasal syndrome hybrid. *Eur J Neurol*. 2012;19(11):1440-1446. doi:10.1111/j.1468-1331.2012.03726.x

142. Tsukamoto K., Matsusue E., Kanasaki Y., et al. Significance of apparent diffusion coefficient measurement for the differential diagnosis of multiple system atrophy, progressive supranuclear palsy, and Parkinson’s disease: Evaluation by 3.0-T MR imaging. *Neuroradiology*. 2012;54(9):947-955. doi:10.1007/s00234-012-1009-9

143. Saini J., Bagepally B.S., Sandhya M., Pasha S.A., Yadav R., Pal P.K. In vivo evaluation of white matter pathology in patients of progressive supranuclear palsy using TBSS. *Neuroradiology*. 2012;54(7):771-780. doi:10.1007/s00234-011-0983-7

144. Kimura N., Hanaki S., Masuda T., et al. Brain perfusion differences in Parkinsonian disorders. *Mov Disord*. 2011;26(14):2530-2537. doi:10.1002/mds.23915

145. Canu E., Agosta F., Baglio F., Galantucci S., Nemni R., Filippi M. Diffusion tensor magnetic resonance imaging tractography in progressive supranuclear palsy. *Mov Disord*. 2011;26(9):1752-1755. doi:10.1002/mds.23739

146. Rolland Y., Verin M., Payan C.A., et al. A new MRI rating scale for progressive supranuclear palsy and multiple system atrophy: Validity and reliability. *J Neurol Neurosurg Psychiatry*. 2011;82(9):1025-1032. doi:10.1136/jnnp.2010.214890

147. Kashihara K, Shinya T, Higaki F. Reduction of neuromelanin-positive nigral volume in patients with MSA, PSP and CBD. *Intern Med*. 2011;50(16):1683-1687. doi:10.2169/internalmedicine.50.5101

148. Whitwell J.L., Master A.V., Avula R., et al. Clinical correlates of white matter tract degeneration in progressive supranuclear palsy. *Arch Neurol*. 2011;68(6):753-760. doi:10.1001/archneurol.2011.107

149. Whitwell J.L., Avula R., Master A., et al. Disrupted thalamocortical connectivity in PSP: A resting-state fMRI, DTI, and VBM study. *Parkinsonism Relat Disord*. 2011;17(8):599-605. doi:10.1016/j.parkreldis.2011.05.013

150. Focke N.K., Helms G., Scheewe S., et al. Individual voxel-based subtype prediction can differentiate progressive supranuclear palsy from idiopathic Parkinson syndrome and healthy controls. *Hum Brain Mapp*. 2011;32(11):1905-1915. doi:10.1002/hbm.21161

151. Zhao P, Zhang B, Gao S. 18F-FDG PET study on the idiopathic Parkinson’s disease from several parkinsonian-plus syndromes. *Park Relat Disord*. 2012;18 Suppl 1:S60-2. doi:10.1016/S1353-8020(11)70020-7

152. Agosta F, Kostic VS, Galantucci S, et al. The in vivo distribution of brain tissue loss in Richardson’s syndrome and PSP-parkinsonism: a VBM-DARTEL study. *Eur J Neurosci*. 2010;32(4):640-647. doi:10.1111/j.1460-9568.2010.07304.x

153. Teune L.K., Bartels A.L., De Jong B.M., et al. Typical cerebral metabolic patterns in neurodegenerative brain diseases. *Mov Disord*. 2010;25(14):2395-2404. doi:10.1002/mds.23291

154. Fukui T., Lee E., Hosoda H., Okita K. Obsessive-compulsive behavior as a symptom of dementia in progressive supranuclear palsy. *Dement Geriatr Cogn Disord*. 2010;30(2):179-188. doi:10.1159/000310351

155. Knake S., Belke M., Menzler K., et al. In vivo demonstration of microstructural brain pathology in progressive supranuclear palsy: A DTI study using TBSS. *Mov Disord*. 2010;25(9):1232-1238. doi:10.1002/mds.23054

156. Messina D., Cerasa A., Condino F., et al. Patterns of brain atrophy in Parkinson’s disease, progressive supranuclear palsy and multiple system atrophy. *Parkinsonism Relat Disord*. 2011;17(3):172-176. doi:10.1016/j.parkreldis.2010.12.010

157. Ebentheuer J, Canelo M, Trautmann E, Trenkwalder C. Substantia nigra echogenicity in progressive supranuclear palsy. *Mov Disord*. 2010;25(6):773-777. doi:10.1002/mds.22981

158. Wang J., Wai Y., Lin W.-Y., et al. Microstructural changes in patients with progressive supranuclear palsy: A diffusion tensor imaging study. *J Magn Reson Imaging*. 2010;32(1):69-75. doi:10.1002/jmri.22229

159. Roselli F., Pisciotta N.M., Pennelli M., et al. Midbrain SERT in degenerative parkinsonisms: A 123I-FP-CIT SPECT study. *Mov Disord*. 2010;25(12):1853-1859. doi:10.1002/mds.23179

160. Gupta D., Saini J., Kesavadas C., Sarma P.S., Kishore A. Utility of susceptibility-weighted MRI in differentiating Parkinson’s disease and atypical parkinsonism. *Neuroradiology*. 2010;52(12):1087-1094. doi:10.1007/s00234-010-0677-6

161. Gama R.L., Tavora D.G., Bomfim R.C., Silva C.E., de Bruin V.M., de Bruin P.F.C. Sleep disturbances and brain MRI morphometry in Parkinson’s disease, multiple system atrophy and progressive supranuclear palsy - a comparative study. *Parkinsonism Relat Disord*. 2010;16(4):275-279. doi:10.1016/j.parkreldis.2010.01.002

162. Gilman S., Koeppe R.A., Nan B., et al. Cerebral cortical and subcortical cholinergic deficits in parkinsonian syndromes. *Neurology*. 2010;74(18):1416-1423. doi:10.1212/WNL.0b013e3181dc1a55

163. Lehericy S., Hartmann A., Lannuzel A., et al. Magnetic resonance imaging lesion pattern in Guadeloupean parkinsonism is distinct from progressive supranuclear palsy. *Brain*. 2010;133(8):2410-2425. doi:10.1093/brain/awq162

164. Vasconcellos LF, Novis SA, Moreira DM, Rosso AL, Leite AC. Neuroimaging in Parkinsonism: a study with magnetic resonance and spectroscopy as tools in the differential diagnosis. *Arq Neuropsiquiatr*. 2009;67(1):1-6. doi:10.1590/s0004-282x2009000100002

165. Stamelou M, Matusch A, Elmenhorst D, et al. Nigrostriatal upregulation of 5-HT2A receptors correlates with motor dysfunction in progressive supranuclear palsy. *Mov Disord*. 2009;24(8):1170-1175. doi:10.1002/mds.22533

166. Borroni B., Malinverno M., Gardoni F., et al. Tau forms in CSF as a reliable biomarker for progressive supranuclear palsy. *Neurology*. 2008;71(22):1796-1803. doi:10.1212/01.wnl.0000335941.68602.39

167. Eckert T., Tang C., Ma Y., et al. Abnormal metabolic networks in atypical parkinsonism. *Mov Disord*. 2008;23(5):727-733. doi:10.1002/mds.21933

168. Nicoletti G., Tonon C., Lodi R., et al. Apparent diffusion coefficient of the superior cerebellar peduncle differentiates progressive supranuclear palsy from Parkinson’s disease. *Mov Disord*. 2008;23(16):2370-2376. doi:10.1002/mds.22279

169. Rizzo G., Martinelli P., Manners D., et al. Diffusion-weighted brain imaging study of patients with clinical diagnosis of corticobasal degeneration, progressive supranuclear palsy and Parkinson’s disease. *Brain*. 2008;131(10):2690-2700. doi:10.1093/brain/awn195

170. Park H.K., Kim J.S., Im K.C., et al. Functional brain imaging in pure Akinesia with Gait freezing: [18F] FDG PET and [18F] FP-CIT PET analyses. *Mov Disord*. 2009;24(2):237-245. doi:10.1002/mds.22347

171. Herting B, Beuthien-Baumann B, Pottrich K, et al. Prefrontal cortex dysfunction and depression in atypical parkinsonian syndromes. *Mov Disord Off J Mov Disord Soc*. 2007;22(4):490-497.

172. Bartels A.L., Willemsen A.T.M., Kortekaas R., et al. Decreased blood-brain barrier P-glycoprotein function in the progression of Parkinson’s disease, PSP and MSA. *J Neural Transm*. 2008;115(7):1001-1009. doi:10.1007/s00702-008-0030-y

173. Paviour D.C., Price S.L., Jahanshahi M., Lees A.J., Fox N.C. Longitudinal MRI in progressive supranuclear palsy and multiple system atrophy: Rates and regions of atrophy. *Brain*. 2006;129(4):1040-1049. doi:10.1093/brain/awl021

174. Paviour D., Price S.L., Jahanshahi M., Lees A.J., Fox N.C. Regional brain volumes distinguish PSP, MSA-P, and PD: MRI-based clinico-radiological correlations. *Mov Disord*. 2006;21(7):989-996. doi:10.1002/mds.20877

175. Seppi K., Scherfler C., Donnemiller E., et al. Topography of dopamine transporter availability in progressive supranuclear palsy: A voxelwise [123I]beta-CIT SPECT analysis. *Arch Neurol*. 2006;63(8):1154-1160. doi:10.1001/archneur.63.8.1154

176. Nicoletti G., Lodi R., Condino F., et al. Apparent diffusion coefficient measurements of the middle cerebellar peduncle differentiate the Parkinson variant of MSA from Parkinson’s disease and progressive supranuclear palsy. *Brain*. 2006;129(10):2679-2687. doi:10.1093/brain/awl166

177. Blain C.R.V., Barker G.J., Jarosz J.M., et al. Measuring brain stem and cerebellar damage in parkinsonian syndromes using diffusion tensor MRI. *Neurology*. 2006;67(12):2199-2205. doi:10.1212/01.wnl.0000249307.59950.f8

178. Josephs K.A., Whitwell J.L., Dickson D.W., et al. Voxel-based morphometry in autopsy proven PSP and CBD. *Neurobiol Aging*. 2008;29(2):280-289. doi:10.1016/j.neurobiolaging.2006.09.019

179. Juh R., Pae C.-U., Kim T.-S., Lee C.-U., Choe B., Suh T. Cerebral glucose metabolism in corticobasal degeneration comparison with progressive supranuclear palsy using statistical mapping analysis. *Neurosci Lett*. 2005;383(1-2):22-27. doi:10.1016/j.neulet.2005.03.057

180. Paviour D.C., Price S.L., Stevens J.M., Lees A.J., Fox N.C. Quantitative MRI measurement of superior cerebellar peduncle in progressive supranuclear palsy. *Neurology*. 2005;64(4):675-679. doi:10.1212/01.WNL.0000151854.85743.C7

181. Gerhard A., Trender-Gerhard I., Turkheimer F., Quinn N.P., Bhatia K.P., Brooks D.J. In vivo imaging of microglial activation with [11C]-PK11195 PET progresive supranuclear palsy. *Mov Disord*. 2006;21(1):89-93. doi:10.1002/mds.20668

182. Oba H., Yagishita A., Terada H., et al. New and reliable MRI diagnosis for progressive supranuclear palsy. *Neurology*. 2005;64(12):2050-2055. doi:10.1212/01.WNL.0000165960.04422.D0

183. Walter U, Dressler D, Wolters A, Probst T, Grossmann A, Benecke R. Sonographic discrimination of corticobasal degeneration vs progressive supranuclear palsy. *Neurology*. 2004;63(3):504-509. doi:10.1212/01.wnl.0000133006.17909.32

184. Klein R.C., de Jong B.M., de Vries J.J., Leenders K.L. Direct comparison between regional cerebral metabolism in progressive supranuclear palsy and Parkinson’s disease. *Mov Disord*. 2005;20(8):1021-1030. doi:10.1002/mds.20493

185. Mishina M, Ishii K, Mitani K, et al. Midbrain hypometabolism as early diagnostic sign for progressive supranuclear palsy. *Acta Neurol Scand*. 2004;110(2):128-135. doi:10.1111/j.1600-0404.2004.00293.x

186. Taki M., Ishii K., Fukuda T., Kojima Y., Mori E. Evaluation of cortical atrophy between progressive supranuclear palsy and corticobasal degeneration by hemispheric surface display of MR images. *Am J Neuroradiol*. 2004;25(10):1709-1714.

187. Groschel K., Hauser T.-K., Luft A., et al. Magnetic resonance imaging-based volumetry differentiates progressive supranuclear palsy from corticobasal degeneration. *NeuroImage*. 2004;21(2):714-724. doi:10.1016/j.neuroimage.2003.09.070

188. Brenneis C., Seppi K., Schocke M., Benke T., Wenning G.K., Poewe W. Voxel based morphometry reveals a distinct pattern of frontal atrophy in progressive supranuclear palsy. *J Neurol Neurosurg Psychiatry*. 2004;75(2):246-249.

189. Eckert T, Sailer M, Kaufmann J, et al. Differentiation of idiopathic Parkinson’s disease, multiple system atrophy, progressive supranuclear palsy, and healthy controls using magnetization transfer imaging. *Neuroimage*. 2004;21(1):229-235. doi:10.1016/j.neuroimage.2003.08.028

190. Walter U, Niehaus L, Probst T, Benecke R, Meyer BU, Dressler D. Brain parenchyma sonography discriminates Parkinson’s disease and atypical parkinsonian syndromes. *Neurology*. 2003;60(1):74-77. doi:10.1212/wnl.60.1.74

191. Kato N, Arai K, Hattori T. Study of the rostral midbrain atrophy in progressive supranuclear palsy. *J Neurol Sci*. 2003;210(1-2):57-60. doi:10.1016/s0022-510x(03)00014-5

192. Juh R., Kim J., Moon D., Choe B., Suh T. Different metabolic patterns analysis of Parkinsonism on the 18F-FDG PET. *Eur J Radiol*. 2004;51(3):223-233. doi:10.1016/S0720-048X%2803%2900214-6

193. Hosaka K, Ishii K, Sakamoto S, et al. Voxel-based comparison of regional cerebral glucose metabolism between PSP and corticobasal degeneration. *J Neurol Sci*. 2002;199(1-2):67-71. doi:10.1016/s0022-510x(02)00102-8

194. Yekhlef F., Ballan G., Macia F., Delmer O., Sourgen C., Tison F. Routine MRI for the differential diagnosis of Parkinson’s disease, MSA, PSP, and CBD. *J Neural Transm*. 2003;110(2):151-169. doi:10.1007/s00702-002-0785-5

195. Zhang L., Murata Y., Ishida R., Saitoh Y., Mizusawa H., Shibuya H. Differentiating between progressive supranuclear palsy and corticobasal degeneration by brain perfusion SPET. *Nucl Med Commun*. 2001;22(7):767-772. doi:10.1097/00006231-200107000-00007

196. Warmuth-Metz M., Naumann M., Csoti I., Solymosi L. Measurement of the midbrain diameter on routine magnetic resonance imaging: A simple and accurate method of differentiating between Parkinson disease and progressive supranuclear palsy. *Arch Neurol*. 2001;58(7):1076-1079. doi:10.1001/archneur.58.7.1076

197. Asato R, Akiguchi I, Masunaga S, Hashimoto N. Magnetic resonance imaging distinguishes progressive supranuclear palsy from multiple system atrophy. *J Neural Transm*. 2000;107(12):1427-1436. doi:10.1007/s007020070006

198. Treseder S.A., Smith L.A., Jenner P. Voxel-based distribution of metabolic impairment in corticobasal degeneration. *Mov Disord*. 2000;15(5):894-904. doi:10.1002/1531-8257%28200009%2915:5%3C894::AID-MDS1021%3E3.0.CO;2-S

199. Garraux G., Salmon E., Degueldre C., Lemaire C., Laureys S., Franck G. Comparison of impaired subcortico-frontal metabolic networks in normal aging, subcortico-frontal dementia, and cortical frontal dementia. *NeuroImage*. 1999;10(2):149-162. doi:10.1006/nimg.1999.0463

200. Schulz J.B., Skalej M., Wedekind D., et al. Magnetic resonance imaging-based volumetry differentiates idiopathic Parkinson’s syndrome from multiple system atrophy and progressive supranuclear palsy. *Ann Neurol*. 1999;45(1):65-74.

201. Tedeschi G., Litvan I., Bonavita S., et al. Proton magnetic resonance spectroscopic imaging in progressive supranuclear palsy, Parkinson’s disease and corticobasal degeneration. *Brain*. 1997;120(9):1541-1552. doi:10.1093/brain/120.9.1541

202. Karbe H, Holthoff V, Huber M, et al. Positron emission tomography in degenerative disorders of the dopaminergic system. *J Neural Transm Park Dis Dement Sect*. 1992;4(2):121-130.

203. Stern MB, Braffman BH, Skolnick BE, Hurtig HI, Grossman RI. Magnetic resonance imaging in Parkinson’s disease and parkinsonian syndromes. *Neurology*. 1989;39(11):1524-1526. doi:10.1212/wnl.39.11.1524

204. Foster N.L., Gilman S., Berent S., Morin E.M., Brown M.B., Koeppe R.A. Cerebral hypometabolism in progressive supranuclear palsy studied with positron emission tomography. *Ann Neurol*. 1988;24(3):399-406. doi:10.1002/ana.410240308

205. Abe S., Miyasaka K., Tashiro K., Takei H., Isu T., Tsuru M. Evaluation of the brainstem with high-resolution CT in cerebellar atrophic processes. *AJNR Am J Neuroradiol*. 1983;4(3):446-449.

206. Onder H., Kocer B.G., Turan A., Comoglu S. The utility of quantitative MRI parameters in discriminating progressive supranuclear palsy from Parkinson’s disease. *Neurol Res*. 2023;((Onder, Kocer, Comoglu) Neurology Clinic, Diskapi Yildirim Beyazit Training and Research Hospital, Ankara, Turkey(Turan) Radiology Clinic, Diskapi Yildirim Beyazit Training and Research Hospital, Ankara, Turkey). doi:10.1080/01616412.2023.2203612

207. Shir D., Thu Pham N.T., Botha H., et al. Clinicoradiologic and Neuropathologic Evaluation of Corticobasal Syndrome. *Neurology*. 2023;((Shir, Botha, Ali, Knopman, Petersen, Boeve, Josephs, Graff-Radford) Department of Neurology, Mayo Clinic, Rochester, United States(Thu Pham, Whitwell) Department of Radiology, Mayo Clinic, Rochester, United States(Koga, Kouri, Murray, Dickson) Department). doi:10.1212/WNL.0000000000207397

208. Muller S.J., Khadhraoui E., Hansen N., et al. Brainstem atrophy in dementia with Lewy bodies compared with progressive supranuclear palsy and Parkinson’s disease on MRI. *BMC Neurol*. 2023;23(1):114. doi:10.1186/s12883-023-03151-4

209. Miyata M., Kakeda S., Yoneda T., et al. Superior cerebellar peduncle atrophy of progressive supranuclear palsy on phase difference enhanced imaging: a comparison with Parkinson’s disease. *Neuroradiology*. 2023;65(4):719-727. doi:10.1007/s00234-023-03119-8

210. Onder H., Kocer B., Turan A., Kertmen H., Comoglu S. The overlap in neuroimaging findings between idiopathic normal pressure hydrocephalus and progressive supranuclear palsy. *Ann Indian Acad Neurol*. 2022;25(6):1087-1091. doi:10.4103/aian.aian_208_22

211. Compta Y, Painous C, Soto M, et al. Combined CSF α-SYN RT-QuIC, CSF NFL and midbrain-pons planimetry in degenerative parkinsonisms: From bedside to bench, and back again. *Park Relat Disord*. 2022;99:33-41. doi:10.1016/j.parkreldis.2022.05.006

212. Illan-Gala I., Nigro S., Vandevrede L., et al. Diagnostic Accuracy of Magnetic Resonance Imaging Measures of Brain Atrophy Across the Spectrum of Progressive Supranuclear Palsy and Corticobasal Degeneration. *JAMA Netw Open*. 2022;5(4):E229588. doi:10.1001/jamanetworkopen.2022.9588

213. Madetko N., Alster P., Kutylowski M., et al. Is MRPI 2.0 More Useful than MRPI and M/P Ratio in Differential Diagnosis of PSP-P with Other Atypical Parkinsonisms? *J Clin Med*. 2022;11(10):2701. doi:10.3390/jcm11102701

214. Luca A., Nicoletti A., Donzuso G., et al. Phonemic Verbal Fluency and Midbrain Atrophy in Progressive Supranuclear Palsy. *J Alzheimers Dis JAD*. 2021;((Donzuso, Terravecchia, Cicero, D’Agate, Rascuna, Manna, Mostile, Zappia) Dipartimento di Scienze Mediche, Chirurgiche e Tecnologie Avanzate “G.F. Ingrassia,” University of Catania, Catania, Italy). doi:10.3233/JAD-210023

215. Virhammar J., Blohme H., Nyholm D., Georgiopoulos C., Fallmar D. Midbrain area and the hummingbird sign from brain MRI in progressive supranuclear palsy and idiopathic normal pressure hydrocephalus. *J Neuroimaging*. 2022;32(1):90-96. doi:10.1111/jon.12932

216. Grijalva R.M., Pham N.T.T., Huang Q., et al. Brainstem Biomarkers of Clinical Variant and Pathology in Progressive Supranuclear Palsy. *Mov Disord*. 2022;37(4):702-712. doi:10.1002/mds.28901

217. Kannenberg S., Caspers J., Dinkelbach L., et al. Investigating the 1-year decline in midbrain-to-pons ratio in the differential diagnosis of PSP and IPD. *J Neurol*. 2021;268(4):1526-1532. doi:10.1007/s00415-020-10327-2

218. Heim B, Mangesius S, Krismer F, et al. Diagnostic accuracy of MR planimetry in clinically unclassifiable parkinsonism. *Parkinsonism Relat Disord*. 2021;82:87-91. doi:10.1016/j.parkreldis.2020.11.019

219. Herwig A., Agic A., Huppertz H.-J., et al. Differentiating Progressive Supranuclear Palsy and Parkinson’s Disease With Head-Mounted Displays. *Front Neurol*. 2021;12((Herwig) Department of Psychology, Clinical Psychology and Psychotherapy, University of Bremen, Bremen, Germany(Herwig, Schneider) Department of Psychology, Neuro-Cognitive Psychology, and Cognitive Interaction Technology (CITEC), Bielefeld University, Bi):791366. doi:10.3389/fneur.2021.791366

220. Janarthanan V., Nadhamuni K., Rajakumar S., Padmanaban E., Amirthalingam U., Achantani Y. Accuracy of Magnetic Resonance Parkinsonism Index in Differentiating Progressive Supranuclear Palsy from Parkinson’s Disease among South Indian Population: A Retrospective Case Control Study. *Indian J Radiol Imaging*. 2021;31(3):596-600. doi:10.1055/s-0041-1736402

221. Cooperrider J., Bluett B., Jones S.E. Methods and utility of quantitative brainstem measurements in progressive supranuclear palsy versus Parkinson’s disease in a routine clinical setting. *Clin Park Relat Disord*. 2020;3((Cooperrider) Cleveland Clinic Lerner College of Medicine, 9980 Carnegie Ave, Cleveland, OH 44195, United States(Bluett, Jones) Cleveland Clinic Imaging Institute, 9500 Euclid Ave, Cleveland, OH 44195, United States(Bluett) Stanford University Department):100033. doi:10.1016/j.prdoa.2020.100033

222. Oktay C., Ozkaynak S.S., Eseroglu E., Karaali K. Contribution of the Mesencephalon Indices to Differential Diagnosis of Parkinsonian Disorders. *Can Assoc Radiol J J Assoc Can Radiol*. 2020;((Oktay, Karaali) Department of Radiology, Akdeniz University School of Medicine, Antalya, Turkey(Ozkaynak) Department of Neurology, Akdeniz University School of Medicine, Antalya, Turkey(Eseroglu) Department of Public Health, Gazi University School of Med):846537119888411. doi:10.1177/0846537119888411

223. Ugga L., Cuocolo R., Cocozza S., et al. Magnetic resonance parkinsonism indices and interpeduncular angle in idiopathic normal pressure hydrocephalus and progressive supranuclear palsy. *Neuroradiology*. 2020;62(12):1657-1665. doi:10.1007/s00234-020-02500-1

224. Nigro S., Antonini A., Vaillancourt D.E., et al. Automated MRI Classification in Progressive Supranuclear Palsy: A Large International Cohort Study. *Mov Disord*. 2020;35(6):976-983. doi:10.1002/mds.28007

225. Picillo M., Tepedino M.F., Abate F., et al. Midbrain MRI assessments in progressive supranuclear palsy subtypes. *J Neurol Neurosurg Psychiatry*. 2020;91(1):98-103. doi:10.1136/jnnp-2019-321354

226. Morelli M., Vescio B., Nigro S., et al. Refining initial diagnosis of Parkinson’s disease after follow-up: A 4-year prospective clinical and magnetic resonance imaging study. *Mov Disord*. 2019;34(4):487-495. doi:10.1002/mds.27621

227. Ahn JH, Kim M, Kim JS, et al. Midbrain atrophy in patients with presymptomatic progressive supranuclear palsy-Richardson’s syndrome. *Parkinsonism Relat Disord*. 2019;66(9513583):80-86. doi:10.1016/j.parkreldis.2019.07.009

228. Nakahara K., Nakane S., Kitajima M., Masuda-Narita T., Matsuo H., Ando Y. Diagnostic accuracy of MRI parameters in pure akinesia with gait freezing. *J Neurol*. 2019;((Nakahara, Nakane, Ando) Department of Neurology, Graduate School of Medical Sciences, Kumamoto University, 1-1-1 Honjo, Chuo-ku, Kumamoto, 860-8556, Japan(Nakane) Department of Molecular Neurology and Therapeutics, Kumamoto University Hospital, 1-1-1 Hon). doi:10.1007/s00415-019-09635-z

229. Sako W., Abe T., Haji S., et al. “One line”: A method for differential diagnosis of parkinsonian syndromes. *Acta Neurol Scand*. 2019;140(3):229-235. doi:10.1111/ane.13136

230. Eraslan C., Acarer A., Guneyli S., et al. MRI evaluation of progressive supranuclear palsy: differentiation from Parkinson’s disease and multiple system atrophy. *Neurol Res*. 2019;41(2):110-117. doi:10.1080/01616412.2018.1541115

231. Constantinides V.C., Paraskevas G.P., Velonakis G., Toulas P., Stefanis L., Kapaki E. Midbrain morphology in idiopathic normal pressure hydrocephalus. A progressive supranuclear palsy mimic. *Acta Neurol Scand*. 2019;((Constantinides, Paraskevas, Stefanis, Kapaki) 1st Department of Neurology, National and Kapodistrian University of Athens, School of Medicine, Eginition Hospital, Athens, United States(Velonakis, Toulas) Research Unit of Radiology, 2nd Department of Radi). doi:10.1111/ane.13205

232. Mueller C., Hussl A., Krismer F., et al. The diagnostic accuracy of the hummingbird and morning glory sign in patients with neurodegenerative parkinsonism. *Parkinsonism Relat Disord*. 2018;54((Mueller, Hussl, Krismer, Heim, Mahlknecht, Nocker, Scherfler, Mair, Wenning, Poewe, Seppi) Department of Neurology, Innsbruck Medical University, Austria(Esterhammer, Schocke) Department of Radiology 1, Innsbruck Medical University, Austria(Scherfler, Sc):90-94. doi:10.1016/j.parkreldis.2018.04.005

233. Constantinides V.C., Paraskevas G.P., Velonakis G., Toulas P., Stamboulis E., Kapaki E. MRI planimetry and magnetic resonance parkinsonism index in the differential diagnosis of patients with Parkinsonism. *Am J Neuroradiol*. 2018;39(6):1047-1051. doi:10.3174/ajnr.A5618

234. Morelli M., Nigro S., Quattrone A., et al. A new MR imaging index for differentiation of progressive supranuclear palsy-parkinsonism from Parkinson’s disease. *Parkinsonism Relat Disord*. 2018;54((Quattrone, Gulla) Neuroscience Centre, Magna Graecia University, Catanzaro, Italy(Quattrone, Morelli, Nigro, Arabia, Nicoletti, Nistico, Salsone, Novellino, Vaccaro, Chiriaco, Rocca, Caracciolo) Neuroimaging Research Unit, Institute of Molecular Bioimagi):3-8. doi:10.1016/j.parkreldis.2018.07.016

235. Kim B.C., Choi S.-M., Choi K.-H., et al. MRI measurements of brainstem structures in patients with vascular parkinsonism, progressive supranuclear palsy, and Parkinson’s disease. *Neurol Sci*. 2017;38(4):627-633. doi:10.1007/s10072-017-2812-1

236. Silsby M., Tweedie-Cullen R.Y., Murray C.R., Halliday G.M., Hodges J.R., Burrell J.R. The midbrain-to-pons ratio distinguishes progressive supranuclear palsy from non-fluent primary progressive aphasias. *Eur J Neurol*. 2017;24(7):956-965. doi:10.1111/ene.13314

237. Nizamani W.M., Mubarak F., Barakzai M.D., Ahmed M.S. Role of magnetic resonance planimetry and magnetic resonance parkinsonism index in discriminating Parkinson’s disease and progressive supranuclear palsy: A retrospective study based on 1.5 and 3 T MRI. *Int J Gen Med*. 2017;10((Nizamani, Mubarak, Barakzai) Department of Radiology, Aga Khan University Hospital, Karachi, Pakistan(Ahmed) Department of Radiology, Ziauddin University Hospital, Karachi, Pakistan):375-384. doi:10.2147/IJGM.S134297

238. Mangesius S., Hussl A., Krismer F., et al. MR planimetry in neurodegenerative parkinsonism yields high diagnostic accuracy for PSP. *Parkinsonism Relat Disord*. 2018;46((Mangesius, Hussl, Krismer, Mahlknecht, Reiter, Tagwercher, Djamshidian, Wenning, Muller, Scherfler, Poewe, Seppi) Department of Neurology, Medical University Innsbruck, Innsbruck, Austria(Mangesius, Schocke, Esterhammer, Gizewski) Department of Neuroradi):47-55. doi:10.1016/j.parkreldis.2017.10.020

239. Nigro S, Arabia G, Antonini A, et al. Magnetic Resonance Parkinsonism Index: diagnostic accuracy of a fully automated algorithm in comparison with the manual measurement in a large Italian multicentre study in patients with progressive supranuclear palsy. *Eur Radiol*. 2017;27(6):2665-2675. doi:10.1007/s00330-016-4622-x

240. Moller L., Kassubek J., Sudmeyer M., et al. Manual MRI morphometry in Parkinsonian syndromes. *Mov Disord*. 2017;32(5):778-782. doi:10.1002/mds.26921

241. Mostile G., Nicoletti A., Cicero C.E., et al. Magnetic resonance parkinsonism index in progressive supranuclear palsy and vascular parkinsonism. *Neurol Sci*. 2016;37(4):591-595. doi:10.1007/s10072-016-2489-x

242. Zanigni S., Calandra-Buonaura G., Manners D.N., et al. Accuracy of MR markers for differentiating Progressive Supranuclear Palsy from Parkinson’s disease. *NeuroImage Clin*. 2016;11((Zanigni, Manners, Testa, Evangelisti, Gramegna, Bianchini, Lodi, Tonon) Functional MR Unit, Policlinico S. Orsola Malpighi, Department of Biomedical and NeuroMotor Sciences (DiBiNeM), University of Bologna, Via Massarenti 9, Bologna 40138, Italy(Zanigni,):736-742. doi:10.1016/j.nicl.2016.05.016

243. Pasha SA, Yadav R, Ganeshan M, et al. Correlation between qualitative balance indices, dynamic posturography and structural brain imaging in patients with progressive supranuclear palsy and its subtypes. *Neurol India*. 2016;64(4):633-639. doi:10.4103/0028-3886.185417

244. Sankhla C.S., Patil K.B., Sawant N., Gupta S. Diagnostic accuracy of Magnetic Resonance Parkinsonism Index in differentiating progressive supranuclear palsy from Parkinson’s disease and controls in Indian patients. *Neurol India*. 2016;64(2):239-245. doi:10.4103/0028-3886.177611

245. Tipton PW, Konno T, Broderick DF, Dickson DW, Wszolek ZK. Cerebral peduncle angle: Unreliable in differentiating progressive supranuclear palsy from other neurodegenerative diseases. *Park Relat Disord*. 2016;32:31-35. doi:10.1016/j.parkreldis.2016.08.009

246. Hwang M., Yang H., Kim Y., et al. Differential progression of midbrain atrophy in parkinsonism: Longitudinal MRI study. *Neurodegener Dis*. 2016;17(1):31-37. doi:10.1159/000448174

247. Owens E., Krecke K., Ahlskog J.E., et al. Highly specific radiographic marker predates clinical diagnosis in progressive supranuclear palsy. *Parkinsonism Relat Disord*. 2016;28((Owens, Ahlskog, Fealey, Hassan, Josephs, Klassen, Matsumoto, Bower) Mayo Clinic Department of Neurology, 200 Second St. SW, Rochester, MN 55905, United States(Krecke) Mayo Clinic Department of Neuroradiology, 200 Second St. SW, Rochester, MN 55905, Unite):107-111. doi:10.1016/j.parkreldis.2016.05.006

248. Fatterpekar G.M., Dietrich A., Pantano P., et al. Cerebral peduncle angle: An objective criterion for assessing progressive supranuclear palsy richardson syndrome. *Am J Roentgenol*. 2015;205(2):386-391. doi:10.2214/AJR.14.12724

249. Kaasinen V., Kangassalo N., Gardberg M., et al. Midbrain-to-pons ratio in autopsy-confirmed progressive supranuclear palsy: replication in an independent cohort. *Neurol Sci*. 2015;36(7):1251-1253. doi:10.1007/s10072-015-2184-3

250. Whitwell J.L., Jack C.R., Parisi J.E., et al. Midbrain atrophy is not a biomarker of progressive supranuclear palsy pathology. *Eur J Neurol*. 2013;20(10):1417-1422. doi:10.1111/ene.12212

251. Massey L.A., Jager H.R., Paviour D.C., et al. The midbrain to pons ratio: A simple and specific MRI sign of progressive supranuclear palsy. *Neurology*. 2013;80(20):1856-1861. doi:10.1212/WNL.0b013e318292a2d2

252. Massey LA, Micallef C, Paviour DC, et al. Conventional magnetic resonance imaging in confirmed progressive supranuclear palsy and multiple system atrophy. *Mov Disord*. 2012;27(14):1754-1762. doi:10.1002/mds.24968

253. Whitwell J.L., Xu J., Mandrekar J.N., Gunter J.L., Jack Jr. C.R., Josephs K.A. Rates of brain atrophy and clinical decline over 6 and 12-month intervals in PSP: Determining sample size for treatment trials. *Parkinsonism Relat Disord*. 2012;18(3):252-256. doi:10.1016/j.parkreldis.2011.10.013

254. Morelli M., Arabia G., Salsone M., et al. Accuracy of magnetic resonance parkinsonism index for differentiation of progressive supranuclear palsy from probable or possible Parkinson disease. *Mov Disord*. 2011;26(3):527-533. doi:10.1002/mds.23529

255. Looi J.C.L., Macfarlane M.D., Walterfang M., et al. Morphometric analysis of subcortical structures in progressive supranuclear palsy: In vivo evidence of neostriatal and mesencephalic atrophy. *Psychiatry Res - Neuroimaging*. 2011;194(2):163-175. doi:10.1016/j.pscychresns.2011.07.013

256. Choi S.-M., Kim B.C., Nam T.-S., et al. Midbrain atrophy in vascular parkinsonism. *Eur Neurol*. 2011;65(5):296-301. doi:10.1159/000326907

257. Gama R.L., Tavora D.F.G., Bomfim R.C., Silva C.E., de Bruin V.M., de Bruin P.F. Morphometry MRI in the differential diagnosis of parkinsonian syndromes. *Arq Neuropsiquiatr*. 2010;68(3):333-338. doi:10.1590/S0004-282X2010000300001

258. Longoni G, Agosta F, Kostić VS, et al. MRI measurements of brainstem structures in patients with Richardson’s syndrome, progressive supranuclear palsy-parkinsonism, and Parkinson’s disease. *Mov Disord*. 2011;26(2):247-255. doi:10.1002/mds.23293

259. Emam A.T., El-Sayed M.A., Awad F.M. Progressive supranuclear palsy: Brainstem measurement and its clinical implication. *Egypt J Neurol Psychiatry Neurosurg*. 2010;47(3):355-360.

260. Hussl A., Mahlknecht P., Scherfler C., et al. Diagnostic accuracy of the magnetic resonance Parkinsonism index and the midbrain-to-pontine area ratio to differentiate progressive supranuclear palsy from Parkinson’s disease and the Parkinson variant of multiple system atrophy. *Mov Disord*. 2010;25(14):2444-2449. doi:10.1002/mds.23351

261. Rohrer J.D., Paviour D., Bronstein A.M., O’Sullivan S.S., Lees A., Warren J.D. Progressive supranuclear palsy syndrome presenting as progressive nonfluent aphasia: A neuropsychological and neuroimaging analysis. *Mov Disord*. 2010;25(2):179-188. doi:10.1002/mds.22946

262. Sung Y.-H., Park K.-H., Lee Y.-B., et al. Midbrain atrophy in subcortical ischemic vascular dementia. *J Neurol*. 2009;256(12):1997-2002. doi:10.1007/s00415-009-5226-z

263. Slowinski J., Imamura A., Uitti R.J., et al. MR imaging of brainstem atrophy in progressive supranuclear palsy. *J Neurol*. 2008;255(1):37-44. doi:10.1007/s00415-007-0656-y

264. Quattrone A., Nicoletti G., Messina D., et al. MR imaging index for differentiation of progressive supranuclear palsy from Parkinson disease and the Parkinson variant of multiple system atrophy. *Radiology*. 2008;246(1):214-221. doi:10.1148/radiol.2453061703

265. Cosottini M, Ceravolo R, Faggioni L, et al. Assessment of midbrain atrophy in patients with progressive supranuclear palsy with routine magnetic resonance imaging. *Acta Neurol Scand*. 2007;116(1):37-42. doi:10.1111/j.1600-0404.2006.00767.x

266. Barsottini O.G.P., Ferraz H.B., Maia Jr. A.C.M., Silva C.J., Rocha A.J. Differentiation of Parkinson’s disease and progressive supranuclear palsy with magnetic resonance imaging: The first Brazilian experience. *Parkinsonism Relat Disord*. 2007;13(7):389-393. doi:10.1016/j.parkreldis.2006.12.011

267. Groschel K., Kastrup A., Litvan I., Schulz J.B. Penguins and hummingbirds: Midbrain atrophy in progressive supranuclear palsy. *Neurology*. 2006;66(6):949-950. doi:10.1212/01.wnl.0000203342.77115.bf

268. Strecker K., Schneider J.P., Barthel H., et al. Profound midbrain atrophy in patients with Wilson’s disease and neurological symptoms? *J Neurol*. 2006;253(8):1024-1029. doi:10.1007/s00415-006-0151-x

269. Righini A., Antonini A., De Notaris R., et al. MR imaging of the superior profile of the midbrain: Differential diagnosis between progressive supranuclear palsy and Parkinson disease. *Am J Neuroradiol*. 2004;25(6):927-932.

270. Upadhyay N., Suppa A., Piattella M.C., et al. Functional disconnection of thalamic and cerebellar dentate nucleus networks in progressive supranuclear palsy and corticobasal syndrome. *Parkinsonism Relat Disord*. 2017;39:52-57. doi:10.1016/j.parkreldis.2017.03.008

271. Zwergal A., La Fougere C., Lorenzl S., et al. Postural imbalance and falls in PSP correlate with functional pathology of the thalamus. *Neurology*. 2011;77(2):101-109. doi:10.1212/WNL.0b013e318223c79d
